# CD45⁺ hybrid circulating cells may reflect tumor-immune interactions and serve as transcriptomic indicators of metastatic potential in prostate cancer

**DOI:** 10.7150/thno.122226

**Published:** 2026-01-01

**Authors:** Baek Gil Kim, Yeonsue Jang, Min Gyu Kim, Dongwook Song, Jungchan Jung, Jihee Jung, Ayoung Yoo, Jongsoo Lee, Nam Hoon Cho, Hyeong Jung Woo, Woon-Hae Kim, Hyun Young Shin, Minseok S. Kim, Hyun Ho Han, Jae Young Joung

**Affiliations:** 1 Department of Urology, Urological Science Institute, Yonsei University College of Medicine, 50-1 Yonsei-ro, Seodaemun-gu, Seoul 03722, Republic of Korea.; 2 Brain Korea 21 Plus Project for Medical Science, Yonsei University College of Medicine, 50-1 Yonsei-ro, Seodaemun-gu, Seoul 03722, Republic of Korea.; 3 Center for Urologic Cancer, National Cancer Center, 323 Ilsan-ro, Ilsandong-gu, Goyang-si, Gyeonggi-do 10408, Republic of Korea.; 4 Department of Pathology, Yonsei University College of Medicine, 50-1 Yonsei-ro, Seodaemun-gu, Seoul 03722, Republic of Korea.; 5 Department of New Biology, Daegu Gyeongbuk Institute of Science and Technology (DGIST), 333 Techno Jungang-daero, Hyeonpung-eup, Dalseong-gun, Daegu 42988, Republic of Korea.; 6 CTCELLS Inc., 216, Gaepo-ro, Gangnam-gu, Seoul 06307, Republic of Korea.

**Keywords:** Prostate cancer, Circulating hybrid cells, Single-cell RNA sequencing, Metastasis prediction, CD45⁺ KRT18⁺ hybrid cells, Tumor-immune interaction

## Abstract

**Rationale:** Circulating hybrid cells expressing both epithelial and immune markers have emerged as indicators of dynamic tumor-immune interactions. This study aimed to characterize circulating hybrid cells co-expressing *KRT18* (pan-cytokeratin) and *PTPRC* (CD45), termed KP_Pos, in metastatic prostate cancer (mPCa), and to assess their molecular features, tumor microenvironmental (TME) origins, and clinical relevance.

**Methods:** Imaging mass cytometry (IMC) was used to examine spatial relationships between CK⁺ tumor and CD45⁺ immune cells in metastatic prostate tissues. Single-cell RNA sequencing (scRNA-seq) datasets from mPCa were analyzed to identify KP_Pos cells and characterize their transcriptional heterogeneity across epithelial and immune lineages. Differentially expressed genes (DEGs) between KP_Pos and other cells were used to generate predictive gene signatures. Random forest (RF) and extreme gradient boosting (XGB) models were applied to evaluate metastatic classification performance, and high-performing signatures were validated in bulk RNA-seq datasets and correlated with clinical parameters.

**Results:** IMC revealed frequent spatial proximity between tumor and immune compartments, supporting a TME-derived hybrid phenotype. KP_Pos cells were detected across multiple immune and epithelial clusters, showing heterogeneity and enrichment of immune response and epithelial-mesenchymal transition (EMT)-related genes. Machine learning-based classifiers using KP_Pos-derived DEGs achieved high predictive accuracy (AUC ≥ 0.7) for metastasis, and selected combinations further improved performance in internal validation sets. Signature scores significantly correlated with PSA and Gleason grade, and CD45⁺ hybrid circulating cells were more abundant in patients with advanced disease burden.

**Conclusions:** CD45⁺ KRT18⁺ hybrid circulating cells (KP_Pos) represent biologically distinct populations shaped by tumor-immune interactions within the TME. Their transcriptomic features and derived gene signatures may serve as biomarkers of metastatic potential and indicators of disease progression in prostate cancer. However, their causal role in metastasis and impact on survival remain to be determined.

## Introduction

Prostate cancer (PCa) is the most frequently diagnosed cancer and the fifth leading cause of cancer-related death among men worldwide, with over 1.4 million new cases and approximately 375,000 deaths each year 1. The distinction between localized and metastatic disease is a pivotal determinant of therapeutic decision-making, as metastatic PCa is associated with markedly worse outcomes and limited curative options 2. Early and accurate prediction of metastasis could thus play a crucial role in improving survival and reducing treatment-related morbidity by enabling timely and tailored interventions 3.

Although many prostate cancers are initially indolent, a considerable proportion progress to aggressive phenotypes, and up to 20% of patients present with metastases at diagnosis 4. Furthermore, among patients with localized disease, recurrence and subsequent progression to distant metastasis remain a significant clinical concern 5. While recent advances in systemic therapies—including androgen receptor signaling inhibitors, radionuclide therapies, and immunotherapy—have transformed the management of metastatic PCa, their success hinges upon accurate risk stratification at an early stage 6. Moreover, the advent of PSMA PET-CT has significantly altered diagnostic and therapeutic decision-making in prostate cancer, further underscoring the need for biomarkers that can complement advanced imaging modalities 7.

In this context, the identification of robust biomarkers or molecular signatures capable of predicting metastatic potential is a pressing need. Circulating tumor cells (CTCs) have emerged as a promising non-invasive biomarker of metastasis in various cancers 8. Traditionally, CTCs are defined by the expression of epithelial markers such as cytokeratins and the absence of the pan-leukocyte marker CD45. However, recent studies have challenged this classical dichotomy by reporting CTC-like cells that co-express both epithelial and immune markers, including CD45. These hybrid phenotypes may result from epithelial-mesenchymal transition (EMT), tumor-immune cell fusion, or immune mimicry mechanisms 9-11. One proposed mechanism underlying the emergence of such hybrid CTC-like cells is spontaneous fusion between neoplastic epithelial cells and tumor-associated macrophages. This fusion gives rise to progeny that co-express hematopoietic and epithelial markers and exhibit enhanced migratory capacity, immune evasion, and metastatic plasticity, as demonstrated in both murine models and human tumors 12.

Such phenotypically hybrid cells have been observed in several malignancies and are often enriched in patients with metastatic disease 13-15. In prostate cancer, while CTCs are generally CD45-, recent findings suggest that CD45 expression in circulating epithelial-like cells may be associated with increased metastatic potential 11. A similar phenomenon has been described in breast cancer, where CD45-overexpressing tumor cells exhibit enhanced migratory and immune-evasive properties 16. These findings indicate that the appearance of atypically expressed cells in circulation may serve as a sensitive indicator of metastatic dissemination.

Concurrently, transcriptomic analysis has revolutionized the field of precision oncology by enabling comprehensive characterization of the tumor microenvironment and metastatic programs 17. In parallel, genetic testing is now increasingly recommended in prostate cancer to refine risk stratification and guide precision treatment decisions 18. Bulk and single-cell RNA sequencing allow for the identification of gene expression signatures linked to invasion, EMT, immune evasion, and metastasis 19, 20. Such transcriptome-based approaches have shown promise in stratifying patients, predicting treatment response, and uncovering mechanisms of progression 21, 22.

In this study, we hypothesized that circulating tumor cell (CTC)-like populations co-expressing epithelial and immune markers may reflect underlying metastatic programs shaped by the tumor microenvironment. To test this, we conducted an integrative analysis that combines single-cell RNA sequencing (scRNA-seq)-based signature discovery from primary prostate tumors with validation in bulk RNA-seq datasets from metastatic prostate cancer tissues. By evaluating the predictive value of these transcriptomic signatures for metastatic status, we aim to identify robust biomarkers associated with tumor aggressiveness. We note that such associations do not imply direct causation, which would require functional validation beyond the scope of this study. This approach provides a foundation for a novel biomarker framework aligned with personalized and metastasis-informed management strategies in prostate cancer.

## Materials and Methods

### Sample acquisition

Peripheral blood mononuclear cells (PBMCs) and tissue samples were collected from prostate cancer patients as part of a study approved by the Institutional Review Board of Severance Hospital, Yonsei University College of Medicine (IRB numbers: 4-2022-0710, 4-2021-0276). All participants provided written informed consent after being appropriately informed that their peripheral blood and tissue samples would be used for research purposes. Peripheral blood samples (5 mL) were obtained from 82 patients via venipuncture using a 21-gauge needle and collected into EDTA-coated Vacutainer tubes (Becton, Dickinson and Company, Franklin Lakes, NJ) for circulating cell analysis. Peripheral blood mononuclear cells (PBMCs) were isolated from seven patients with metastatic prostate cancer (M1 stage) for single-cell RNA sequencing (scRNA-seq) analysis. For comparative purposes, biopsy tissues from five additional patients with clinically and radiographically confirmed metastatic prostate cancer (M1 stage) were also collected and processed for scRNA-seq. Because patient consent prohibited paired blood-tissue collection, the PBMC and biopsy cohorts were analyzed independently. In addition, biopsy specimens from 234 prostate cancer patients—including both non-metastatic (M0) and metastatic (M1) cases—were subjected to bulk RNA sequencing.

### Circulating cell isolation

Circulating cell isolation was performed using the CTCeptor system (CTCELLS, Daegu, South Korea), a fully automated Continuous Centrifugal Microfluidics-Circulating Tumor Cell Disc (CCM-CTCD) platform 23, 24. This system employs a rotating microfluidic disc to separate blood components based on their density. A synchronized laser-controlled motor activates an internal valve to release a thin layer enriched with tumor-derived and white blood cells into a designated chamber, where circulating cells are selectively captured through antibody-based surface binding. The isolated cells were subjected to immunofluorescence staining using the following antibodies: anti-pan-cytokeratin (PanCK; eBioscience, San Diego, CA), anti-CD45 (BioLegend, San Diego, CA), and anti-prostate-specific antigen (PSA; Invitrogen, Waltham, MA). Stained cells were imaged and analyzed to distinguish epithelial-derived cells (PanCK⁺/CD45⁻), immune-origin cells (CD45⁺), and hybrid phenotypes (PanCK⁺/CD45⁺), reflecting the cellular heterogeneity of circulating populations in prostate cancer.

### Imaging mass cytometry

Formalin-fixed, paraffin-embedded (FFPE) prostate tissue sections from 21 tissue microarrays (TMAs) of metastatic prostate cancer were deparaffinized, rehydrated, subjected to antigen retrieval, and blocked with 3% BSA. The slides were then incubated with metal-conjugated antibodies labeled using Maxpar X8 Antibody Labeling Kits (Standard BioTools, South San Francisco, CA), purified, and quantified using NanoDrop spectrophotometry (Thermo Fisher Scientific, Waltham, MA), followed by storage at 4°C until use. A Standard BioTools-verified antibody panel targeting tumor and immune-related markers—including cytokeratin, CD45, CD14, and CD16—was applied for staining. Imaging Mass Cytometry (IMC) was performed using the Hyperion Imaging System (Standard BioTools), with laser ablation and data acquisition conducted at JCBio (Seoul, South Korea). Regions of interest (1000 × 1000 µm) were selected based on tissue morphology, and the acquired data were processed using CyTOF software v7.0 (Standard BioTools, South San Francisco, CA). Image quality was assessed prior to exporting the data as multilayer OME-TIFF files, which were analyzed using HALO Imaging Analysis Software (v3.5, Indica Labs, Albuquerque, NM). The Highplex FL module was used for cell segmentation and marker quantification, while spatial tissue analyses—including nearest neighbor and proximity-based interaction mapping—were performed to characterize tumor-immune interactions. Co-registration of serial sections and the generation of density heatmaps enabled detailed spatial visualization of immune and epithelial cell populations. Morphometric quantification of CK⁺CD45⁺ double-positive cells was performed using cell segmentation masks generated in HALO. Cell and cytoplasmic area distributions were binned (10 µm² per bin), and bin-wise percent values were calculated for each group. To enable quantitative comparison across groups, cumulative proportions (“AUC_percent”) were computed as the sum of percent values within the defined small-size range (≤250 µm²), representing the relative enrichment of smaller cells.

### RNA sequencing

#### Single-cell RNA sequencing

Freshly obtained primary tumor biopsy specimens from metastatic prostate cancer patients (M1 stage) were processed to generate single-cell suspensions. Enzymatic dissociation was performed using in-house optimized protocols developed by DCGEN (Seoul, South Korea). Following filtration through a 40 µm cell strainer (Corning, Corning, NY) and viability assessment using an automated cell counter (Countess II, Thermo Fisher Scientific, Waltham, MA), single cells were encapsulated and barcoded using the Chromium Controller system (10x Genomics, Pleasanton, CA) operated at Macrogen (Seoul, South Korea). cDNA synthesis, amplification, and library construction were conducted according to the manufacturer's protocol (10x Genomics). Libraries were quality-checked with a Bioanalyzer (Agilent Technologies, Santa Clara, CA) and a Qubit fluorometer (Thermo Fisher Scientific, Waltham, MA), and sequenced on Illumina NovaSeq 6000 or NextSeq 2000 platforms (Illumina, San Diego, CA). The raw sequencing data were processed using Cell Ranger software (10x Genomics) to generate gene expression matrices for downstream analyses, including tumor microenvironment (TME) characterization and ligand-receptor interaction mapping. Peripheral blood mononuclear cells (PBMCs) were isolated from seven patients with metastatic prostate cancer (M1 stage) and subjected to single-cell RNA sequencing (scRNA-seq) to profile circulating immune-cell transcriptomes. After cell isolation and viability assessment, single-cell encapsulation and barcoding were performed using the Chromium Controller system (10x Genomics, Pleasanton, CA). Library preparation, including cDNA synthesis and amplification, was conducted according to the manufacturer's protocol. Sequencing was performed by Eyeoncell (Gwangju, South Korea) on Illumina NovaSeq 6000 or NextSeq 2000 platforms (Illumina, San Diego, CA). Raw sequencing data were processed using Cell Ranger software (10x Genomics) to generate gene-cell count matrices for downstream analyses.

#### Bulk RNA sequencing

Total RNA was extracted from biopsy tissues using TRIzol reagent (Thermo Fisher Scientific, Waltham, MA) or the RNeasy Mini Kit (Qiagen, Hilden, Germany). RNA integrity was verified using a Bioanalyzer (Agilent Technologies, Santa Clara, CA), and RNA concentrations were measured using a Qubit fluorometer (Thermo Fisher Scientific, Waltham, MA). Only samples with an RNA Integrity Number (RIN) greater than 7.0 were used for library preparation with the TruSeq Stranded mRNA Library Prep Kit (Illumina, San Diego, CA). Sequencing was performed on the Illumina NovaSeq 6000 or NextSeq 2000 platforms (Illumina, San Diego, CA) using 100-150 bp paired-end reads, with a target depth of 20-50 million reads per sample. Raw reads were aligned to the reference genome using STAR or HISAT2, and gene expression was quantified using featureCounts or HTSeq-count.

### Single-cell transcriptomic analysis and differential gene expression

Single-cell RNA-seq data were analyzed using R (v4.3.2) and the Seurat package. For integrated analyses, scRNA-seq datasets from five metastatic prostate cancer cases were merged using the anchor-based integration workflow implemented in Seurat v4. Briefly, each dataset was log-normalized, and the top 2,000 variable features were identified. Integration anchors were calculated using FindIntegrationAnchors with default parameters, and the datasets were aligned into a shared expression space via IntegrateData, which corrects for patient-specific batch effects while preserving biological variability. This integration enabled direct comparison of identical cell types across patients, enhanced the detection of rare populations (e.g., KP_Pos cells), and improved the robustness of downstream clustering, annotation, and differential expression analyses. Cell type annotation was performed using six complementary approaches: (1) SingleR-based methods, including (i) cell-level annotation using the HumanPrimaryCellAtlasData reference, (ii) cluster-level majority voting, defined as assigning the most frequent SingleR-derived cell-type label within each Seurat-defined cluster, and (iii) cluster-to-cell-type mapping; (2) CellTypist-based annotation using the Immune_All_Low pretrained model; (3) canonical marker-based annotation using curated cell type-specific gene sets; and (4) cell subtype-specific marker scoring using lineage-relevant markers for regulatory, cytotoxic, exhausted, memory, and helper T cells, B-cell subsets, macrophages, and epithelial cells. Marker gene sets used for annotation are listed in Supplementary Tables S7-1 (cell types) and S7-2 (cell subtypes). In this study, no single annotation approach was designated as the “gold standard.” Instead, all six methods were applied to capture complementary perspectives on cell identity, acknowledging that each can yield distinct yet biologically meaningful results. For downstream analyses and visualization in the main text, we selected the SingleR cluster-level majority-voting annotation as the representative strategy because it provided clearer cluster separation in two-dimensional embeddings and minimized figure complexity. This choice was made solely for presentation clarity and does not indicate analytical preference or bias. To ensure interpretability and reproducibility, all differential expression analyses and subsequent signature construction steps were based on a unified DEG pool that combined the results from all annotation approaches, thereby incorporating both overlapping and method-specific DEGs. KP_Pos cells were defined as those co-expressing *KRT18* and *PTPRC* (normalized expression > 0.1), with all other cells categorized as “Others.” DEG analysis comparing KP_Pos versus Others was conducted using the Wilcoxon rank-sum test implemented in Seurat's FindMarkers function, applying a log₂ fold-change threshold ≥ 0.25 and requiring expression in at least 10% of cells per group. Significant DEGs were selected based on an adjusted p-value ≤ 0.05. Two DEG strategies were performed: (i) cluster-based analysis within our metastatic prostate cancer scRNA-seq dataset, and (ii) metastasis-specific analysis incorporating benign and localized prostate cancer samples from GSE193337.

### Cell-cell interaction analysis

Cell-cell communication was analyzed using the CellChat R package (v1.6.1). Raw count matrices from Seurat-integrated single-cell RNA-seq data of PBMCs from metastatic prostate cancer patients were merged into a unified Seurat object. Cell types were annotated using SingleR with the HumanPrimaryCellAtlasData reference, and the resulting labels were incorporated into the CellChat metadata. A CellChat object was constructed using the merged count matrix and cell-type annotations and analyzed with the CellChatDB.human ligand-receptor database. Overexpressed genes and interactions were identified using the functions identifyOverExpressedGenes() and identifyOverExpressedInteractions(). Communication probabilities were computed via computeCommunProb() and computeCommunProbPathway(), considering only cell types containing ≥10 cells. The resulting network strengths were aggregated with aggregateNet() and visualized using circular and heatmap layouts. Sender-receiver (incoming/outgoing) signaling analyses were performed for six major immune populations—monocytes, macrophages, T cells, NK cells, B cells, and CMPs—to quantify intercellular signaling strength and relative communication topology.

### Signature-based classification and model evaluation

To identify transcriptomic signatures predictive of metastasis (M0 vs. M1), we implemented a machine learning workflow based on gene expression-derived features. Each signature was evaluated using a three-way data partitioning strategy (training/validation/test) with five different random seeds to ensure robustness and reproducibility. Model training and validation were performed using random forest (RF) and extreme gradient boosting (XGB), and performance was assessed by calculating AUC, PR AUC, accuracy, sensitivity, specificity, precision, and F1-score. To control for overfitting, signatures were categorized into four levels (none, mild, moderate, severe) based on discrepancies between training and test set performance; only signatures classified as None in the overfitting-level analysis were used for downstream analysis. A composite score was calculated by z-transforming PR AUCs from both RF and XGB models and summing them, enabling robust ranking of consistently high-performing signatures. Signatures with AUC ≥ 0.7 in the test set were further applied to bulk RNA-seq data for M-stage (M0 vs. M1) prediction. This cutoff of 0.7 was selected because it is widely regarded as the lower bound for clinically meaningful discrimination, balancing sensitivity and specificity beyond random chance. The threshold has also been frequently adopted in prior biomarker studies to ensure comparability across studies and to exclude weak predictors. Given the class imbalance in our dataset, PR-AUC ≥ 0.7 in the test set was used as the primary criterion, ensuring that only robustly predictive signatures were advanced to downstream analysis. To assess whether predictive performance could be improved through integration, combinations of top-ranked signatures were tested using logistic regression, RF, and XGB with repeated stratified cross-validation across five seeds. Model performance was compared using mean ROC AUC, PR AUC, accuracy, and F1-score, and the final composite models were evaluated for associations with clinical features.

### M0/M1 prediction and performance evaluation

For M-stage classification (M0 vs. M1), model performance was assessed using standard binary classification metrics. Predicted probabilities from RF and XGB models were converted into class labels using a default threshold of 0.5, unless otherwise optimized based on PR AUC. The following evaluation metrics were calculated: accuracy = (TP + TN) / (TP + TN + FP + FN), sensitivity = TP / (TP + FN), specificity = TN / (TN + FP), precision = TP / (TP + FP), and F1-score = 2 × (precision × sensitivity) / (precision + sensitivity). Additionally, area under the receiver operating characteristic curve (ROC AUC) and precision-recall curve (PR AUC) were computed to assess overall discriminative power, particularly under class imbalance. All metrics were averaged across five random seeds for robust comparison between individual signatures and signature combinations.

### Correlation analysis with clinical variables

To assess the clinical relevance of circulating cell phenotypes and gene expression-based prediction scores, we performed correlation analyses with key clinical parameters including age, PSA level, Gleason score, and TNM staging. For circulating cell phenotypes defined by CD45 expression status (CD45⁺ and CD45⁻), Pearson correlation coefficients were calculated and visualized using Microsoft Excel (Microsoft Corporation, Redmond, WA). For combined signature scores derived from top-ranked gene sets, Spearman rank correlation was computed in R using the cor.test() function. Clinical variables were numerically encoded, and results were visualized using ggplot2-based bubble plots, where color scale represented correlation strength and direction, and circle size indicated statistical significance (-log₁₀(p-value)).

### Statistical analysis

All statistical analyses were performed using GraphPad Prism (GraphPad Software, La Jolla, CA) and R (version 4.3.2). Gene expression differences between groups were assessed using two-tailed unpaired Student's t-tests, assuming equal variance unless otherwise specified. Correlation analyses in RNA-seq datasets were conducted using Pearson's correlation coefficient. For correlation between gene signature-based predictions and clinical parameters, Spearman's rank correlation was used when the data were not normally distributed or were ordinal in nature. A p-value less than 0.05 was considered statistically significant. Statistical significance was denoted by asterisks as follows: p ≤ 0.05 (*), p ≤ 0.01 (**), and p ≤ 0.001 (***). Where applicable, multiple testing correction was performed using the Benjamini-Hochberg false discovery rate (FDR) method. All visualizations were generated using R packages including ggplot2, and significance markers were applied accordingly in plots and tables.

## Results

### CD45 expression and characterization of CTC-like cells in metastatic prostate cancer

**Figure 1:**
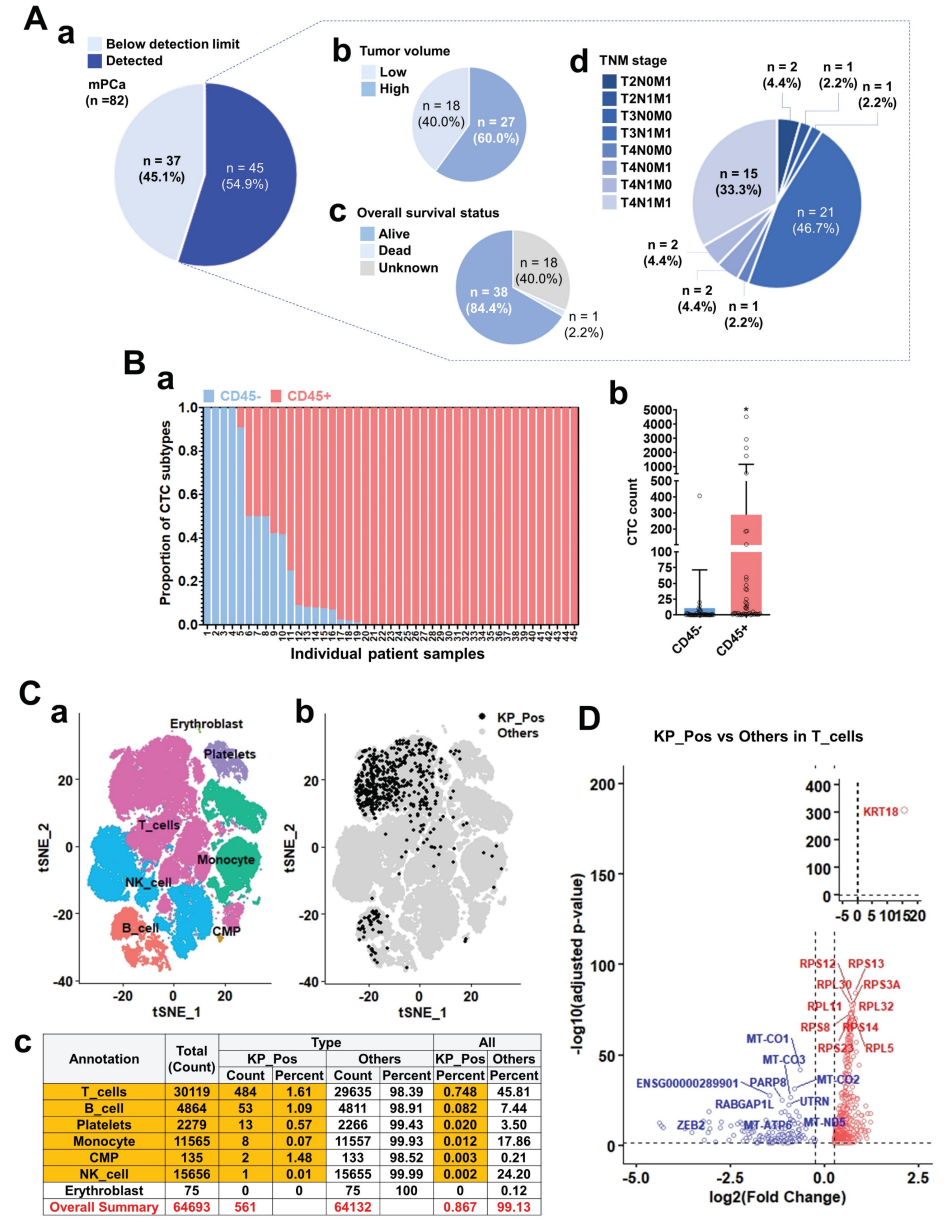
** CD45 expression analysis and characterization of circulating tumor cell (CTC)-like cells in metastatic prostate cancer. (A)** Overview of CTC-like cell detection and associated clinical parameters in metastatic prostate cancer patients (n = 82). (a) Distribution of patients based on CTC-like cell levels above or below the detection limit. (b) Tumor volume classification in patients with detectable CTC-like cells. (c) Overall survival status of patients with detectable CTC-like cells. (d) TNM staging distribution among patients with detectable CTC-like cells. **(B)** Analysis of CD45 expression in cytokeratin-positive CTC-like cells. (a) Patient-wise proportion of CD45⁺ and CD45⁻ populations among cytokeratin-positive CTC-like cells. Cytokeratin positivity was determined using pan-cytokeratin staining. (b) Quantification of cytokeratin-positive CTC-like subsets stratified by CD45 expression status. **(C)** Clustering, mapping, and quantitative summary of peripheral blood mononuclear cells (PBMCs) integrated from seven metastatic prostate cancer (M1 stage) patients. (a) t-SNE (t-distributed Stochastic Neighbor Embedding) plots showing clustering and annotation of PBMCs based on majority-voting classification. Major immune populations including T cells, B cells, NK cells, monocytes, common myeloid progenitors (CMPs), erythroblasts, and platelets were identified. (b) Distribution of double-positive *KRT18⁺* (cytokeratin) and *PTPRC⁺* (CD45) cells, referred to as KP_Pos cells, within the PBMC population. KP_Pos cells (black) and other cells (gray) are visualized across annotated clusters on the t-SNE map. (c) Summary table showing the number and proportion of KP_Pos and other cells within each annotated population. Among 64,693 PBMCs, 561 cells (0.867%) were classified as KP_Pos (highlighted in red), whereas 64,132 cells (99.13%) were classified as others. The overall summary row is highlighted in yellow for emphasis. **(D)** Differentially expressed genes (DEGs) of KP_Pos versus other cells within the T-cell population from PBMCs of seven M1-stage prostate cancer patients. Genes were ranked based on log₂ fold change and adjusted p-values, and significantly upregulated and downregulated genes in KP_Pos T cells are shown in red and blue, respectively. Only the top 10 upregulated and top 10 downregulated genes are labeled to highlight the most significantly altered transcripts.

We investigated CD45 expression in circulating tumor cell (CTC)-like populations to define the cellular identity of CD45⁺ subsets through immunostaining, PBMC clustering, and transcriptomic profiling of metastatic prostate cancer samples. CTC-like cells were detected in the majority of 82 metastatic cases and were stratified by tumor burden, overall survival, and TNM stage (Figure 1A). Their abundance correlated with higher tumor load and advanced disease (Supplementary Figure S1-1A), although CD45 expression itself showed no significant association with clinical parameters (Supplementary Figure S1-1B). Quantification revealed that a substantial portion of cytokeratin⁺ CTC-like cells co-expressed CD45 (Figure 1B-a,b). Immunofluorescence further confirmed CD45⁺/PanCK⁺ dual staining in patient-derived CTC-like cells, with PSA signals often overlapping CD45 (Supplementary Figure S1-1C). Single-cell transcriptomic profiling of PBMCs from seven M1-stage patients identified diverse immune lineages—T cells, B cells, NK cells, monocytes, CMPs, erythroblasts, and platelets (Figure 1C-a). Within these, a rare subset of KRT18⁺ PTPRC⁺ (KP_Pos) cells was detected, primarily among T and B cells (Figure 1C-b), comprising 0.867% (561 of 64,693) of total PBMCs (Figure 1C-c). Annotation using multiple approaches—SingleR (Supplementary Figure S1-2-1), cluster-to-cell-type (S1-2-2), CellTypist (S1-2-3), canonical marker (S1-2-4), and curated subtype markers (S1-2-5)—consistently confirmed immune lineage identities. Independent analyses validated reproducibility across seven PBMC datasets (Supplementary Figures S1-3-1 and S1-3-2-1 to S1-3-2-7). Differential expression analysis comparing KP_Pos and other cells (Figure 1D; Supplementary Table S1-1) revealed upregulation of *KRT18* and multiple ribosomal protein genes (*RPS12*,* RPS13*,* RPL30*,* RPS3A*,* RPL11*,* RPL32*,* RPS8*,* RPS23*,* RPS14*,* RPL5*), indicating enhanced translational activity and partial epithelial-like reprogramming. Downregulated genes included mitochondrial oxidative phosphorylation-related transcripts (*MT-CO1*,* MT-CO2*,* MT-CO3*,* MT-ATP6*,* MT-ND5*) and regulators such as *PARP8*,* RABGAP1L*, *UTRN*, *ZEB2*, consistent with metabolic rewiring and reduced mitochondrial respiration. T cells, accounting for 86.3% of all KP_Pos cells (484/561), were presented as the representative subset for primary DEG analysis, while B cells, monocytes, and platelets showed analogous yet distinct transcriptional changes (Supplementary Figure S1-4; Supplementary Table S1-1). For clarity and consistency, the SingleR cluster-level majority-voting annotation was adopted as the representative framework in the main text. Alternative annotation strategies—CellTypist-, canonical marker-, subtype marker-, and unsupervised cluster-based methods (Supplementary Figures S1-2-1 to S1-2-5)—produced complementary DEG lists (Supplementary Tables S1-2 to S1-6). All DEGs were integrated into a unified DEG pool, ensuring that downstream analyses captured the full transcriptional spectrum for signature development.

### Characterization of CK⁺CD45⁺ cells in primary tumors reveals spatial, morphological, and signaling features linked to CTC-like phenotypes

**Figure 2:**
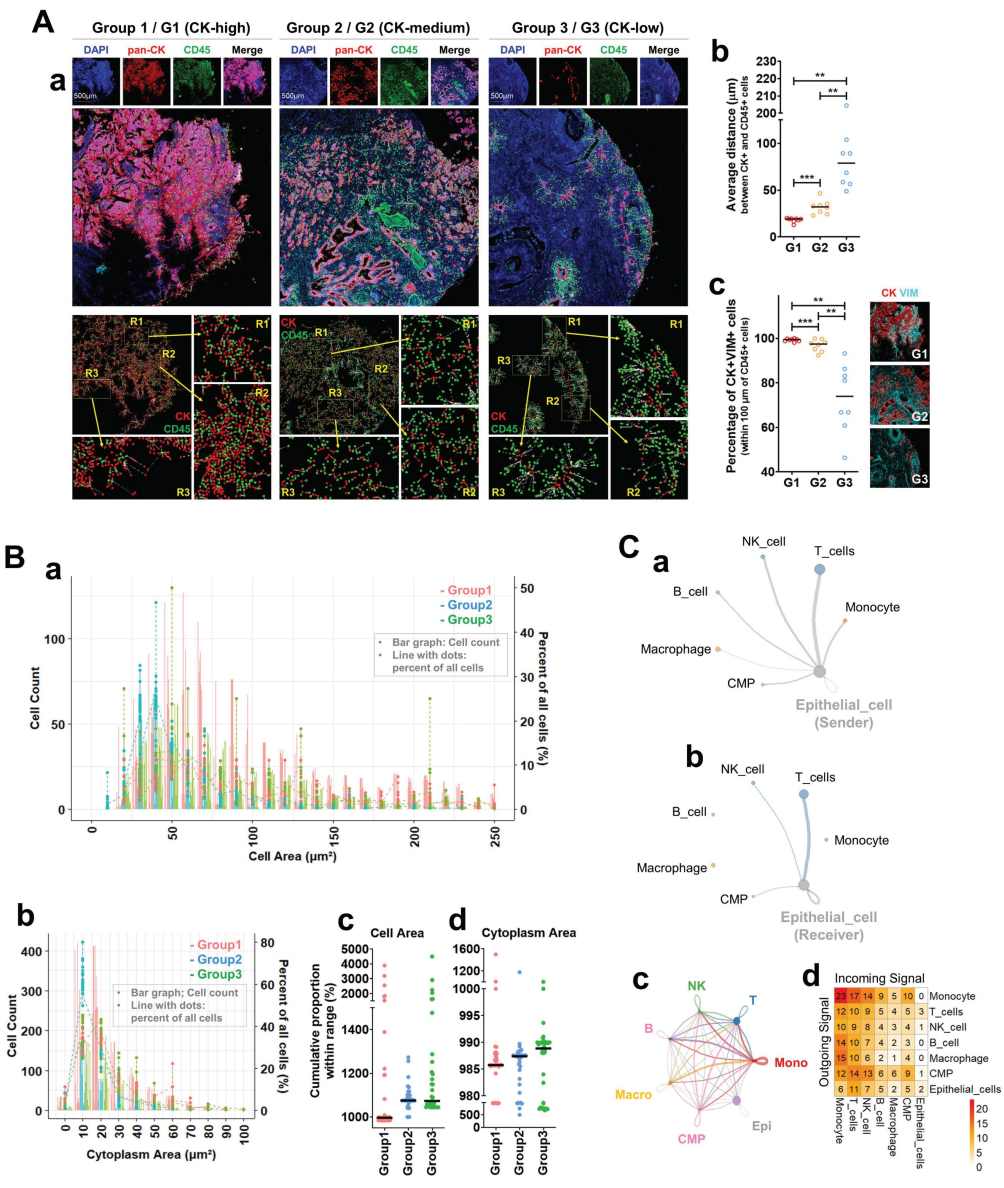
** Spatial and transcriptomic characterization of CK⁺CD45⁺ cells in primary tumors suggests a microenvironmental basis for CTC-like phenotype emergence. (A)** Spatial analysis of CK⁺ and CD45⁺ cells using Imaging Mass Cytometry (IMC). (a) Representative IMC images of metastatic prostate cancer tissues categorized into three groups based on pan-CK expression levels. Top: Individual and merged fluorescence channels showing DAPI (blue), pan-CK (red), and CD45 (green) staining in Group 1 (G1: CK-high), Group 2 (G2: CK-medium), and Group 3 (G3: CK-low). Middle: Composite marker overlays and multiplexed spatial distribution maps at the single-cell level. Bottom: Spatial cell mapping with segmentation showing the distribution of CK⁺ (red) and CD45⁺ (green) cells. Enlarged regions (R1-R3) highlight areas of spatial proximity between these two populations. (b) Quantification of the average distance (µm) between CK⁺ and CD45⁺ cells across the three groups. (c) Proportion of CK⁺VIM⁺ cells—indicative of epithelial-mesenchymal transition (EMT)-like features—among total CK⁺ cells. Statistical significance: *p < 0.05; **p < 0.01; ***p < 0.001. **(B)** Morphometric profiling of CK⁺CD45⁺ double-positive cells. (a) Distribution of total cell area (µm²) of CK⁺CD45⁺ double-positive cells in Group 1 (red), Group 2 (blue), and Group 3 (green). Absolute counts are shown as bar graphs, while relative frequencies (%) are overlaid as line plots with dots. To enhance visibility, values are truncated at 250 µm². The full distribution across the complete range, separated into Count and Percent plots, is provided in Supplementary Figure S2-1-1-1. (b) Distribution of total cytoplasm area within the restricted range, shown separately as Count and Percent plots in Supplementary Figure S2-1-1-2. (c) Cumulative proportions of cells within the defined small-size range for total cell area across the three groups. (d) Cumulative proportions of cells within the defined small-size range for cytoplasmic area across the three groups. Black horizontal lines indicate median values. Quantitative summaries of AUC and global statistics for cell and cytoplasmic area are provided in Supplementary Tables S2-1 to S2-4. **(C)** Intercellular communication analysis centered on epithelial cells using single-cell RNA sequencing. (a, b) Circle plots showing outgoing (a) and incoming (b) signaling interactions of epithelial cells with major immune populations. (c) Circle plot summarizing the overall intercellular communication network among epithelial cells and immune populations, including T cells, NK cells, B cells, macrophages (Macro), monocytes (Mono), and CMPs. (d) Heatmap displaying the overall strength of intercellular communication between epithelial and immune cell populations. All annotations were harmonized with those in Figure 1C. Detailed outgoing and incoming communication profiles for each cell lineage are provided in Supplementary Figures S2-2-1-1 to S2-2-2-2.

To explore the origin and identity of CK⁺CD45⁺ circulating tumor cell (CTC)-like populations observed in peripheral blood (Figure 1), we analyzed metastatic prostate cancer tissues using Imaging Mass Cytometry (IMC) and single-cell RNA sequencing. CK-high tumors exhibited close spatial proximity between CK⁺ and CD45⁺ cells and enriched EMT-like features (Figure 2A). Tumors were stratified into three groups by pan-CK expression: CK-high (G1), CK-medium (G2), and CK-low (G3). IMC imaging revealed dense colocalization of CK⁺ and CD45⁺ cells in G1 tumors (Figure 2A-a), with progressively separated patterns in G2 and G3. Quantitative analysis confirmed significantly shorter CK-CD45 distances in G1 (p < 0.001) and higher frequencies of CK⁺VIM⁺ EMT-like cells (Figure 2A-b,c).

Morphometric analysis of CK⁺CD45⁺ double-positive cells (Figure 2B-a,b) revealed broader size distributions in G1 but a left-shift toward smaller cell and cytoplasmic areas in cumulative proportion plots (Figure 2B-c,d). Summaries of these parameters are provided in Supplementary Tables S2-1 to S2-4, listing cumulative and global size statistics for each CK-defined group. Supplementary Figures S2-1-1-1 and S2-1-1-2 display full distributions of total cell area from Figure 2B-a as count and percent plots (≤ 250 µm²), while Supplementary Figure S2-1-2 shows the corresponding cytoplasmic distributions. These data indicate enrichment of compact, densely distributed immune-epithelial hybrid cells in CK-high tumors. Intercellular communication analysis based on single-cell RNA sequencing of M1-stage tumor biopsies revealed extensive epithelial signaling with multiple immune populations (Figure 2C-a,b). The strongest bidirectional interactions were observed between epithelial cells and T cells, defining a dominant epithelial-T cell signaling axis. The global network (Figure 2C-c) confirmed that epithelial cells were highly integrated with T, NK, B, monocyte, macrophage, and CMP lineages. This network suggests that intense epithelial-T cell cross-talk serves as a central communication hub driving hybrid (CD45⁺/KRT18⁺, KP_Pos) cell formation within the tumor microenvironment. To ensure lineage consistency with Figure 1C, platelets were excluded to avoid signals from circulating components 25, although platelet infiltration into solid tumors has been reported 26. Macrophages were included due to their myeloid lineage continuity with circulating monocytes, which can differentiate into tissue macrophages 27. In the directional interaction heatmap (Figure 2C-d), rows represent signal senders and columns receivers. Monocytes showed the highest outgoing signaling toward epithelial cells, whereas epithelial cells displayed moderate reciprocal signaling to T cells and monocytes. Among all pairs, epithelial-T cell interactions remained the most balanced and sustained. Detailed outgoing and incoming signaling profiles are presented in Supplementary Figures S2-2-1-1 to S2-2-2-2. Supplementary Figure S2-2-1-1 depicts outgoing signals from T, B, NK, monocyte, macrophage, CMP, and epithelial populations, with simplified circle plots in Supplementary Figure S2-2-1-2. Incoming networks for the same populations are shown in Supplementary Figures S2-2-2-1 and S2-2-2-2, highlighting lineage-specific signal reception. These results collectively extend Figure 2C by visualizing directional and quantitative aspects of immune-epithelial communication in metastatic prostate cancer. Finally, quantitative IMC analysis further confirmed CK and CD45 co-expression at the single-cell level (Supplementary Figure S2-3). Scatter plots display CK and CD45 signal intensities from thousands of cells across three CK-defined groups, with three representative cases per group (e.g., G1-1, G1-2, G1-3). Each point represents an individual cell. Together, these IMC and transcriptomic analyses demonstrate spatial, morphological, and signaling evidence supporting the presence and functional relevance of hybrid immune-epithelial (CK⁺CD45⁺) populations in metastatic prostate cancer.

### Distribution and transcriptional features of KP_Pos cells in the metastatic tumor microenvironment

**Figure 3:**
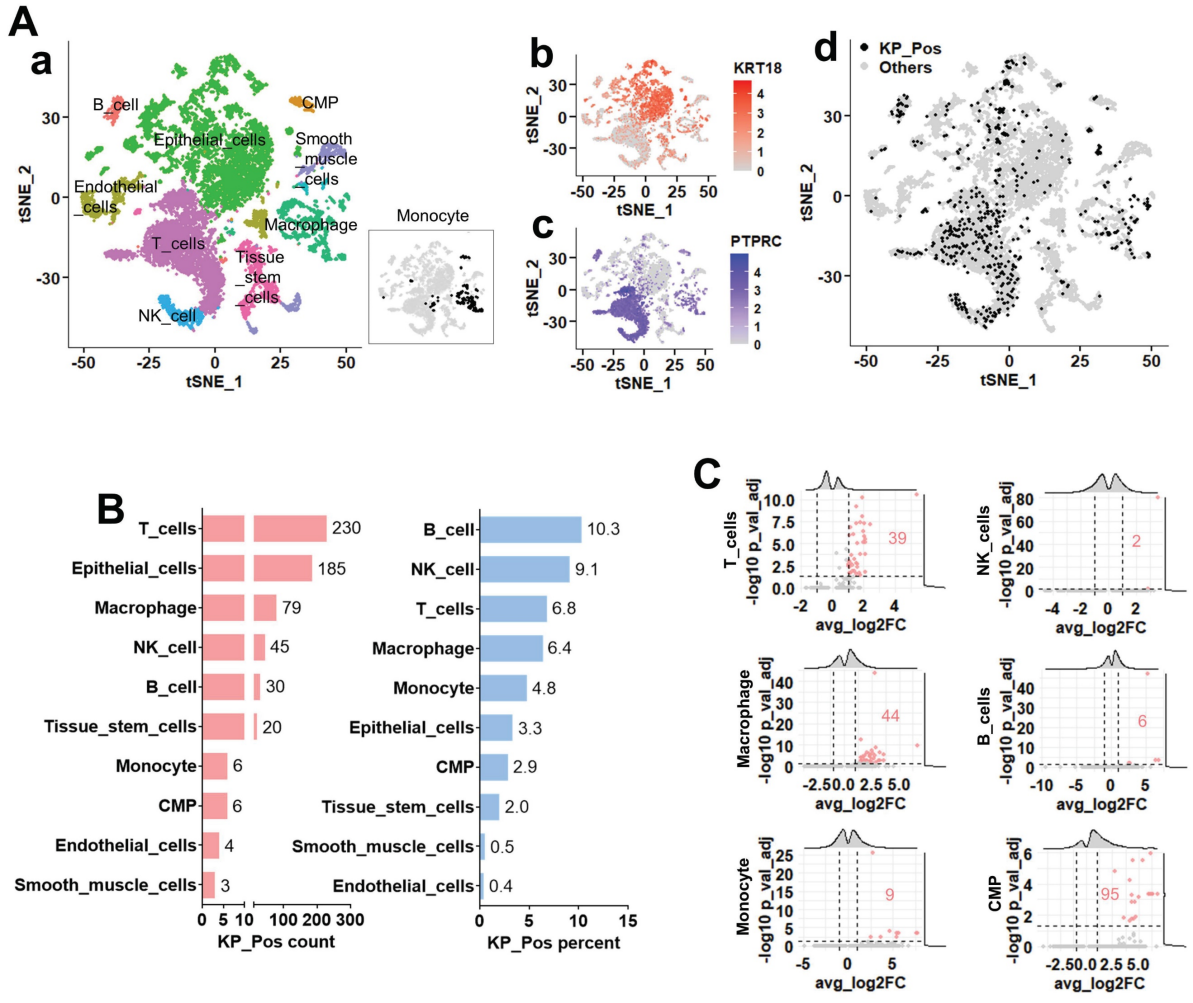
** Single-cell transcriptomic analysis of metastatic prostate cancer reveals the distribution and transcriptional features of KP_Pos (KRT18⁺PTPRC⁺) cells. (A)** Cell clustering and annotation. (a) Cell identities assigned by majority voting across Seurat clusters; since monocytes were not clearly resolved in the clustering, their distribution is separately highlighted in the inset. (b-c) Feature plots showing expression of KRT18 (b) and PTPRC (c). (d) Distribution of KP_Pos cells (KRT18⁺PTPRC⁺) projected onto the t-SNE map. **(B)** Cell type composition of KP_Pos cells. Number and proportion of KP_Pos cells per annotated cell type. **(C)** Differential gene expression in KP_Pos versus others. Volcano plots showing differentially expressed genes between KP_Pos and other cells across six major immune cell types (T cells, NK cells, macrophages, B cells, monocytes, and CMPs). Red numbers indicate the count of significantly upregulated genes (adjusted p < 0.05, log2FC > 1).

To explore the distribution of KP_Pos cells across cell types, we analyzed single-cell transcriptomic profiles from five metastatic prostate cancer biopsy samples, which were entirely independent of the PBMC cases in Figure 1 (no overlap between blood- and tissue-derived datasets). Cluster-level annotation via majority voting identified epithelial, immune, and stromal lineages (Figure 3A-a). Expression maps of KRT18 and PTPRC (Figure 3A-b,c) showed that KP_Pos cells were broadly distributed across the t-SNE embedding (Figure 3A-d). Quantification across annotated lineages revealed that, among the six immune subsets previously detected in PBMCs, KP_Pos cells were most frequent in T cells, macrophages, NK cells, B cells, monocytes, and CMPs (Figure 3B). This distribution pattern was consistently reproduced using six complementary annotation strategies: cell-level annotation (Supplementary Figure S3-1), cluster-level majority voting (S3-2), cluster-to-cell-type mapping (S3-3), CellTypist-based cell subtype annotation (S3-4), marker-based immune annotation (S3-5), and subtype-specific marker-based annotation (S3-6). In all methods, immune populations enriched for KP_Pos cells—particularly T cells, macrophages, NK cells, B cells, monocytes, and CMPs—were highlighted in orange in the accompanying summary tables. To define the molecular characteristics of KP_Pos cells, differential gene expression analysis was performed between KP_Pos and Other cells within the six major immune lineages. Volcano plots revealed distinct sets of significantly upregulated genes in KP_Pos cells across lineages, with the highest numbers observed in CMPs, macrophages, and T cells (Figure 3C). Complete gene lists for all annotation strategies are provided in Supplementary Tables S3-1 to S3-6.

### Single-cell transcriptomic profiling identifies metastasis-specific cellular and molecular features of KP_Pos cells

**Figure 4:**
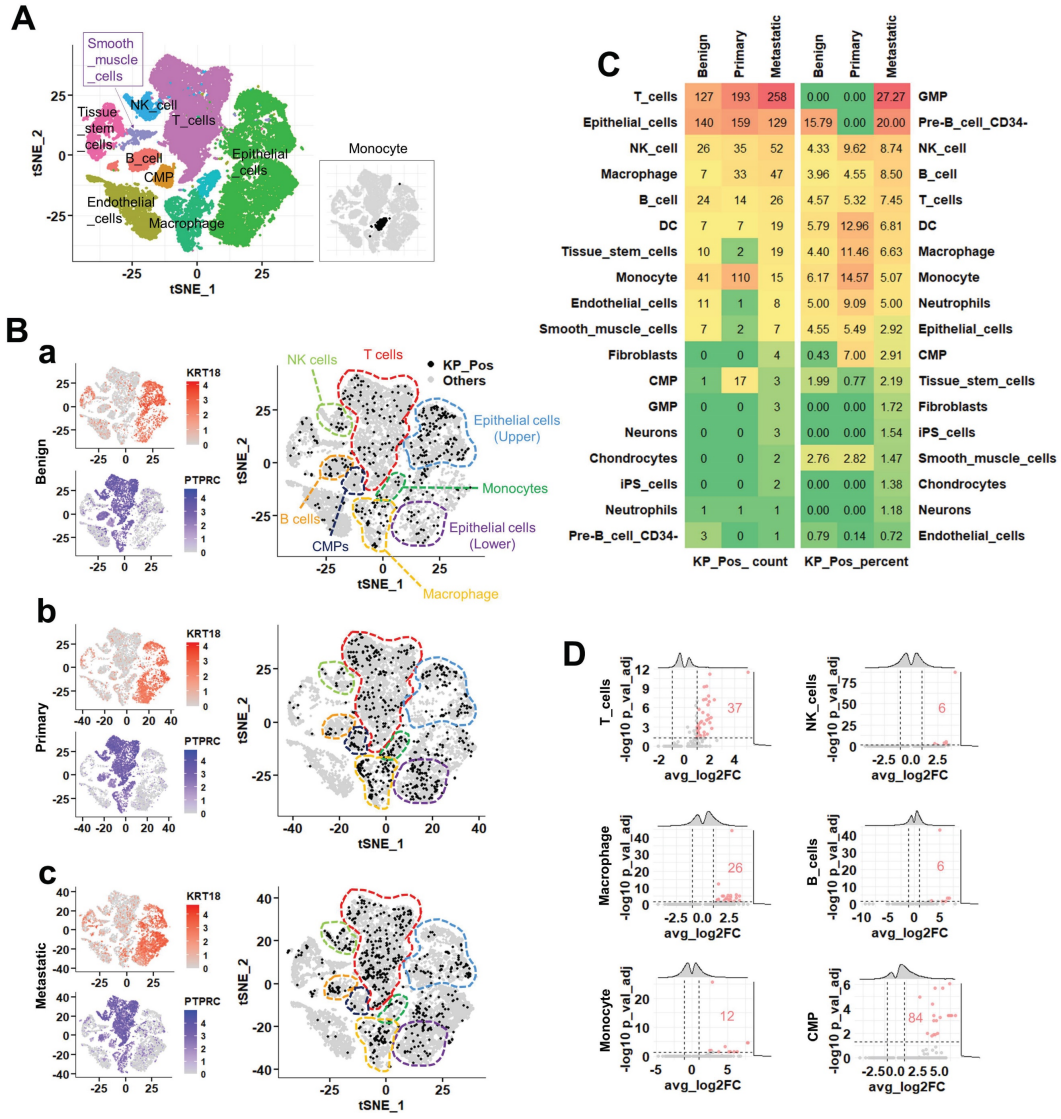
** Integrated single-cell transcriptomic analysis of KP_Pos populations across benign, primary, and metastatic prostate cancer. (A)** Clustering of integrated single-cell data. Annotation based on majority voting across Seurat-defined clusters. Since monocytes were not clearly resolved in the clustering, their distribution is separately highlighted in the inset (rectangular box) of the t-SNE map. **(B)** Distribution of KP_Pos and others in t-SNE space and marker gene expression. Left: Expression levels of KRT18 and PTPRC across benign (top), primary (middle), and metastatic (bottom) samples. Right: Distribution of KP_Pos (KRT18⁺PTPRC⁺) and other cells in t-SNE space for each disease stage (a, benign; b, primary; c, metastatic). Dashed boundaries delineate major cell lineages: T cells (red), NK cells (light green), B cells (orange), macrophages (yellow), monocytes (green), CMPs (navy), and epithelial cells (sky blue and purple). KP_Pos cells (black dots) were broadly distributed across multiple immune and epithelial lineages. **(C)** Cell type composition of KP_Pos cells. Heatmap showing the number (left) and percentage (right) of KP_Pos cells across cell types in benign, primary, and metastatic samples. Color intensity reflects values from low (green) to high (red). **(D)** Metastasis-specific differentially expressed genes in KP_Pos cells. Volcano plots displaying DEGs in KP_Pos cells from metastatic samples compared to benign and primary samples within six major immune cell types: T cells, NK cells, B cells, Macrophages, Monocytes, and CMPs. Significantly upregulated genes are marked in red (adjusted p < 0.05 and |log₂FC| > 0.25). DEG counts are annotated within each plot.

To identify metastasis-specific alterations in the composition and gene expression of KP_Pos (KRT18⁺PTPRC⁺) cells, we conducted an integrated single-cell transcriptomic analysis of benign, primary, and metastatic prostate cancer tissues. Seurat-based majority voting confirmed the presence of KP_Pos cells across all stages (Figure 4A). In t-SNE projections (Figure 4B-a-c), KP_Pos cells showed lineage- and stage-dependent distribution patterns: T cells (red outline) exhibited a progressive increase from benign to metastatic states; epithelial cells (sky blue, upper cluster) decreased gradually, while another epithelial subset (purple, lower cluster) displayed a biphasic pattern (primary > metastatic > benign). Monocytes (green outline) demonstrated a marked enrichment in primary tumors, whereas CMPs, B cells, macrophages, and NK cells showed minimal stage-specific variation. Quantitative comparison revealed a lineage shift in KP_Pos composition across disease stages, with macrophage-derived KP_Pos populations predominating in metastatic tumors and CMP-associated KP_Pos cells enriched mainly in primary tissues (Figure 4C). Results from five complementary reference-based cell-level annotation methods, consistent with the Seurat-based majority-voting annotation, are summarized in Supplementary Tables S4-1-1 to S4-1-6 (table only, without figure presentation due to overlap with Figure 3). To further characterize KP_Pos heterogeneity in metastasis, we analyzed subtype distributions within three major compartments. In epithelial cells, KP_Pos cells were enriched in the Epithelial_EMT subtype (Supplementary Figure S4-1). Within T cells, they were predominantly associated with T_Memory and T_Exhausted phenotypes (Supplementary Figure S4-2). Among monocytes, KP_Pos cells were less abundant in metastasis but enriched in the Mono_NonClassical subtype during earlier stages (Supplementary Figure S4-3). Differential gene expression analysis comparing metastatic, primary, and benign KP_Pos cells across six immune lineages—T cells, NK cells, B cells, macrophages, monocytes, and CMPs—revealed lineage-specific transcriptional changes (Figure 4D). Distinct upregulated gene sets were most prominent in T cells and CMPs, indicating activation of metastatic programs in these populations (Supplementary Tables S4-2-1 to S4-2-6).

### Construction and evaluation of scRNA-seq-based gene signatures for M0/M1 classification

**Figure 5:**
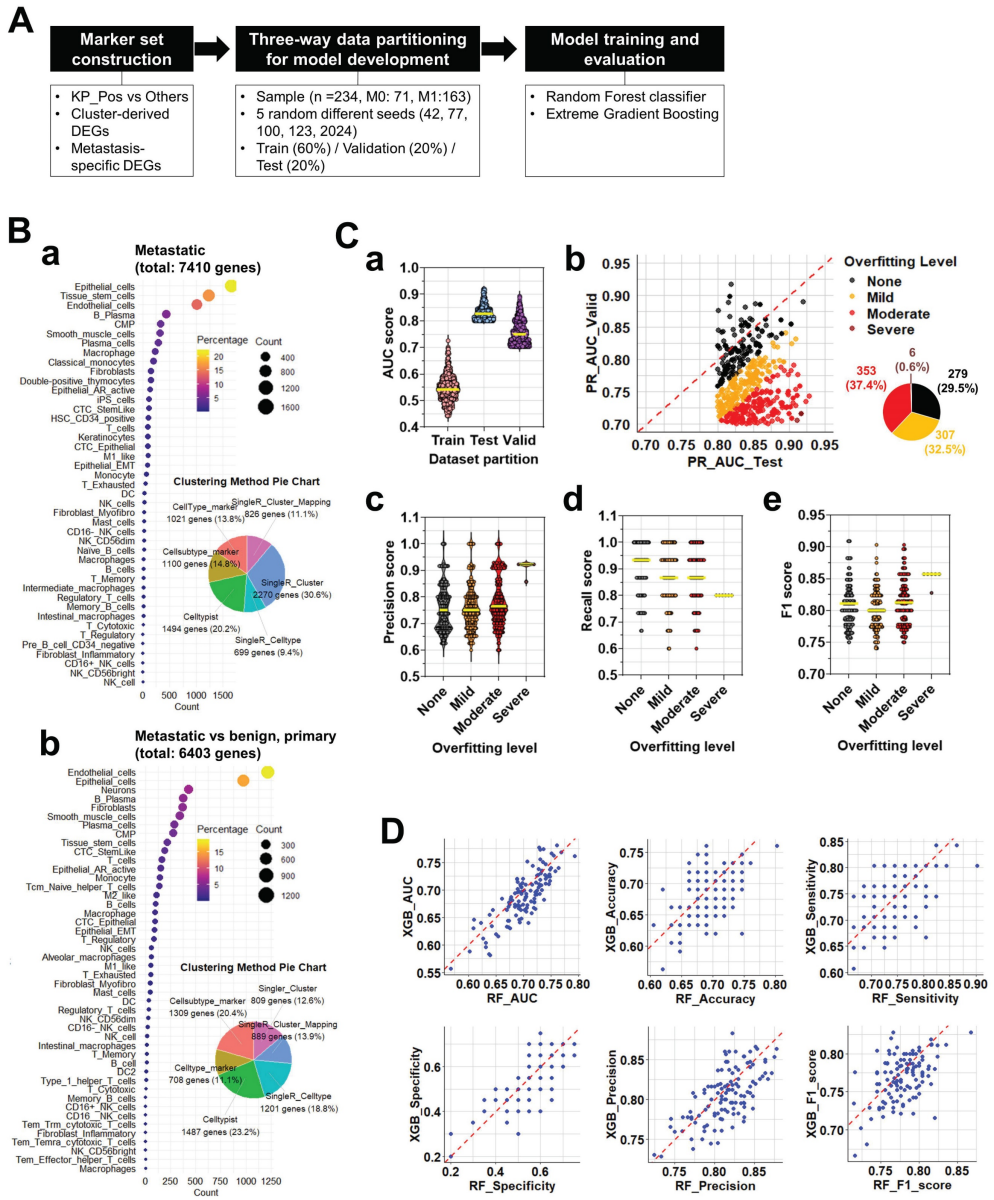
** Construction and evaluation of predictive signatures for M0/M1 classification based on differentially expressed genes. (A)** Workflow for signature evaluation. Step 1: Marker set construction using cluster-derived DEGs and metastasis-specific DEGs identified from cell-level annotation strategies. Step 2: Three-way data partitioning for model development using bulk RNA-seq data combined with clinical information. Five independent random seeds were applied for reproducibility analysis. Step 3: Signature validation through model training and evaluation using Random Forest (RF) and Extreme Gradient Boosting (XGB) algorithms. **(B)** Composition of the marker Pool. (a) Cell type and clustering method-dependent distribution of cluster-derived DEGs. The left panel shows a bubble plot summarizing the number of DEGs per cell type across annotation methods. The right pie chart displays proportional contributions from each method (SingleR, CellTypist, etc.). (b) Cell type and clustering method-dependent distribution of metastasis-specific DEGs. The left panel shows a bubble plot summarizing DEGs derived from metastasis-specific comparisons, while the right pie chart shows contributions from each method. **(C)** Three-way data partitioning analysis for model development. (a) AUC scores for training, validation, and test datasets. (b) Overfitting level evaluation of signatures, based on the difference between PR_AUC in validation and test sets. Scatter plot with pie chart summarizes the proportion of signatures categorized as none, mild, moderate, or severe overfitting. (c) Precision scores of signatures according to overfitting level. (d) Recall scores of signatures according to overfitting level. (e) F1 scores of signatures according to overfitting level. Yellow horizontal lines indicate the average score within each group. **(D)** Comparative performance of RF and XGB models using signatures from the none overfitting group. Eight scatter plots display the correlation between RF and XGB models in terms of AUC, accuracy, sensitivity, specificity, precision, and F1 score for signatures classified as none in overfitting level evaluation.

To identify gene signatures predictive of metastatic status (M0 vs. M1), we implemented a multi-step workflow encompassing marker selection, model development, and performance evaluation (Figure 5A). Differentially expressed genes (DEGs) were collected from two major sources: (i) cluster-derived DEGs obtained from diverse annotation methods (SingleR at cluster- and cell type-levels, cluster-level majority voting, cluster-to-cell-type mapping, CellTypist, and marker-based references) and (ii) metastasis-specific DEGs identified by comparing epithelial cells from metastatic, benign, and primary prostate tissues. In total, 7,488 cluster-derived and 6,408 metastasis-specific DEGs were compiled, encompassing epithelial, immune, and stromal populations (Figure 5B-a,b). All DEGs—including those distinguishing KP_Pos versus other cells across immune lineages—were pooled to construct candidate marker sets for M0/M1 classification. Each marker set was evaluated through three-way data partitioning (training, validation, and test sets), and classification performance was assessed primarily by the precision-recall area under the curve (PR-AUC) to correct for class imbalance. Marker sets achieving PR-AUC ≥ 0.7 in the test dataset were retained as high-performing, yielding 945 predictive signatures. This threshold was selected to ensure clinical relevance and avoid overfitting 28. Random Forest (RF) and Extreme Gradient Boosting (XGB) models were then applied in parallel to the 945 signatures. Cross-model evaluation compared AUC, PR-AUC, accuracy, sensitivity, specificity, precision, and F1-score, leading to the identification of 119 consistently robust signatures with minimal overfitting. Pie charts illustrate the proportional contribution of each annotation method to the final marker pool. To validate stability, each signature was trained and tested using five random seeds (Figure 5C-a). AUC distributions across partitions confirmed consistent model behavior. Overfitting was assessed by PR_AUC differences between validation and test sets, classifying signatures into four categories—None, Mild, Moderate, or Severe (Figure 5C-b). Among the 945 candidates, 29.5% showed no overfitting, 32.5% mild, 37.4% moderate, and only 0.6% severe. Performance metrics, including precision (Figure 5C-c), recall (Figure 5C-d), and F1 score (Figure 5C-e), declined progressively with increasing overfitting severity, as indicated by lower medians and broader distributions. Yellow bars denote mean performance within each category. To compare algorithmic consistency, we analyzed overfitting-free (None) signatures across both RF and XGB models (Figure 5D). All six performance metrics—AUC, accuracy, sensitivity, specificity, precision, and F1 score—showed strong inter-model correlation, confirming robust, model-independent predictive capacity. Performance variations among all signatures were further visualized using stratified boxplots and heatmaps (Supplementary Figure S5A-B). Classification performance declined modestly from None to Severe groups, with AUC and F1 scores showing the steepest reductions, while specificity and precision remained relatively stable (Supplementary Figure S5A-a, S5B-a). Heatmaps of normalized performance metrics highlighted clusters of top-performing signatures, and the top 20 signatures for RF and XGB were ranked and visualized (Supplementary Figure S5A-b, S5B-b). Comprehensive datasets are provided in Supplementary Tables S5-1 to S5-3, including the full list of predictive gene signatures with gene composition and partitioning results across random seeds (S5-1), detailed RF/XGB performance metrics (S5-2), and overfitting classification for each signature (S5-3).

### Composite scoring identifies robust gene signatures predictive of metastatic prostate cancer and reveals clinical correlations

**Figure 6:**
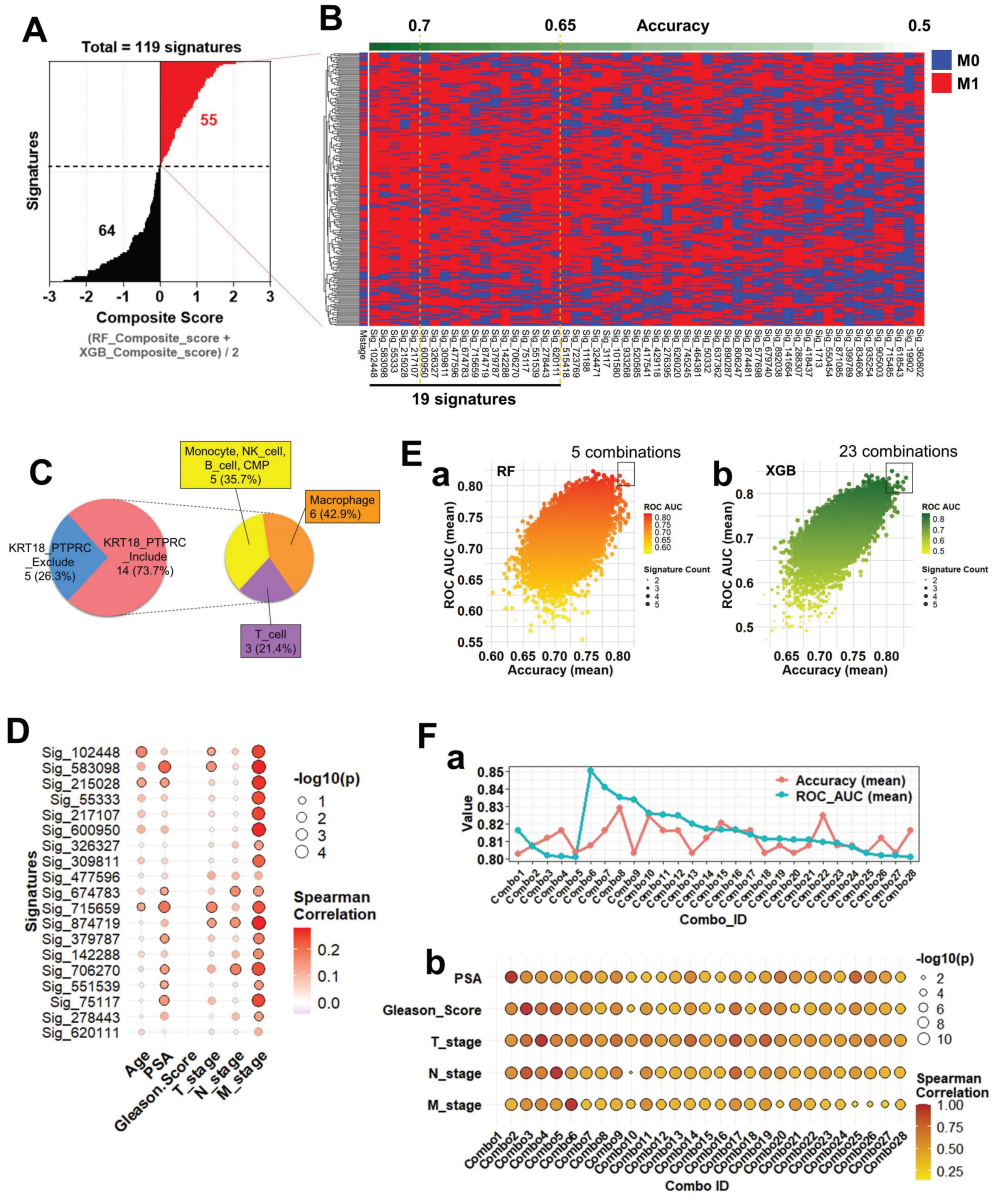
** Composite score-based signature evaluation and clinical correlation analysis. (A)** Distribution of composite scores across 119 gene signatures predictive of metastatic status (M0 vs. M1). Composite scores were calculated as the average of RF and XGBoost-derived scores. Among them, 55 signatures with positive composite scores (red) and 64 with negative scores (black) were identified. **(B)** Heatmap illustrating M-stage prediction (M0: blue, M1: red) across bulk RNA-seq samples using the 119 signatures, sorted by composite score. The top-performing signatures (accuracy ≥ 0.7, n = 5; accuracy ≥ 0.65, n = 19) are highlighted. **(C)** Cell-of-origin analysis for the 19 signatures with accuracy ≥ 0.65. Based on the presence of KRT18 and/or PTPRC, signatures were grouped into 'Include' or 'Exclude'. The 'Include' group was further classified into three cell-type categories: (1) monocyte, NK cell, B cell, and CMP (35.7%), (2) macrophage (42.9%), and (3) T cell (21.4%). **(D)** Spearman correlation between individual signature scores (top 19) and clinical variables (Age, PSA, Gleason Score, T_stage, N_stage, M_stage). Circle color represents correlation strength, size reflects -log₁₀(p-value), and circles with black outlines indicate statistical significance (p < 0.05). **(E)** Prediction performance of multi-signature combinations assessed using (a) RF and (b) XGB models. Here, “combinations” refer to all possible sets of 2 to 5 signatures drawn from the top 19 signatures identified in panel B. Each dot corresponds to a unique combination, with color denoting ROC AUC and size indicating the number of included signatures. **(F)** Evaluation of selected signature combinations, where each combination was derived from the top 19 signatures (2-5 signatures per combination). a. Line plots showing average accuracy (red) and average ROC AUC (blue) for each combination across the test dataset. b. Correlation analysis between combined signature scores and clinical parameters. Dot size indicates -log₁₀(p-value), color represents correlation coefficient, and black outlines highlight significant correlations (p < 0.05).

To systematically evaluate gene signatures predictive of metastatic status (M0 vs. M1), we analyzed 119 candidate gene signatures constructed from scRNA-seq-derived DEGs. For each signature, a composite score was computed as the averaged performance from RF and XGB models. Based on these scores, 55 positive and 64 negative signatures were identified (Figure 6A; Supplementary Table S6-1-1). Applying all 119 signatures to bulk RNA-seq data, we visualized M-stage classification outcomes via heatmap (Figure 6B). Signatures were ranked by composite score, with individual predictions shown per sample (M0: blue; M1: red). Among them, 19 signatures achieved mean accuracy ≥0.65, including five ≥0.7 (Supplementary Table S6-1-2), indicating strong predictive potential. Subsequently, all possible combinations (Combos) of 2-5 gene signatures from these top 19 were tested to assess whether integration improves M-stage prediction relative to single-signature models. The cellular origins of the 19 top-performing signatures were then analyzed. Based on inclusion of epithelial (KRT18) and immune (PTPRC) markers, signatures were classified as Include or Exclude.

The majority (73.7%) belonged to Include, subdivided into macrophage (42.9%), monocyte/NK/B cell/CMP (35.7%), and T cell (21.4%) subgroups (Figure 6C), indicating meaningful contributions from both immune- and epithelial-derived genes. Prediction heterogeneity across the full 945 signatures was visualized using heatmaps (Supplementary Figure S6-1). Considerable variability was observed, yet lineage-based grouping revealed that monocyte-, NK-, B cell-, and CMP-derived Include signatures exhibited classification patterns comparable to macrophage- and T cell-derived ones, underscoring their robustness. To assess clinical relevance, we performed Spearman correlation analysis between signature scores and clinical parameters (Age, PSA, Gleason Score, T_stage, N_stage, and M_stage). Most predictive signatures correlated significantly with M_stage (Figure 6D), and particularly Sig_583098 and Sig_715659 also showed associations with PSA and T_stage, supporting their clinical utility as metastasis-related biomarkers. Next, we tested whether combining multiple signatures enhances prediction accuracy. Ensemble models using RF (Figure 6E-a) and XGB (Figure 6E-b) evaluated diverse signature combinations. Each dot in the scatterplots represents one unique combination, with dot size indicating the number of signatures and color denoting ROC AUC. Several combinations achieved ROC AUC > 0.8, demonstrating the advantage of multi-signature integration (Supplementary Tables S6-2-1 and S6-2-2). Selected combinations were further validated for accuracy and clinical correlation. Multiple combinations maintained high test-set accuracy and ROC AUC (Figure 6F-a). Correlation analyses (Figure 6F-b) revealed significant associations with Gleason Score, T_stage, and M_stage, confirming both robustness and clinical interpretability. Interestingly, Combo 1, despite strong M-stage prediction, showed no correlation with clinical parameters, suggesting that it captures metastasis-linked transcriptomic features independent of conventional variables.

## Discussion

Metastasis remains the leading cause of mortality among men with prostate cancer (PCa), and reliable prediction of metastatic potential remains a major unmet clinical need. In this study, we investigated the transcriptomic and spatial characteristics of hybrid circulating tumor cell (CTC)-like cells co-expressing epithelial (KRT18) and immune (CD45/PTPRC) markers, termed KP_Pos, to elucidate their origin and clinical significance. Through integrated Imaging Mass Cytometry (IMC), single-cell RNA sequencing (scRNA-seq), and bulk RNA-seq-based modeling, we identified lineage-specific transcriptomic programs and gene signatures associated with metastasis and demonstrated their predictive power in stratifying patients by metastatic status.

Spatial IMC analysis of metastatic prostate tumor microenvironments revealed close proximity and frequent interaction between CK⁺ epithelial and CD45⁺ immune cells, coinciding with the emergence of CK⁺CD45⁺ hybrid phenotypes. Although such cells have been linked to fusion-related enlargement 9, 29, our morphometric profiling revealed an opposite pattern: CK⁺CD45⁺ double-positive cells in CK-high tumors (Group 1) were smaller yet more abundant than in other groups, with size distributions shifted toward compact morphologies (Figure 2B-c,d). These compact phenotypes, observed across multiple lineage markers (CD14, CD16, CD3, CD8A, Granzyme, Perforin), likely represent metabolically active states rather than quiescence, consistent with small but functionally potent CD45RO⁺ memory T cells and CD16⁺ NK cells 30, 31. IMC quantification confirmed enrichment of activation and checkpoint molecules (CD25, HLA-DR, PD-1) in these compact hybrids, aligning with evidence that morphologically small circulating cells increase in advanced disease and predict poor prognosis 32. Collectively, these findings suggest that KP_Pos cells are compact, active immune-epithelial hybrids engaged in tumor-immune communication rather than simple fusion products. Consistently, CellChat analysis (Figure 2C) demonstrated that epithelial cells acted as both senders and receivers of intercellular signals with monocytes and T cells, underscoring a bidirectional epithelial-immune signaling network in KP_Pos emergence.

In PBMC scRNA-seq from M1-stage metastatic PCa patients, KP_Pos T cells exhibited a distinct expression profile marked by *KRT18* and multiple ribosomal genes (*RPS12*,* RPS13*,* RPL30*,* RPS3A*,* RPL11*,* RPL32*,* RPS8*,* RPS23*,* RPS14*,* RPL5*), indicating partial epithelial-like reprogramming possibly driven by tumor-derived factors or extracellular vesicle-mediated transcript transfer 33. Enhanced ribosomal expression implies increased translational capacity and adaptation to circulatory stress 34, whereas downregulation of mitochondrial oxidative phosphorylation (*OXPHOS*) genes (*MT-CO1*,* MT-CO2*,* MT-CO3*,* MT-ATP6*,* MT-ND5*) and regulators (*PARP8*,* RABGAP1L*,* UTRN*,* ZEB2*) indicates metabolic rewiring toward glycolytic states linked to T-cell exhaustion 35, 36. These data suggest that KP_Pos T cells constitute metabolically altered, transcriptionally hybrid subsets imprinted by tumor-derived molecular signals 37. Similar epithelial-like gene patterns appeared across other immune lineages: B cells co-upregulated *KRT18*,* RPS12*,* RPL9*,* RPS19*,* RPL8*,* RPL12*,* RPS18*,* RPS17*, and stress-related genes *RASSF7* and *PLIN3*, suggesting cytoskeletal and lipid metabolic adaptation 38, 39; monocytes induced *KRT18*, *PEF1-AS1*, and *SLCO1B3*, indicating xenobiotic responsiveness 40, 41; and platelets showed unexpected *KRT18*, *S1PR2*, *CDK5R1*, *TET1*, and *PMS2/PMS2P3* upregulation, reflecting epithelial transcript uptake or intercellular RNA transfer 42. These convergent profiles across immune subsets support a systemic tumor-immune molecular exchange, generating shared hybrid transcriptional programs (Figures 1, 3-4; Supplementary Figures S1-S4; Supplementary Tables S3-1 to S3-6).

Within metastatic tumor scRNA-seq datasets, multiple annotation approaches (SingleR, CellTypist, marker-based mapping) confirmed the presence of KP_Pos cells across immune (macrophages, monocytes, T cells) and epithelial compartments. Transcriptomic comparisons revealed enrichment of immune-response, antigen-presentation, and epithelial-mesenchymal transition (EMT) pathways, suggesting biological—not artifactual—origins. Dual validation at protein and transcript levels (IMC and scRNA-seq) confirmed KRT18/CD45 co-expression (Supplementary Figures S1-1C, S2-3). The distribution of KP_Pos cells differed by context: circulating PBMC hybrids localized mainly within T and B cells, while tissue-resident KP_Pos cells (Figures 3-4) included NK, macrophage, epithelial, and stromal subsets, reflecting microenvironmental pressures that drive hybrid diversity.

Functional enrichment analyses (Supplementary Figures S6-2, S6-3-1-S6-3-9) revealed consistent enrichment of immune, antigen-presentation, and EMT pathways, consistent with evidence that the TME promotes stemness and therapy resistance 43, that EMT activation correlates with immune evasion 44, 45, and that EMT-related transcriptional programs predict poor outcomes 46. Reports that transcriptionally primed cells can drive lymph node-independent metastasis 47 further support KP_Pos cells as metastasis-competent intermediates.

From these data, we derived 945 lineage-specific gene signatures and assessed metastatic classification performance using Random Forest (RF) and Extreme Gradient Boosting (XGB) models. Several individual signatures achieved ≥0.7 accuracy, while combinations of top-performing signatures reached ≥0.8, indicating additive predictive value (Figures 5-6). The use of multiple clustering and annotation methods was essential to capture hybrid diversity and prevent bias toward dominant lineages (Supplementary Figures S5A-B, S6-1; Supplementary Tables S5-1 to S5-3, S6-1-1, S6-1-2, S6-2-1, S6-2-2; Reference 26). These predictive signatures bridge molecular characteristics of primary tumors and CTC-like hybrids, supporting the concept that primary tumor transcriptional states can inform metastatic potential. The existence of CD45⁺/KRT⁺ hybrid CTCs in advanced prostate and breast cancers 10, 11 reinforces this biological continuum linking tumor-immune interaction and systemic dissemination.

Several limitations must be acknowledged. All analyses were based on a single internal cohort. External validation using TCGA_PRAD and SU2C_PRAD datasets was limited by differences in sequencing platforms and gene coverage (TCGA_PRAD: 20,531 genes; SU2C_PRAD: 19,293 genes; our dataset: 36,553 genes), which precluded complete model transfer and prevented direct testing of our predictive signatures without compromising integrity (Figures 5-6; Supplementary Tables S5-1 to S5-3, S6-1-1, S6-1-2, S6-2-1, S6-2-2). Moreover, scRNA-seq primarily detects upregulated genes due to dropout effects 48, yet these transcripts remain the most reliable for signature generation 49-51. Discrepancies between single-cell and bulk RNA-seq data reflect inherent differences in resolution and have been similarly reported in other studies 51-53. Although panCK⁺/CD45⁺ CTC-like cells were detected in the blood of 45 metastatic prostate cancer patients, standardized enrichment protocols and independent validation of their prognostic utility beyond PSA and Gleason score will be essential in future prospective and longitudinal studies.

## Conclusion

Our study establishes the existence and clinical relevance of CD45⁺CK18⁺ (PTPRC⁺KRT18⁺) hybrid CTC-like cells in metastatic prostate cancer. By linking their emergence to epithelial-immune signaling, metabolic remodeling, and EMT programs, we identify predictive gene signatures capable of distinguishing metastatic status with high accuracy. These findings provide a framework for non-invasive biomarker development, illuminate the biology of immune-epithelial plasticity, and suggest new therapeutic opportunities targeting hybrid-cell formation. Nevertheless, as this study was based on cross-sectional transcriptomic data without longitudinal survival analysis, the causal and prognostic roles of KP_Pos cells remain to be clarified.

## Supplementary Material

Supplementary figures and tables.

## Abbreviations

AUC: Area under the curve
CD45: Cluster of Differentiation 45, also known as Leukocyte Common Antigen (LCA)
CK: cytokeratin
CMP: Common myeloid progenitor
CTC: Circulating tumor cell
DEG: Differentially expressed gene
EMT: Epithelial-mesenchymal transition
IMC: Imaging Mass Cytometry
KP_Pos: KRT18⁺PTPRC⁺ (CD45⁺KRT18⁺)
KRT18: Cytokeratin 18
M0: No distant metastasis detected
M1: Distant metastasis present
Macro: macrophage
Mono: monocyte
mPCa: metastatic prostate cancer
MT-ATP6: Mitochondrially Encoded ATP Synthase Membrane Subunit 6
MT-CO1: Mitochondrially Encoded Cytochrome c Oxidase I
MT-CO2: Mitochondrially Encoded Cytochrome c Oxidase II
MT-CO3: Mitochondrially Encoded Cytochrome c Oxidase III
MT-ND5: Mitochondrially Encoded NADH:Ubiquinone Oxidoreductase Core Subunit 5
NK: Natural killer
PanCK: Pan-Cytokeratin
PARP8: Poly(ADP-Ribose) Polymerase Family Member 8
PBMC: Peripheral blood mononuclear cell
PCa: Prostate cancer
PR-AUC: Precision-recall area under the curve
PSA: Prostate-specific antigen
PSMA: Prostate-specific membrane antigen
PTPRC: Protein tyrosine phosphatase receptor type C, a gene encoding CD45
RABGAP1L: RAB GTPase Activating Protein 1 Like
RF: random forest
ROC AUC: Receiver operating characteristic area under the curve
RPL11: Ribosomal Protein L11
RPL30: Ribosomal Protein L30
RPL32: Ribosomal Protein L32
RPL5: Ribosomal Protein L5
RPS12: Ribosomal Protein S12
RPS13: Ribosomal Protein S13
RPS14: Ribosomal Protein S14
RPS23: Ribosomal Protein S23
RPS3A: Ribosomal Protein S3A
RPS8: Ribosomal Protein S8
TME: Tumor microenvironment
TNM stage: Tumor-Node-Metastasis stage
t-SNE: t-distributed Stochastic Neighbor Embedding
UTRN: Utrophin
Vim: vimentin
XGB: Extreme Gradient Boosting
ZEB2: Zinc Finger E-box Binding Homeobox 2
