# Supplementary material for: CD45⁺ hybrid circulating cells may reflect tumor-immune interactions and serve as transcriptomic indicators of metastatic potential in prostate cancer
Source: Theranostics. 2026 Jan 1;16(3):1594–612. doi: 10.7150/thno.122226 (PMC12679700; doi:10.7150/thno.122226)
Supplement: Supplementary file 1 — Supplementary figures and tables. [file thnov16p1594s1.zip › Supplementary_Data.pdf]

Supplementary Figures with Legends

A

Group	Age	PSA	TNM stage			CTC-like cell (number)		
						Total	CD45-	CD45+
M0	60	570.2	T4	N1	M0	1	0	1
	74	31.4	T4	N1	M0	1	0	1
	60	233.69	T4	N0	M0	1	0	1
	66	32.16	T3	N0	M0	1	1	0
N0M1	81	4865.7	T2	N0	M1	3	0	3
	82	1.71	T2	N0	M1	2	0	2
	77	38.1	T3	N0	M1	1	0	1
	69	77.05	T3	N0	M1	4	1	3
	74	418.8	T3	N0	M1	2	1	1
	64	146	T4	N0	M1	5	0	5
	63	150	T4	N0	M1	2	0	2
T3N1M1	56	240	T2	N1	M1	2335	0	2335
	66	2.7	T3	N1	M1	56	0	56
	65	78.2	T3	N1	M1	25	0	25
	79	2209.3	T3	N1	M1	20	0	20
	87	254	T3	N1	M1	18	0	18
	72	493.5	T3	N1	M1	3	0	3
	67	13631.9	T3	N1	M1	2	0	2
	71	784.52	T3	N1	M1	2	0	2
	73	56	T3	N1	M1	1	0	1
	62	67.3	T3	N1	M1	1	0	1
	76		T3	N1	M1	555	7	548
	79	13.2	T3	N1	M1	13	1	12
	60	16.6	T3	N1	M1	51	4	47
	75	138	T3	N1	M1	11	1	10
	64	10.94	T3	N1	M1	19	8	11
	56	82.4	T3	N1	M1	6	3	3
	57	106.3	T3	N1	M1	2	1	1
	72	75.23	T3	N1	M1	22	20	2
	58	105	T3	N1	M1	1	1	0
T4N1M1	60	270.11	T4	N1	M1	2934	0	2934
	79	108.28	T4	N1	M1	1767	0	1767
	73	261.3	T4	N1	M1	187	0	187
	81	480	T4	N1	M1	185	0	185
	65	546	T4	N1	M1	60	0	60
	67	72.95	T4	N1	M1	1	0	1
	69	>100	T4	N1	M1	1	0	1
	73	5.26	T4	N1	M1	1	0	1
	68	613.4	T4	N1	M1	105	2	103
	70	48.25	T4	N1	M1	42	1	41
	62	279.1	T4	N1	M1	43	3	40
	72	9.1	T4	N1	M1	4943	407	4536
	51	545	T4	N1	M1	24	10	14
	65	100	T4	N1	M1	6	6	0
	73	485.69	T4	N1	M1	14	14	0

B

Correlation coefficient (upper triangle); p-value (lower triangle)

	Age	PSA	TNM stage T	TNM stage N	TNM stage M	CD45-	CD45+
Age		0.071	-0.176	-0.121	0.146	0.054	-0.051
PSA	0.648		-0.196	-0.002	0.065	-0.052	-0.075
TNM stage T	0.248	0.202		0.22	-0.192	0.157	0.11
TNM stage N	0.429	0.992	0.147		0.234	0.089	0.164
TNM stage M	0.34	0.674	0.207	0.121		0.056	0.103
CD45-	0.723	0.736	0.302	0.563	0.716		0.726
CD45+	0.739	0.627	0.471	0.282	0.501	2E-08	

C

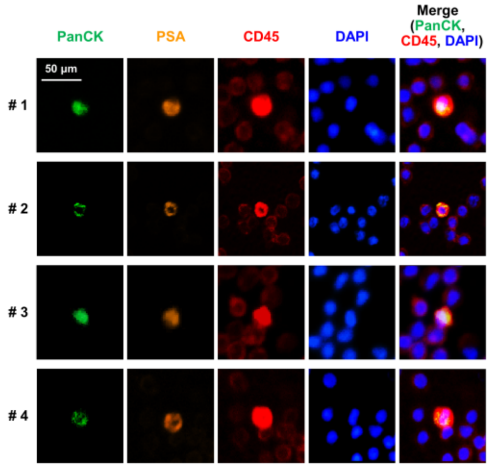

Sample number	Group	TNM stage			Age	PSA
1	T4N1M1	T4	N1	M1	67	72.95
2	N0M1	T3	N0	M1	74	418.8
3	T4N1M1	T4	N1	M1	65	546
4	M0	T4	N1	M0	60	570.2

Supplementary Figure S1-1. Clinical dataset, correlation analysis, and representative imaging of CTC-like cell subtypes.

A) Clinical characteristics and corresponding CTC-like cell counts, including $CD45^{+}$ and $CD45^{-}$ subsets, in individual metastatic prostate cancer patients.

B) Pearson correlation coefficients among clinical parameters (Age, PSA, and TNM stage) and the counts of $CD45^{+}$ and $CD45^{-}$ cytokeratin-positive CTC-like cells. The upper triangle shows correlation

coefficients, with color shading indicating the direction and strength of correlation: red for positive and blue for negative correlations. The lower triangle displays the corresponding p-values, with statistically significant correlations ($p < 0.05$) highlighted in green.

C) Representative immunofluorescence images of CTC-like cells isolated from patient blood samples, stained with PanCK (green), PSA (orange), CD45 (red), and DAPI (blue). The displayed images illustrate hybrid-like CTCs ($PanCK^{+}CD45^{+}$). Although epithelial-like CTCs ($PanCK^{+}CD45^{-}$) may also be present within patient samples, they were not observed in the representative fields shown here. Notably, PSA^{+} cells often appeared to co-express CD45, a phenomenon most likely arising from technical issues associated with the simultaneous four-color staining procedure rather than indicating a direct biological relationship between the two markers.

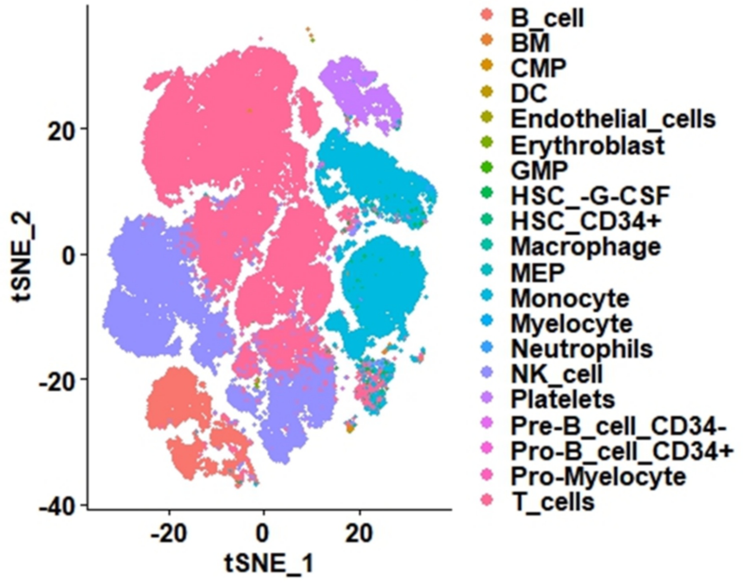

Annotation	Total (Count)	Type				All	
		KP_Pos		Others		KP_Pos	Others
		Count	Percent	Count	Percent	Percent	Percent
T_cells	30833	491	1.59	30342	98.41	0.759	46.902
NK_cell	14591	2	0.01	14589	99.99	0.003	22.551
Monocyte	11644	8	0.07	11636	99.93	0.012	17.986
B_cell	4611	51	1.11	4560	98.89	0.079	7.049
Platelets	2124	6	0.28	2118	99.72	0.009	3.274
HSC_-G-CSF	457	0	0.00	457	100.00	0	0.706
Pre-B_cell_CD34-	82	0	0.00	82	100.00	0	0.127
CMP	78	3	3.85	75	96.15	0.005	0.116
Erythroblast	75	0	0.00	75	100.00	0	0.116
Myelocyte	74	0	0.00	74	100.00	0	0.114
Neutrophils	41	0	0.00	41	100.00	0	0.063
BM	39	0	0.00	39	100.00	0	0.060
MEP	15	0	0.00	15	100.00	0	0.023
GMP	9	0	0.00	9	100.00	0	0.014
HSC_CD34+	7	0	0.00	7	100.00	0	0.011
Macrophage	6	0	0.00	6	100.00	0	0.009
Pro-Myelocyte	4	0	0.00	4	100.00	0	0.006
DC	1	0	0.00	1	100.00	0	0.002
Endothelial_cells	1	0	0.00	1	100.00	0	0.002
Pro-B_cell_CD34+	1	0	0.00	1	100.00	0	0.002

Supplementary Figure S1-2-1. SingleR-Based Cell Type Annotation and Distribution Analysis of PBMCs.

PBMCs from a patient with metastatic prostate cancer, profiled using single cell RNA sequencing, were clustered using the Seurat algorithm and annotated using SingleR, which assigns cell types by comparing transcriptomic profiles to a reference dataset.

Top: t-SNE plot showing clusters labeled with SingleR-predicted cell types.

Bottom: The table summarizes the number and percentage of each cell type in KP_Pos and Others groups. Highlighted cells indicate the counts and percentages for cell types present in the KP_Pos group.

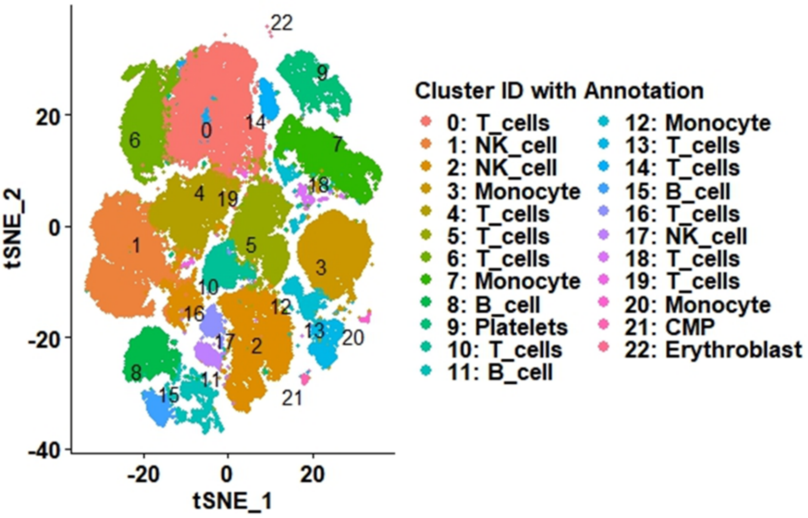

Annotations	Total (Count)	Type				All	
		KP_Pos		Others		KP_Pos	Others
		Count	Percent	Count	Percent	Percent	Percent
6: T_cells	4573	223	4.88	4350	95.12	0.348	6.783
0: T_cells	9522	216	2.27	9306	97.73	0.337	14.511
8: B_cell	2372	39	1.64	2333	98.36	0.061	3.638
4: T_cells	4940	14	0.28	4926	99.72	0.022	7.681
5: T_cells	4913	14	0.28	4899	99.72	0.022	7.639
14: T_cells	878	12	1.37	866	98.63	0.019	1.350
15: B_cell	777	10	1.29	767	98.71	0.016	1.196
9: T_cells	102	7	6.86	95	93.14	0.011	0.148
9: Platelets	2095	6	0.29	2089	99.71	0.009	3.257
3: Monocyte	5366	5	0.09	5361	99.91	0.008	8.359
11: B_cell	1407	2	0.14	1405	99.86	0.003	2.191
18: T_cells	197	2	1.02	195	98.98	0.003	0.304
19: T_cells	186	2	1.08	184	98.92	0.003	0.287
21: CMP	73	2	2.74	71	97.26	0.003	0.111
1: NK_cell	7495	1	0.01	7494	99.99	0.002	11.685
4: NK_cell	454	1	0.22	453	99.78	0.002	0.706
7: Monocyte	4167	1	0.02	4166	99.98	0.002	6.496
7: T_cells	28	1	3.57	27	96.43	0.002	0.042
11: Monocyte	63	1	1.59	62	98.41	0.002	0.097
11: CMP	1	1	100.00	0	0.00	0.002	0.000
12: Monocyte	1505	1	0.07	1504	99.93	0.002	2.345
0: NK_cell	6	0	0.00	6	100.00	0.000	0.009
0: Pre-B_cell_CD34-	1	0	0.00	1	100.00	0.000	0.002
1: T_cells	24	0	0.00	24	100.00	0.000	0.037
2: NK_cell	5387	0	0.00	5387	100.00	0.000	8.400
2: T_cells	2024	0	0.00	2024	100.00	0.000	3.156
2: Pre-B_cell_CD34-	8	0	0.00	8	100.00	0.000	0.012
2: HSC_-G-CSF	6	0	0.00	6	100.00	0.000	0.009
2: Platelets	4	0	0.00	4	100.00	0.000	0.006
2: B_cell	3	0	0.00	3	100.00	0.000	0.005
2: CMP	1	0	0.00	1	100.00	0.000	0.002
2: BM	1	0	0.00	1	100.00	0.000	0.002
3: HSC_-G-CSF	107	0	0.00	107	100.00	0.000	0.167
3: Pre-B_cell_CD34-	13	0	0.00	13	100.00	0.000	0.020
3: BM	13	0	0.00	13	100.00	0.000	0.020
3: Neutrophils	9	0	0.00	9	100.00	0.000	0.014
3: T_cells	7	0	0.00	7	100.00	0.000	0.011

Supplementary Figure S1-2-2. Unsupervised Cluster-to-Cell Type Mapping and KP_Pos

Distribution in PBMCs.

Based on the same PBMC dataset described in S1-2-1, cells were clustered using the Seurat algorithm. Cell type identities were assigned to each cluster based on transcriptomic similarity to reference profiles using an unsupervised cluster-to-cell type mapping approach.

Top: t-SNE plot showing Seurat clusters labeled with matched cell types.

Bottom: The table summarizes the number and percentage of each mapped cluster within the KP_Pos and Others groups.

Highlighted cells indicate the counts and percentages for clusters containing KP_Pos cells.

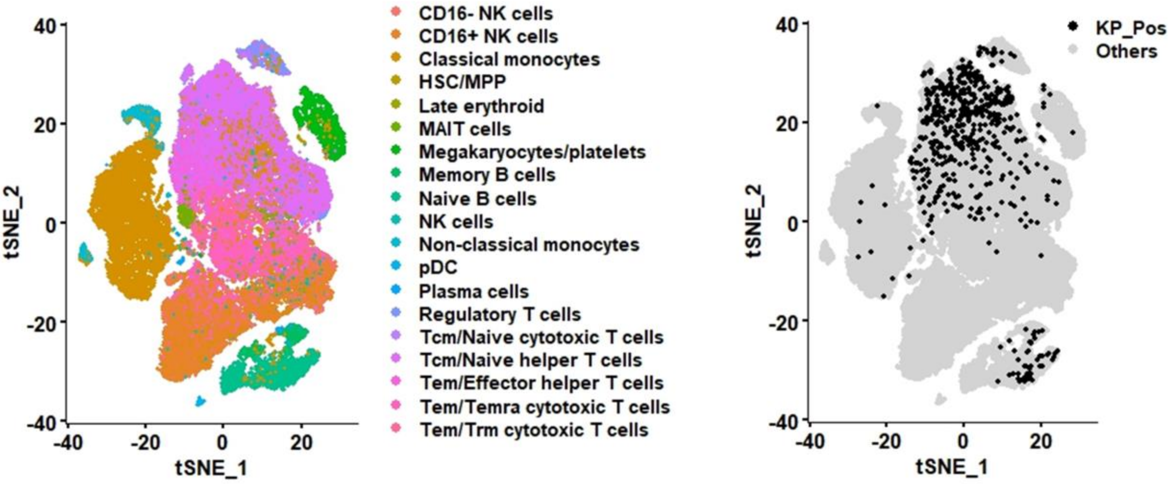

Annotation	Total (Count)	Type				All	
		KP_Pos		Others		KP_Pos	Others
		Count	Percent	Count	Percent	Percent	Percent
Tcm/Naive helper T cells	15115	359	2.38	14756	97.62	0.559	22.98
Tem/Effector helper T cells	4540	70	1.54	4470	98.46	0.109	6.96
Naive B cells	3444	40	1.16	3404	98.84	0.062	5.30
Tem/Trm cytotoxic T cells	3265	26	0.80	3239	99.20	0.040	5.04
Regulatory T cells	1391	17	1.22	1374	98.78	0.026	2.14
Memory B cells	1139	11	0.97	1128	99.03	0.017	1.76
Classical monocytes	11855	10	0.08	11845	99.92	0.016	18.44
Tcm/Naive cytotoxic T cells	472	9	1.91	463	98.09	0.014	0.72
Megakaryocytes/platelets	2097	5	0.24	2092	99.76	0.008	3.26
MAIT cells	658	5	0.76	653	99.24	0.008	1.02
CD16+ NK cells	11084	4	0.04	11080	99.96	0.006	17.25
HSC/MPP	142	3	2.11	139	97.89	0.005	0.22
Tem/Temra cytotoxic T cells	6915	1	0.01	6914	99.99	0.002	10.77
Non-classical monocytes	1431	1	0.07	1430	99.93	0.002	2.23
NK cells	579	0	0.00	579	100.00	0.000	0.90
CD16- NK cells	297	0	0.00	297	100.00	0.000	0.46
Plasma cells	172	0	0.00	172	100.00	0.000	0.27
pDC	153	0	0.00	153	100.00	0.000	0.24
Late erythroid	32	0	0.00	32	100.00	0.000	0.05

Supplementary Figure S1-2-3. Celltypist-based annotation and cell type distribution of PBMCs.

Based on the same PBMC dataset described in S1-2-1, cell type annotation was performed using Celltypist, an automated reference-based classification tool.

Left: t-SNE plot labeled with Celltypist-derived cell types.

Right: The table shows the number and percentage of each annotated cell type within the KP_Pos and Others groups.

Highlighted cells indicate the values corresponding to cell types present in the KP_Pos group.

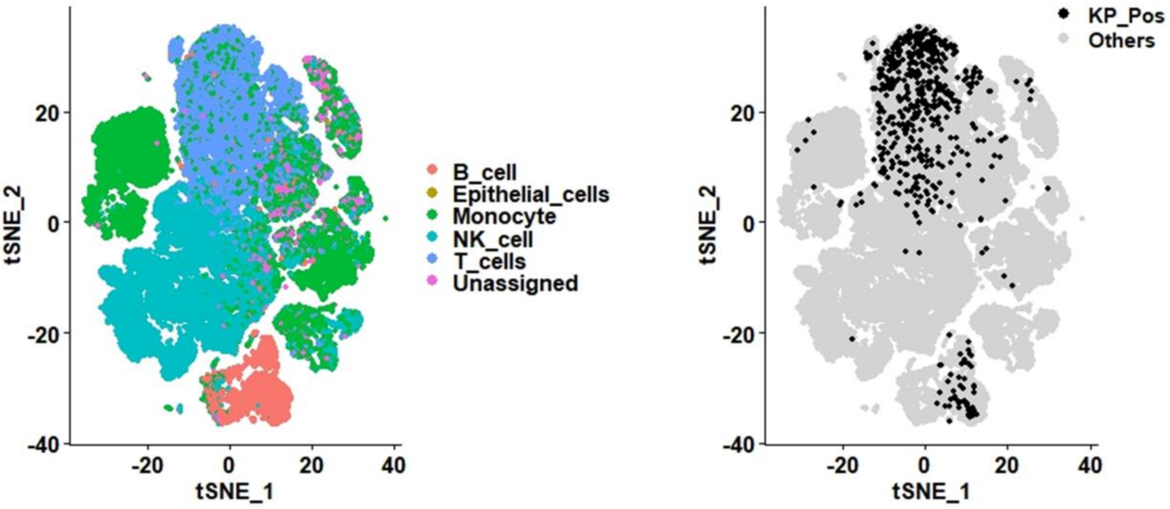

Annotation	Total (Count)	Type				All	
		KP_Pos		Others		KP_Pos	Others
		Count	Percent	Count	Percent	Percent	Percent
T_cells	15118	392	2.59	14726	97.41	0.61	22.73
Monocyte	20549	76	0.37	20473	99.63	0.12	31.60
B_cell	4676	54	1.15	4622	98.85	0.08	7.13
NK_cell	21351	26	0.12	21325	99.88	0.04	32.92
Epithelial_cells	73	13	17.81	60	82.19	0.02	0.09
Unassigned	3014	0	0.00	3014	100.00	0.00	4.65

Supplementary Figure S1-2-4. Marker-based annotation and cell type distribution of PBMCs.

Using the same PBMC dataset as in S1-2-1, cell types were annotated based on the expression of canonical cell type–specific marker genes.

Top: t-SNE plot labeled with marker-inferred cell types.

Bottom: The table summarizes the number and percentage of each marker-defined cell type in the KP_Pos and Others groups.

Highlighted cells represent cell types detected in the KP_Pos group.

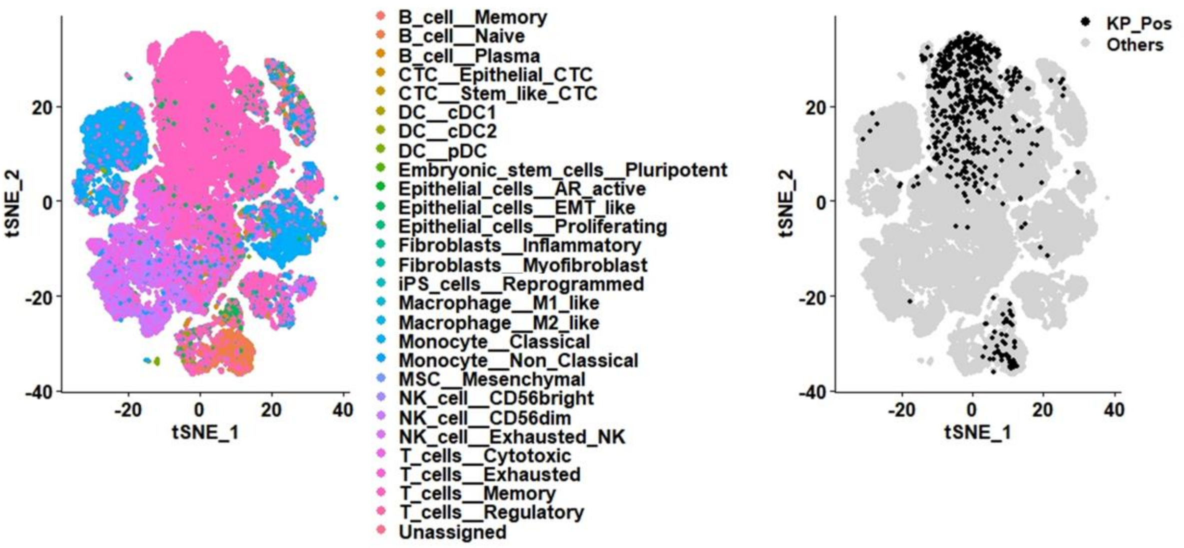

Annotation	Total (Count)	Type				All	
		KP_Pos		Others		KP_Pos	Others
		Count	Percent	Count	Percent	Percent	Percent
T_cells__Memory	32980	515	1.562	32465	98.438	0.795	50.115
B_cell__Naive	2004	29	1.447	1975	98.553	0.045	3.049
Epithelial_cells__EMT_like	1631	7	0.429	1624	99.571	0.011	2.507
Monocyte__Classical	9173	6	0.065	9167	99.935	0.009	14.151
Monocyte__Non_Classical	3252	2	0.062	3250	99.938	0.003	5.017
CTC__Epithelial_CTC	35	1	2.857	34	97.143	0.002	0.052
T_cells__Exhausted	543	1	0.184	542	99.816	0.002	0.837
B_cell__Memory	84	0	0.000	84	100.000	0.000	0.130
B_cell__Plasma	218	0	0.000	218	100.000	0.000	0.337
CTC__Stem_like_CTC	334	0	0.000	334	100.000	0.000	0.516
DC__cDC1	6	0	0.000	6	100.000	0.000	0.009
DC__cDC2	57	0	0.000	57	100.000	0.000	0.088
DC__pDC	226	0	0.000	226	100.000	0.000	0.349
Embryonic_stem_cells__Pluripotent	2	0	0.000	2	100.000	0.000	0.003
Epithelial_cells__AR_active	22	0	0.000	22	100.000	0.000	0.034
Epithelial_cells__Proliferating	57	0	0.000	57	100.000	0.000	0.088
Fibroblasts__Inflammatory	1	0	0.000	1	100.000	0.000	0.002
Fibroblasts__Myofibroblast	5	0	0.000	5	100.000	0.000	0.008
MSC__Mesenchymal	7	0	0.000	7	100.000	0.000	0.011
Macrophage__M1_like	62	0	0.000	62	100.000	0.000	0.096
Macrophage__M2_like	11	0	0.000	11	100.000	0.000	0.017
NK_cell__CD56bright	168	0	0.000	168	100.000	0.000	0.259
NK_cell__CD56dim	5461	0	0.000	5461	100.000	0.000	8.430
NK_cell__Exhausted_NK	7	0	0.000	7	100.000	0.000	0.011
T_cells__Cytotoxic	7503	0	0.000	7503	100.000	0.000	11.582
T_cells__Regulatory	143	0	0.000	143	100.000	0.000	0.221
Unassigned	680	0	0.000	680	100.000	0.000	1.050
iPS_cells__Reprogrammed	109	0	0.000	109	100.000	0.000	0.168

Supplementary Figure S1-2-5. Subtype-specific marker-based annotation and cell subtype distribution of PBMCs.

Based on the PBMC dataset described in S1-2-1, cell subtypes were annotated using curated marker gene sets specific to functional subpopulations.

Top: t-SNE plot showing annotated immune and epithelial subtypes.

Bottom: The table presents the number and percentage of each cell subtype in the KP_Pos and Others groups.

Highlighted cells indicate subtypes found in the KP_Pos group.

Counts and percentages of KP_Pos and Other cells in PBMCs from M1-stage prostate cancer patients

No	Sample	Annotation	Total (Count)	Type				All	
				KP_Pos		Others		KP_Pos	Others
				Count	Percent	Count	Percent	Percent	Percent
1	PBMC#1	T_cells	4870	71	1.46	4799	98.54	0.73	49.05
		B_cell	1553	12	0.77	1541	99.23	0.12	15.75
		NK_cell	2051	1	0.05	2050	99.95	0.01	20.95
		Platelets	351	1	0.28	350	99.72	0.01	3.58
		Monocyte	959	0	0	959	100	0	9.80
		Overall Summary	9784	85		9699		0.87	99.13
2	PBMC#2	T_cells	5838	74	1.27	5764	98.73	0.65	50.31
		B_cell	831	7	0.84	824	99.16	0.06	7.19
		NK_cell	2590	0	0	2590	100	0	22.61
		Monocyte	2173	0	0	2173	100	0	18.97
		Platelets	25	0	0	25	100	0	0.22
		Overall Summary	11457	81		11376		0.71	99.29
3	PBMC#3	T_cells	5396	110	2.04	5286	97.96	0.96	46.35
		B_cell	945	20	2.12	925	97.88	0.18	8.11
		Monocyte	866	3	0.35	863	99.65	0.03	7.57
		Platelets	629	1	0.16	628	99.84	0.01	5.51
		NK_cell	3569	0	0	3569	100	0	31.29
		Overall Summary	11405	134		11271		1.17	98.83
4	PBMC#4	T_cells	5825	111	1.91	5714	98.09	0.95	48.88
		B_cell	755	7	0.93	748	99.07	0.06	6.40
		Platelets	379	4	1.06	375	98.94	0.03	3.21
		NK_cell	3698	0	0	3698	100	0	31.63
		Monocyte	1034	0	0	1034	100	0	8.84
		Overall Summary	11691	122		11569		1.04	98.96
5	PBMC#5	T_cells	3676	103	2.80	3573	97.20	1.71	59.31
		B_cell	409	4	0.98	405	99.02	0.07	6.72
		Monocyte	499	1	0.20	498	99.80	0.02	8.27
		Platelets	234	1	0.43	233	99.57	0.02	3.87
		NK_cell	1116	0	0	1116	100	0	18.53
		Myelocyte	71	0	0	71	100	0	1.18
		BM	19	0	0	19	100	0	0.32
		Overall Summary	6024	109		5915		1.81	98.19
6	PBMC#6	T_cells	1559	20	1.28	1539	98.72	0.68	52.53
		Platelets	528	1	0.19	527	99.81	0.03	17.99
		B_cell	70	1	1.43	69	98.57	0.03	2.35
		NK_cell	530	0	0	530	100	0	18.09
		Monocyte	243	0	0	243	100	0	8.29
		Overall Summary	2930	22		2908		0.75	99.25
7	PBMC#7	Monocyte	6483	5	0.08	6478	99.92	0.04	56.67
		CMP	123	2	1.63	121	98.37	0.02	1.06
		B_cell	224	1	0.45	223	99.55	0.01	1.95
		T_cells	3415	0	0	3415	100	0	29.87
		NK_cell	1000	0	0	1000	100	0	8.75
		Erythroblast	187	0	0	187	100	0	1.64
		Overall Summary	11432	8		11424		0.07	99.93

Supplementary Figure S1-3-1. Counts and percentages of KP_Pos and Other cells in PBMCs from seven M1-stage prostate cancer patients analyzed by single-cell RNA sequencing.

PBMC#1

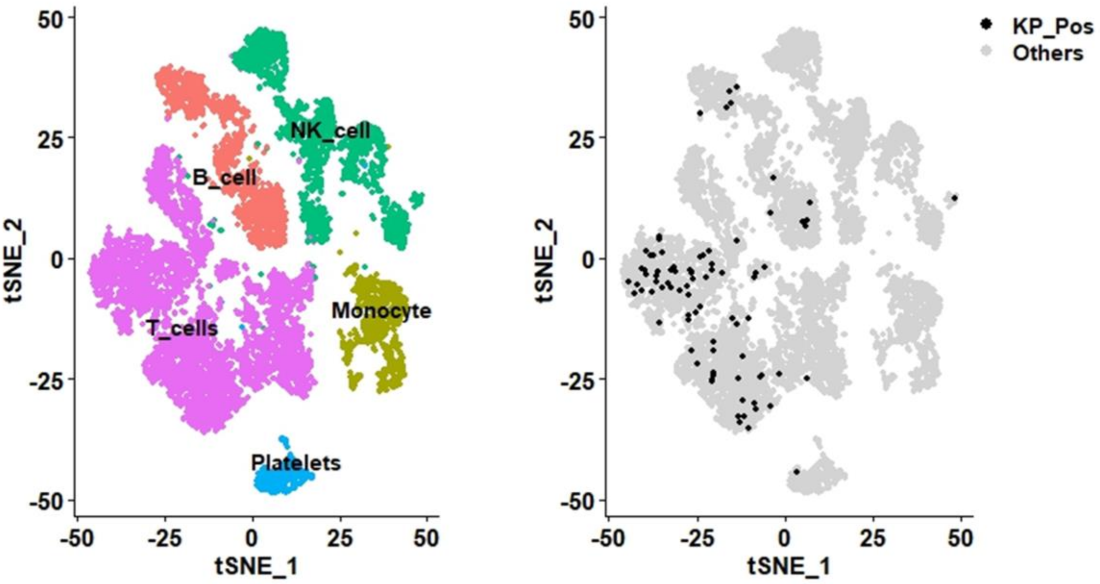

Annotation	Total (Count)	Type				All	
		KP_Pos		Others		KP_Pos	Others
		Count	Percent	Count	Percent	Percent	Percent
T_cells	4870	71	1.46	4799	98.54	0.73	49.05
B_cell	1553	12	0.77	1541	99.23	0.12	15.75
NK_cell	2051	1	0.05	2050	99.95	0.01	20.95
Platelets	351	1	0.28	350	99.72	0.01	3.58
Monocyte	959	0	0.00	959	100.00	0.00	9.80

Supplementary Figure S1-3-2-1. t-SNE-based clustering and majority voting-based annotation of PBMCs from M1-stage patient #1.

PBMC#2

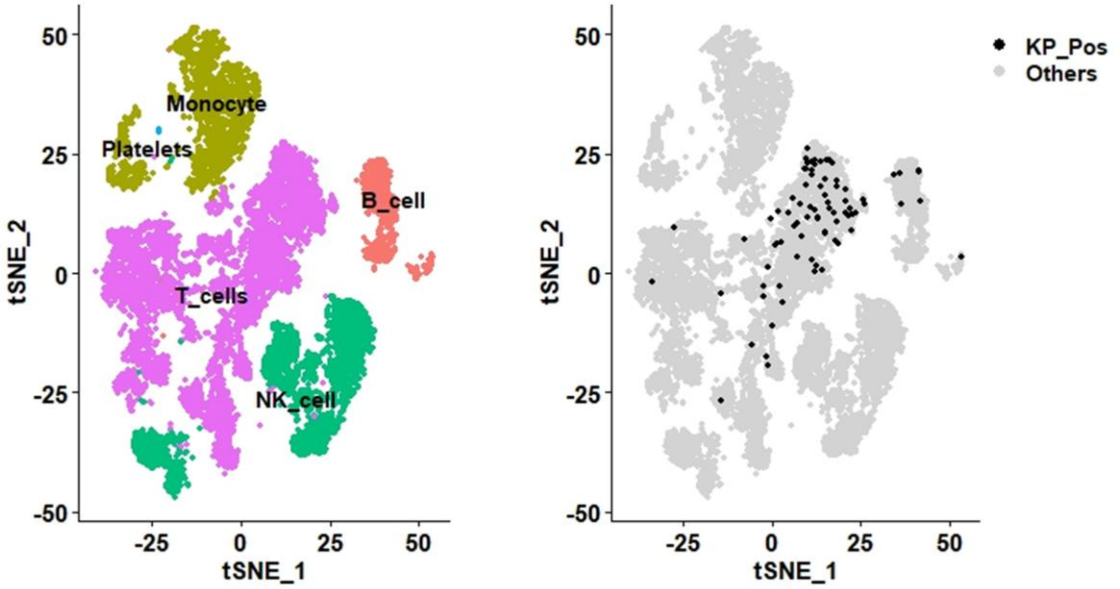

Annotation	Total (Count)	Type				All	
		KP_Pos		Others		KP_Pos	Others
		Count	Percent	Count	Percent	Percent	Percent
T_cells	5838	74	1.27	5764	98.73	0.65	50.31
B_cell	831	7	0.84	824	99.16	0.06	7.19
NK_cell	2590	0	0.00	2590	100.00	0.00	22.61
Monocyte	2173	0	0.00	2173	100.00	0.00	18.97
Platelets	25	0	0.00	25	100.00	0.00	0.22

Supplementary Figure S1-3-2-2. t-SNE-based clustering and majority voting-based annotation of PBMCs from M1-stage patient #2.

PBMC#3

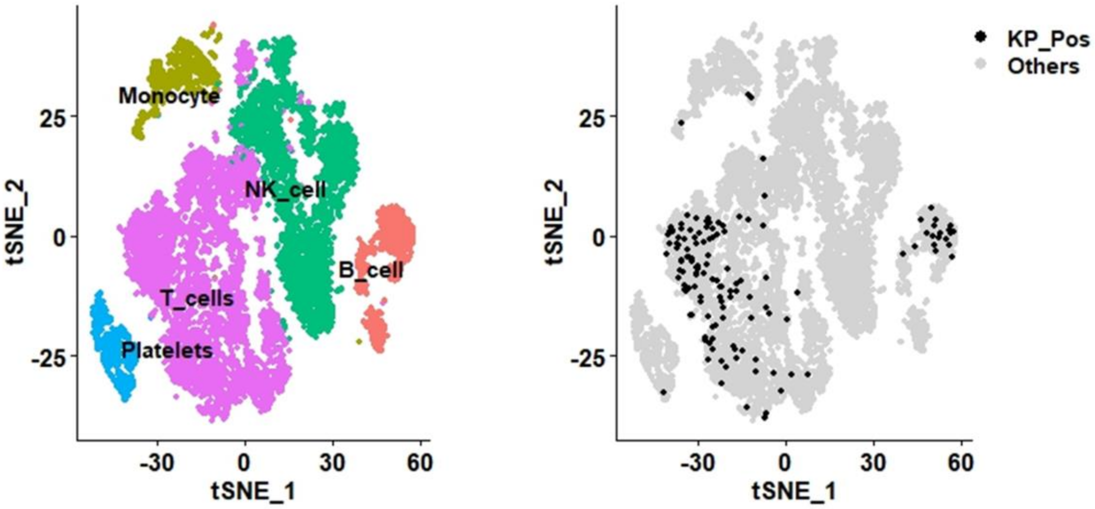

Annotation	Total (Count)	Type				All	
		KP_Pos		Others		KP_Pos	Others
		Count	Percent	Count	Percent	Percent	Percent
T_cells	5396	110	2.04	5286	97.96	0.964	46.35
B_cell	945	20	2.12	925	97.88	0.175	8.11
Monocyte	866	3	0.35	863	99.65	0.026	7.57
Platelets	629	1	0.16	628	99.84	0.009	5.51
NK_cell	3569	0	0	3569	100	0	31.29

Supplementary Figure S1-3-2-3. t-SNE-based clustering and majority voting-based annotation of PBMCs from M1-stage patient #3.

PBMC#4

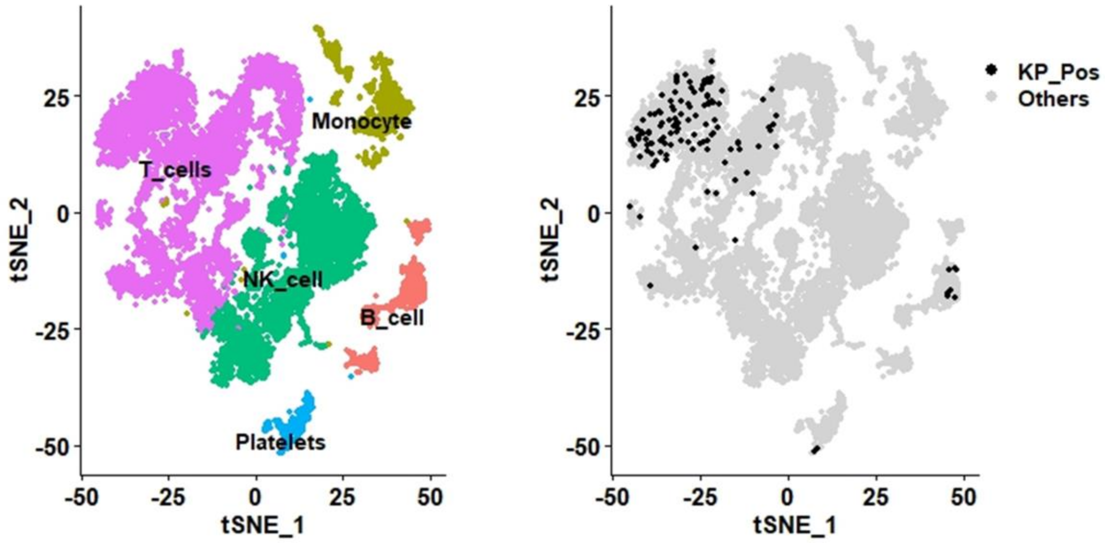

Annotation	Total (Count)	Type				All	
		KP_Pos		Others		KP_Pos	Others
		Count	Percent	Count	Percent	Percent	Percent
T_cells	5825	111	1.91	5714	98.09	0.95	48.88
B_cell	755	7	0.93	748	99.07	0.06	6.40
Platelets	379	4	1.06	375	98.94	0.03	3.21
NK_cell	3698	0	0.00	3698	100.00	0.00	31.63
Monocyte	1034	0	0.00	1034	100.00	0.00	8.84

Supplementary Figure S1-3-2-4. t-SNE-based clustering and majority voting-based annotation of PBMCs from M1-stage patient #4.

PBMC#5

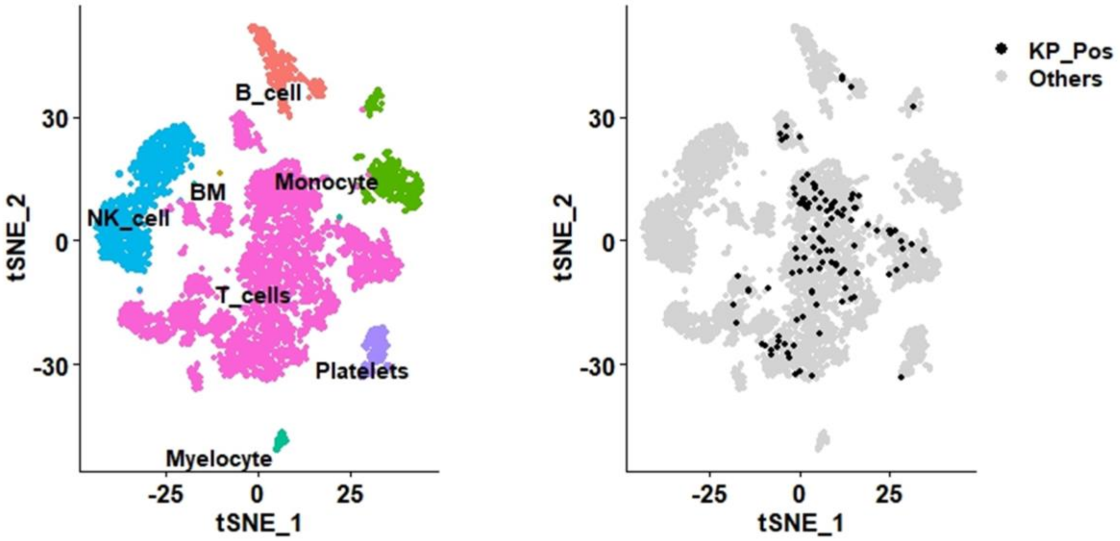

Annotation	Total (Count)	Type				All	
		KP_Pos		Others		KP_Pos	Others
		Count	Percent	Count	Percent	Percent	Percent
T_cells	3676	103	2.80	3573	97.20	1.71	59.31
B_cell	409	4	0.98	405	99.02	0.07	6.72
Monocyte	499	1	0.20	498	99.80	0.02	8.27
Platelets	234	1	0.43	233	99.57	0.02	3.87
NK_cell	1116	0	0.00	1116	100.00	0.00	18.53
Myelocyte	71	0	0.00	71	100.00	0.00	1.18
BM	19	0	0.00	19	100.00	0.00	0.32

Supplementary Figure S1-3-2-5. t-SNE-based clustering and majority voting-based annotation of PBMCs from M1-stage patient #5.

PBMC#6

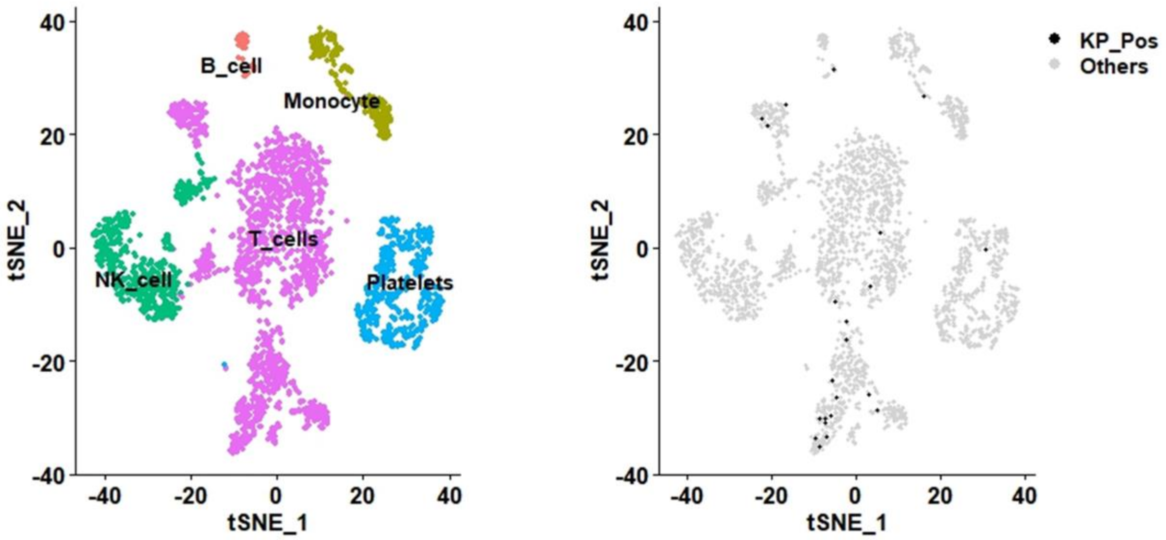

Annotation	Total (Count)	Type				All	
		KP_Pos		Others		KP_Pos	Others
		Count	Percent	Count	Percent	Percent	Percent
T_cells	1559	20	1.28	1539	98.72	0.68	98.72
Platelets	528	1	0.19	527	99.81	0.03	33.80
B_cell	70	1	1.43	69	98.57	0.03	4.43
NK_cell	530	0	0.00	530	100.00	0.00	34.00
Monocyte	243	0	0.00	243	100.00	0.00	15.59

Supplementary Figure S1-3-2-6. t-SNE-based clustering and majority voting-based annotation of PBMCs from M1-stage patient #6.

PBMC#7

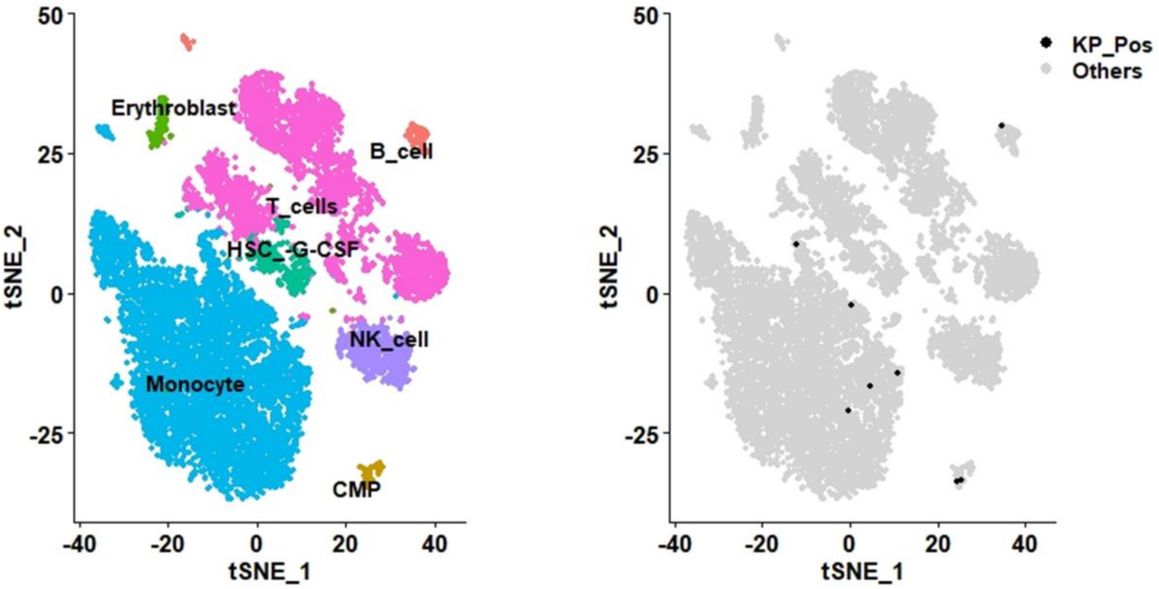

Annotation	Total (Count)	Type				All	
		KP_Pos		Others		KP_Pos	Others
		Count	Percent	Count	Percent	Percent	Percent
Monocyte	6483	5	0.08	6478	99.92	0.04	56.67
CMP	123	2	1.63	121	98.37	0.02	1.06
B_cell	224	1	0.45	223	99.55	0.01	1.95
T_cells	3415	0	0.00	3415	100.00	0.00	29.87
NK_cell	1000	0	0.00	1000	100.00	0.00	8.75
Erythroblast	187	0	0.00	187	100.00	0.00	1.64

Supplementary Figure S1-3-2-7. t-SNE-based clustering and majority voting-based annotation of PBMCs from M1-stage patient #7.

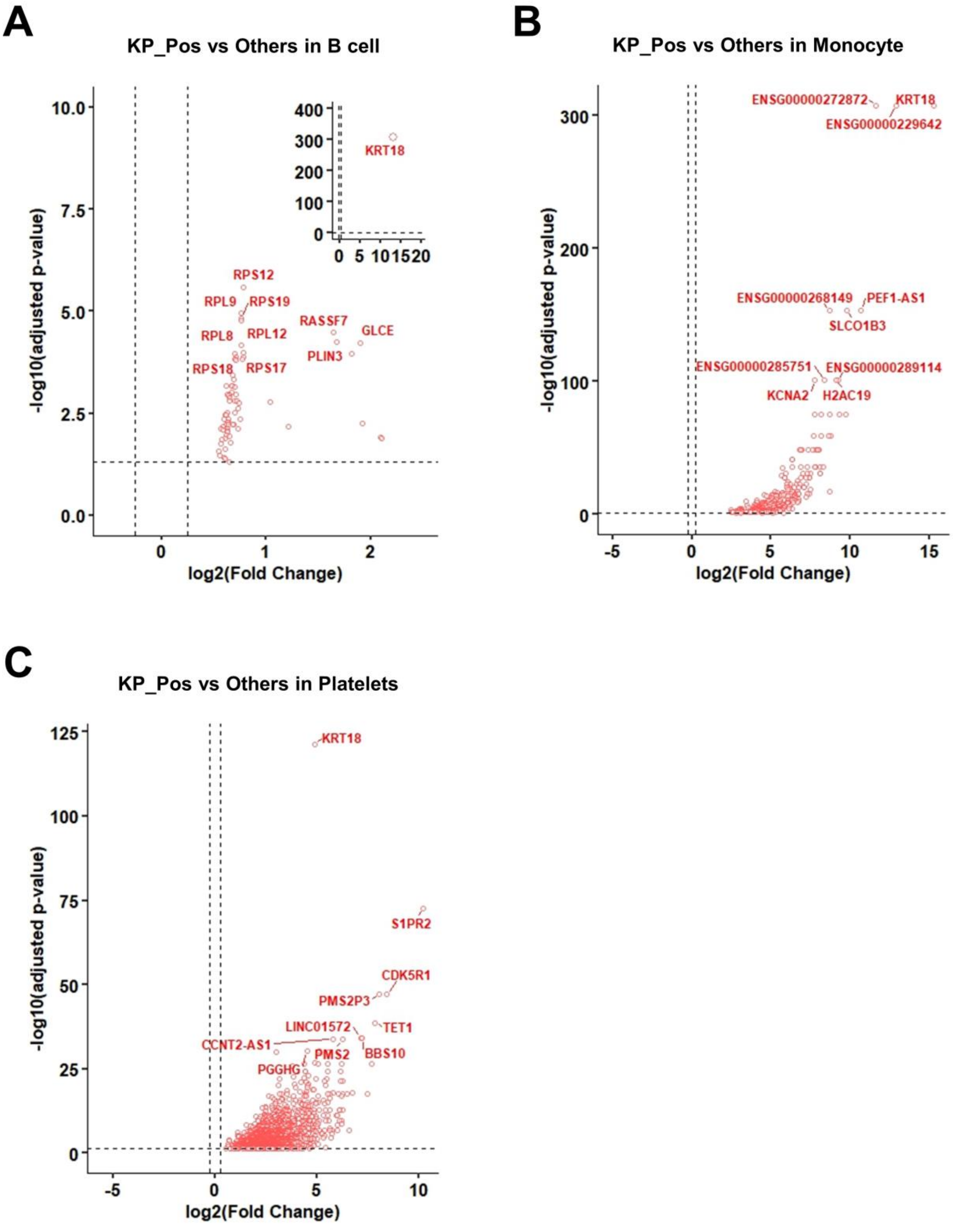

Supplementary Figure S1-4. Differential gene expression analyses of KP_Pos versus Other cells within specific immune lineages from PBMCs of M1-stage prostate cancer patients.

(A) B cells, (B) Monocytes, and (C) Platelets were analyzed separately using the same statistical thresholds as applied to the integrated PBMC dataset (log_2 fold change > 0.25, adjusted $p < 0.05$).

Significantly upregulated genes in KP_Pos cells are shown in red, and only the Top 10 upregulated genes are labeled in each panel.

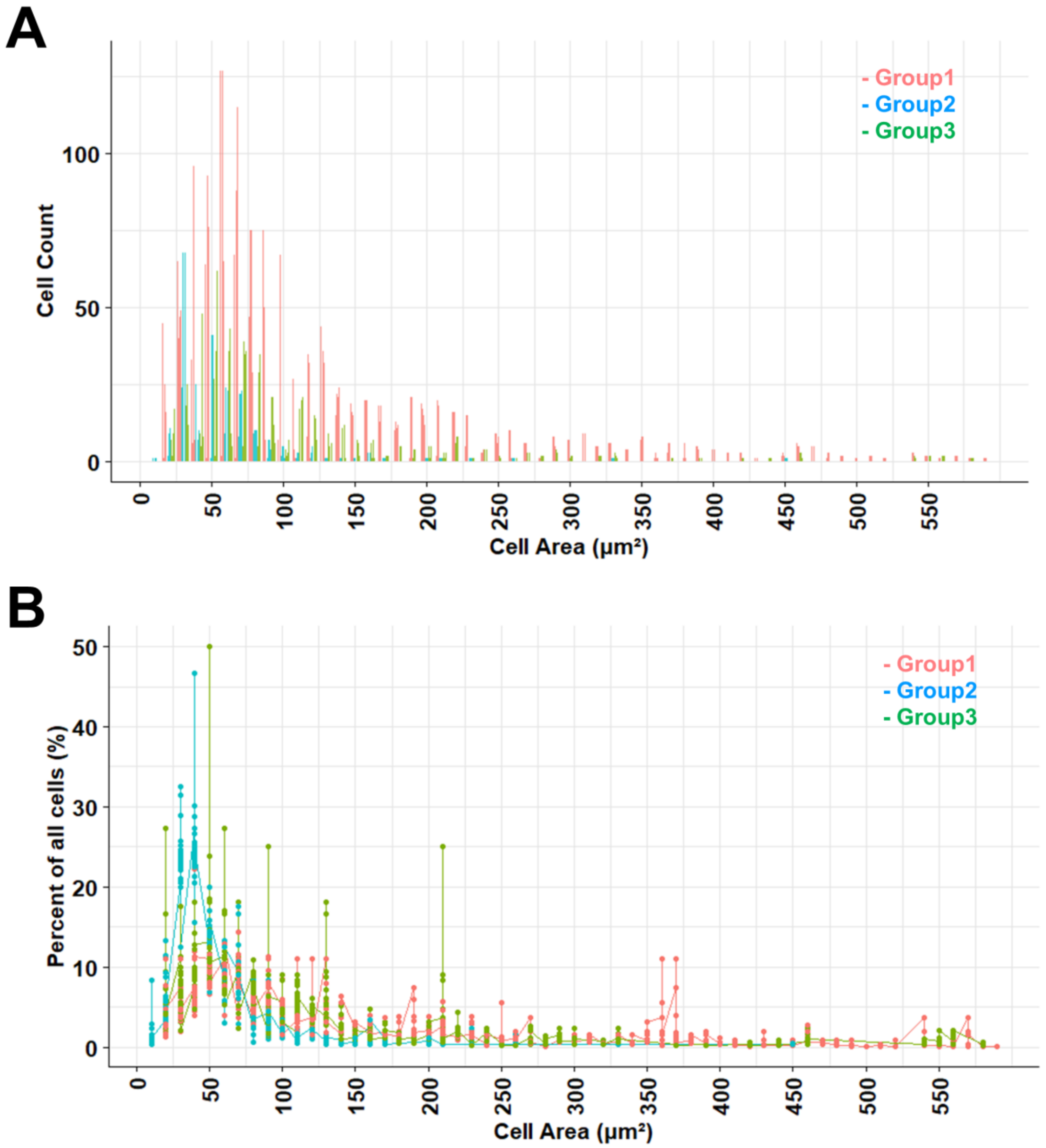

Supplementary Figure S2-1-1-1. Full distribution of total cell area of CK⁺CD45⁺ cells across patient groups.

(A) Histogram showing the absolute counts of $CK^{+}CD45^{+}$ cells according to total cell area (μm²) in Group 1 (red), Group 2 (blue), and Group 3 (green).

(B) Line plot with overlaid points showing the relative frequency (%) of $CK^{+}CD45^{+}$ cells across the same cell area bins.

For clarity, the main Figure 2B-a displays data truncated at 250 μm², whereas this supplementary figure presents the complete distribution across the full measurement range. These supplementary plots are provided to facilitate interpretation of the dual bar–line plots in the main figure by separately visualizing absolute and relative distributions.

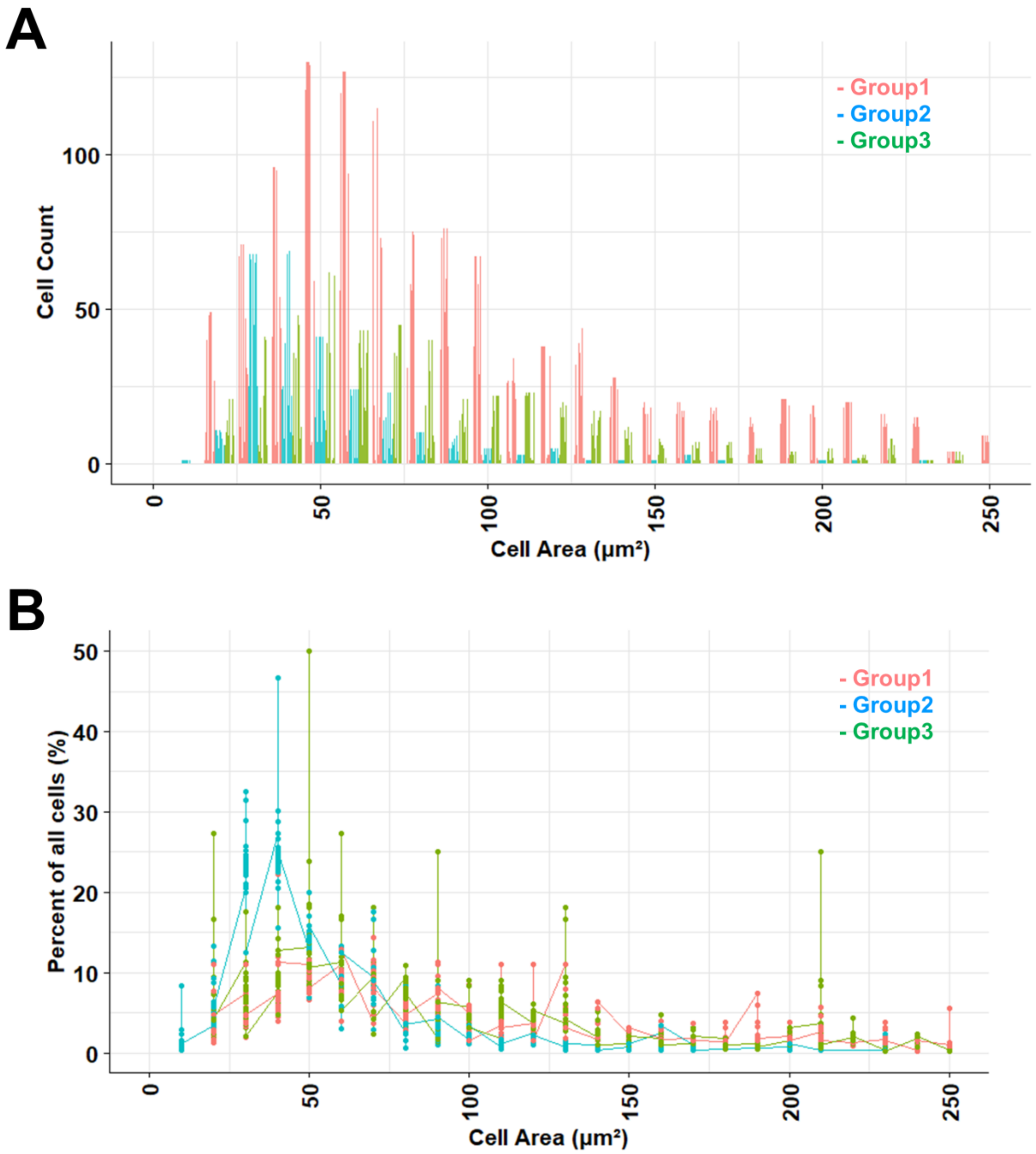

Supplementary Figure S2-1-1-2. Focused distribution of total cell area (≤ 250 μm²) of $CK^{+}CD45^{+}$ cells, shown separately as absolute counts and relative frequencies.

(A) Histogram showing the absolute counts of $CK^{+}CD45^{+}$ cells within the restricted total-area range, comparing Group 1 (red), Group 2 (blue), and Group 3 (green).

(B) Line plots showing the relative frequency (%) of $CK^{+}CD45^{+}$ cells across the same range, highlighting finer group-wise differences in smaller-size categories.

This supplementary figure corresponds to Figure 2B-a and presents the same dataset in a separated form, facilitating clearer interpretation of absolute versus relative size distributions within the restricted range.

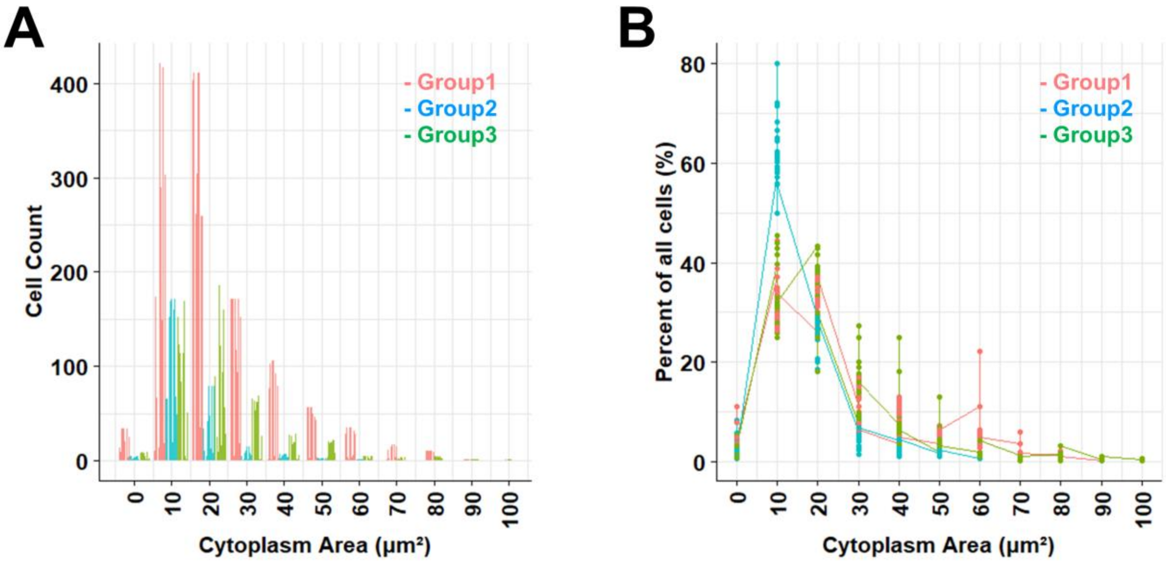

Supplementary Figure S2-1-2. Distribution of cytoplasmic area of CK⁺CD45⁺ cells shown separately as absolute counts and relative frequencies.

(A) Histogram of absolute counts of CK⁺CD45⁺ cells according to cytoplasmic area (μm²) in Group 1 (red), Group 2 (blue), and Group 3 (green).

(B) Line plot with points showing the relative frequency (%) of CK⁺CD45⁺ cells across cytoplasmic area bins. The majority of CK⁺CD45⁺ cells exhibited cytoplasmic areas <30 μm², with notable distributional differences between groups.

As with total cell area, this supplementary figure separates absolute and relative plots to aid in interpreting the dual presentation shown in the main Figure 2B-b.

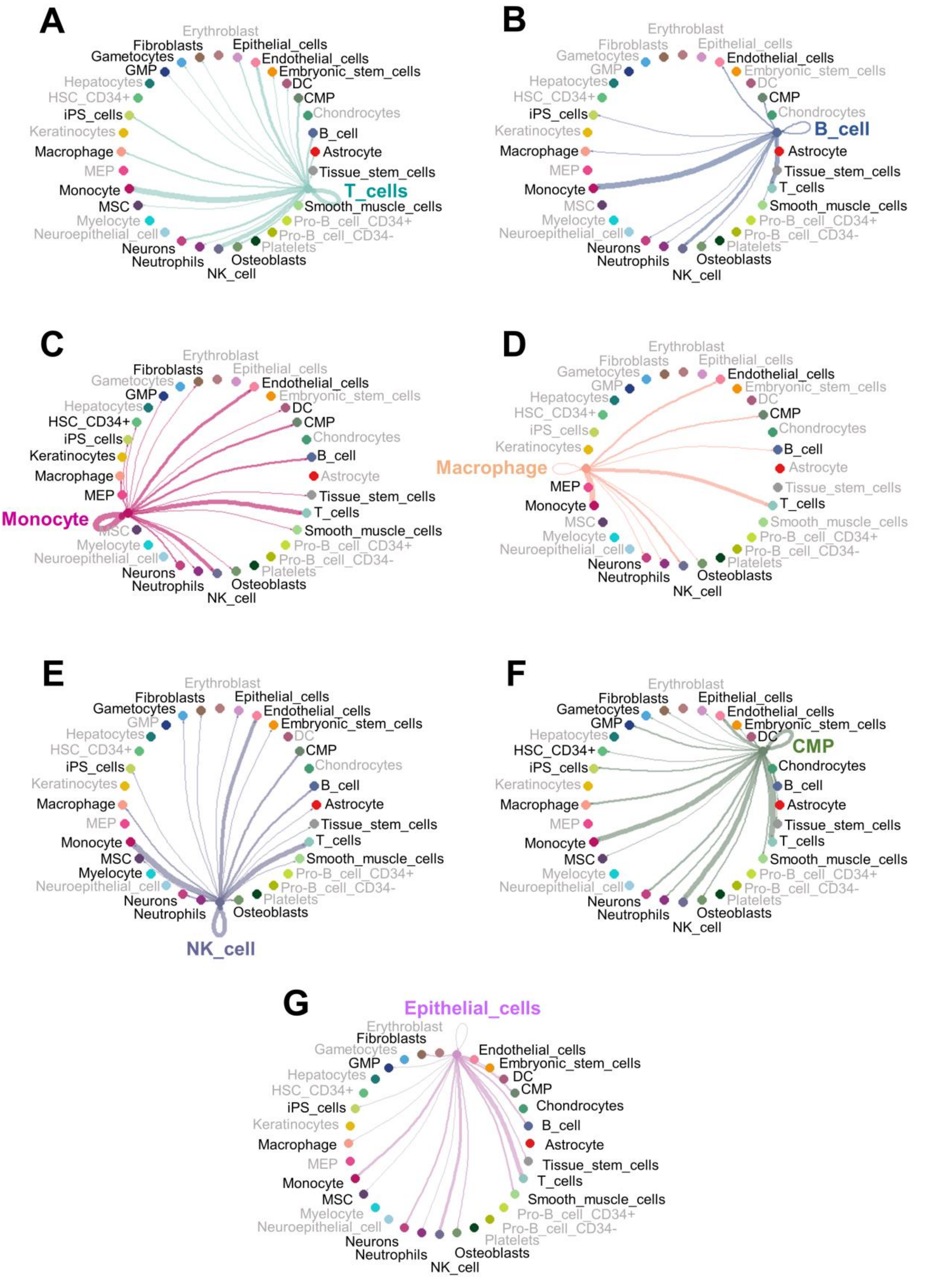

Supplementary Figure S2-2-1-1. Outgoing cell–cell communication profiles of major immune and

epithelial cell types.

CellChat analysis showing outgoing signaling interactions from (A) T cells, (B) B cells, (C) Monocytes, (D) Macrophages, (E) NK cells, (F) CMPs, and (G) Epithelial cells in metastatic prostate cancer. Line thickness represents the relative strength of inferred communication between the sender (center) and target populations.

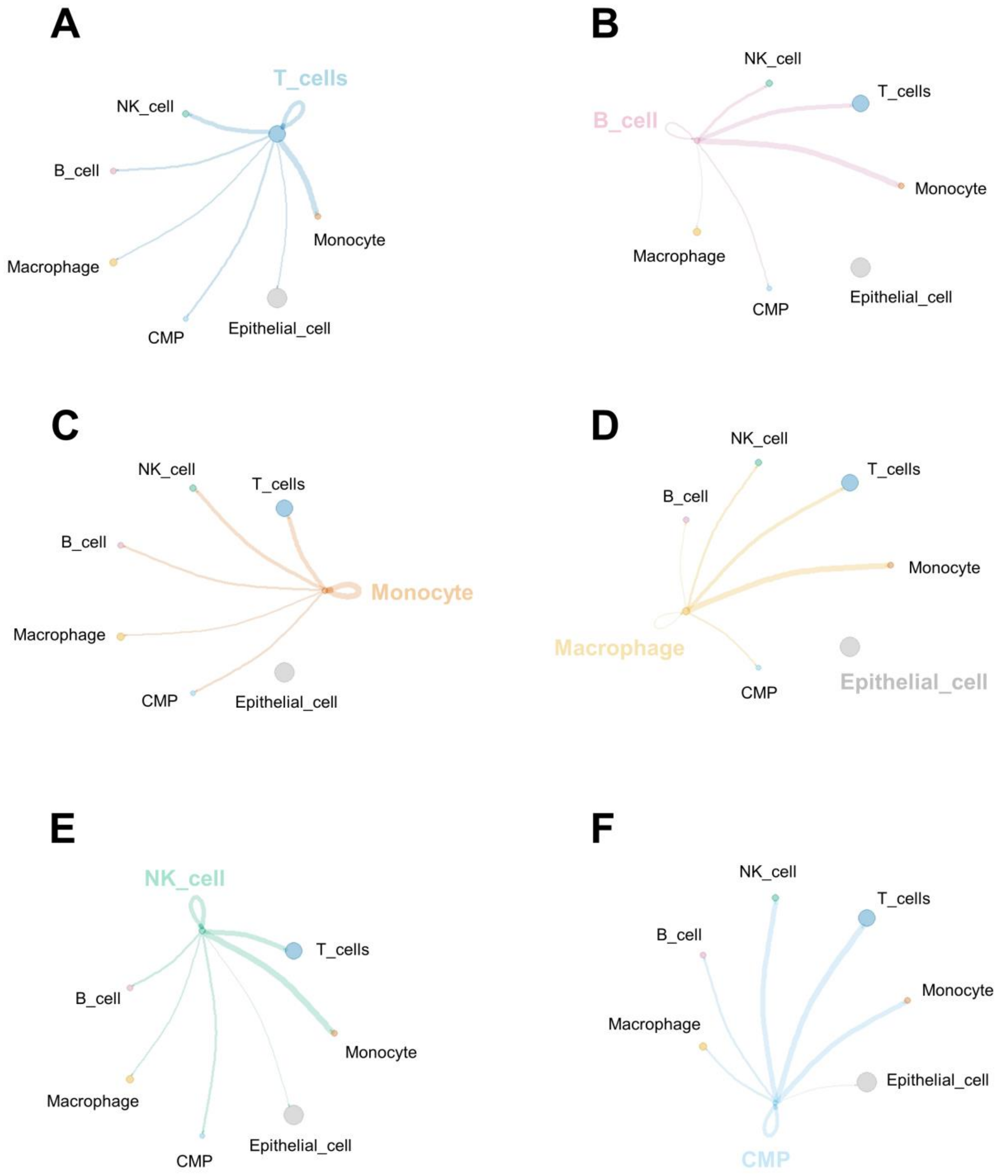

Supplementary Figure S2-2-1-2. Simplified outgoing signaling networks among major immune subsets.

Circle plots showing outgoing intercellular communication from six major immune populations—(A) T cells, (B) B cells, (C) Monocytes, (D) Macrophages, (E) NK cells, and (F) CMPs—to other immune and epithelial populations in metastatic prostate cancer. This figure corresponds to Supplementary Figure S2-2-1-1 but is simplified and restricted to the major immune subsets for clearer visualization

of dominant signaling connections. Line thickness represents the relative strength of inferred outgoing signaling.

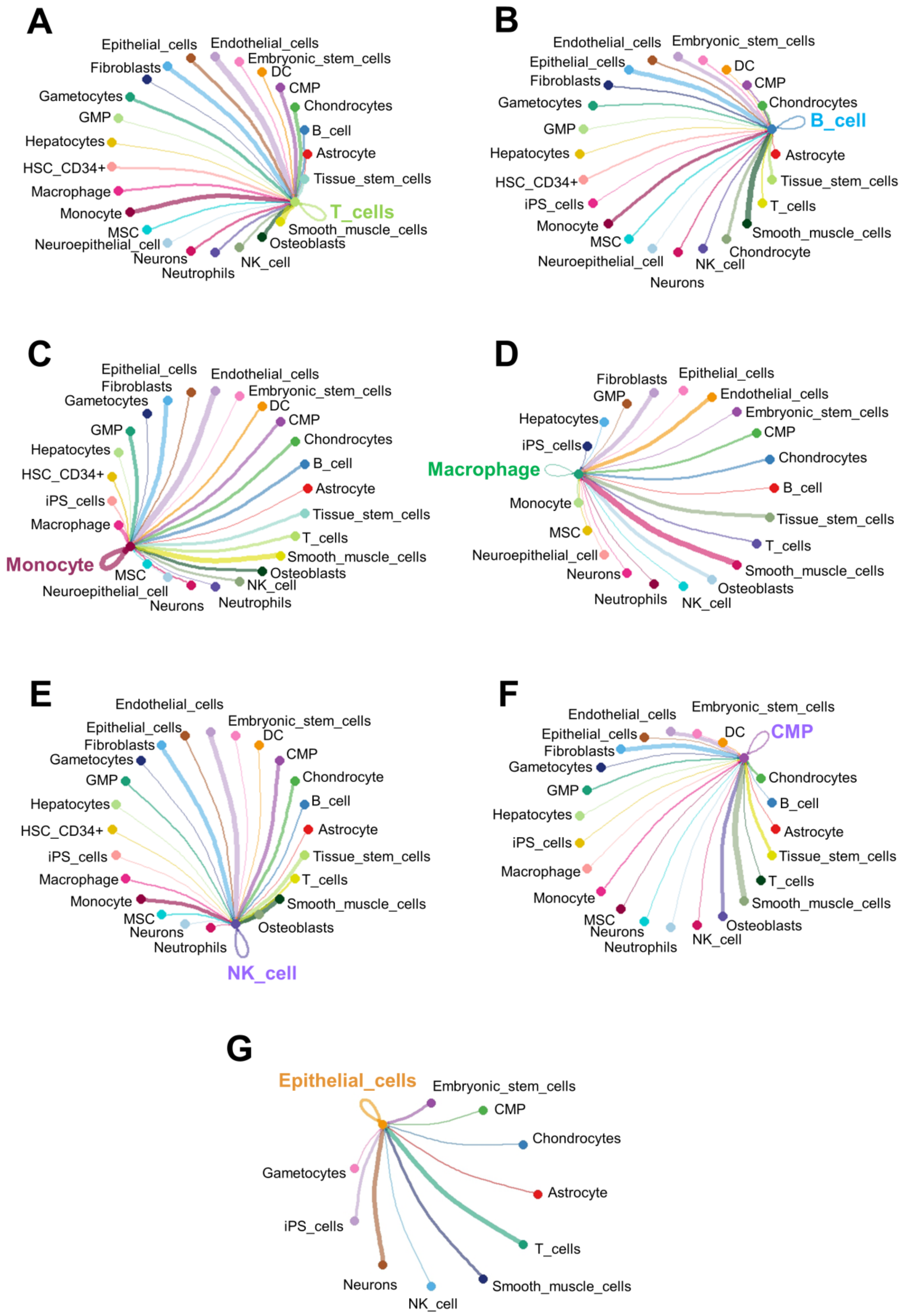

A
Epithelial_cells
Endothelial_cells
Fibroblasts
Embryonic_stem_cells
DC
Gametocytes
CMP
GMP
Chondrocytes
Hepatocytes
B_cell
HSC_CD34+
Astrocyte
Macrophage
Tissue_stem_cells
Monocyte
T_cells
MSC
Smooth_muscle_cells
Neuroepithelial_cell
Osteoblasts
Neurons
NK_cell
Neutrophils
B
Endothelial_cells
Embryonic_stem_cells
Epithelial_cells
DC
Fibroblasts
CMP
Gametocytes
Chondrocytes
B_cell
GMP
Hepatocytes
Astrocyte
HSC_CD34+
Tissue_stem_cells
iPS_cells
T_cells
Monocyte
Smooth_muscle_cells
MSC
Chondrocyte
Neuroepithelial_cell
NK_cell
Neurons
C
Epithelial_cells
Endothelial_cells
Fibroblasts
Embryonic_stem_cells
Gametocytes
DC
CMP
GMP
Chondrocytes
Hepatocytes
B_cell
HSC_CD34+
Astrocyte
iPS_cells
Tissue_stem_cells
Macrophage
T_cells
Smooth_muscle_cells
Monocyte
Osteoblasts
MSC
NK_cell
Neuroepithelial_cell
Neurons
Neutrophils
D
Fibroblasts
Epithelial_cells
GMP
Endothelial_cells
Hepatocytes
Embryonic_stem_cells
CMP
iPS_cells
Chondrocytes
Macrophage
B_cell
Monocyte
Tissue_stem_cells
MSC
T_cells
Neuroepithelial_cell
Smooth_muscle_cells
Neurons
Osteoblasts
Neutrophils
NK_cell
E
Endothelial_cells
Epithelial_cells
Embryonic_stem_cells
Fibroblasts
DC
Gametocytes
CMP
GMP
Chondrocyte
B_cell
Hepatocytes
Astrocyte
HSC_CD34+
iPS_cells
Tissue_stem_cells
Macrophage
T_cells
Monocyte
Smooth_muscle_cells
MSC
Osteoblasts
Neurons
Neutrophils
NK_cell
F
Endothelial_cells
Embryonic_stem_cells
Epithelial_cells
DC
CMP
Fibroblasts
Gametocytes
Chondrocytes
GMP
B_cell
Hepatocytes
Astrocyte
iPS_cells
Tissue_stem_cells
Macrophage
T_cells
Monocyte
Smooth_muscle_cells
MSC
Osteoblasts
Neurons
NK_cell
Neutrophils
G
Epithelial_cells
Embryonic_stem_cells
CMP
Chondrocytes
Gametocytes
Astrocyte
iPS_cells
T_cells
Neurons
Smooth_muscle_cells
NK_cell

Supplementary Figure S2-2-2-1. Incoming cell–cell communication networks of major immune and epithelial cell types.

CellChat analysis showing incoming signaling interactions received by (A) T cells, (B) B cells, (C) Monocytes, (D) Macrophages, (E) NK cells, (F) CMPs, and (G) Epithelial cells in metastatic prostate cancer. Line thickness represents the relative strength of inferred communication from other cell populations to the indicated receiver cell type.

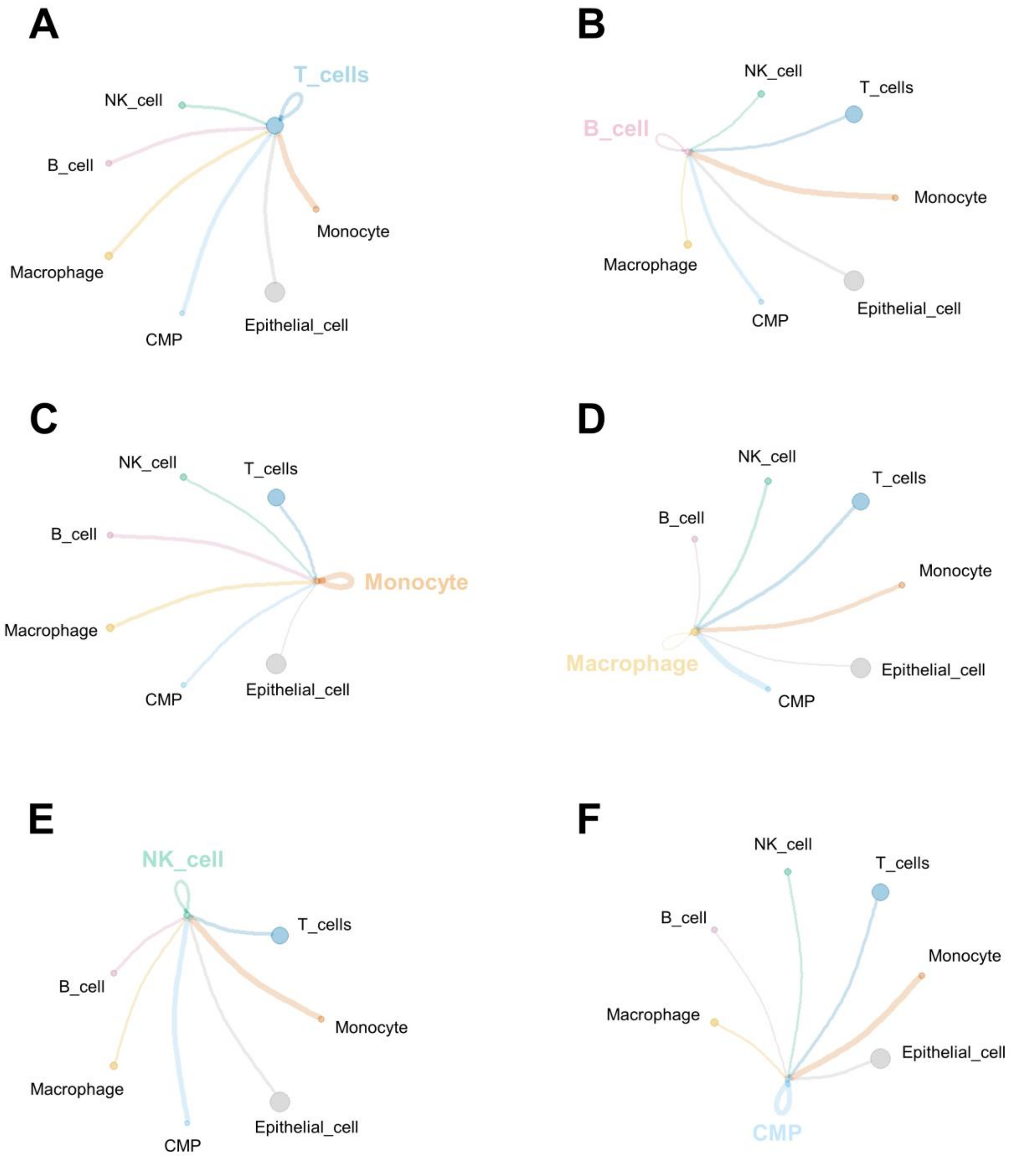

Supplementary Figure S2-2-2-2. Simplified incoming signaling networks among major immune subsets.

Circle plots showing incoming intercellular communication received by six major immune populations—(A) T cells, (B) B cells, (C) Monocytes, (D) Macrophages, (E) NK cells, and (F) CMPs—from other immune and epithelial populations in metastatic prostate cancer. This figure corresponds to Supplementary Figure S2-2-2-1 but is simplified and restricted to the major immune subsets for clearer visualization of dominant signaling inputs. Line thickness represents the relative strength of inferred

incoming communication.

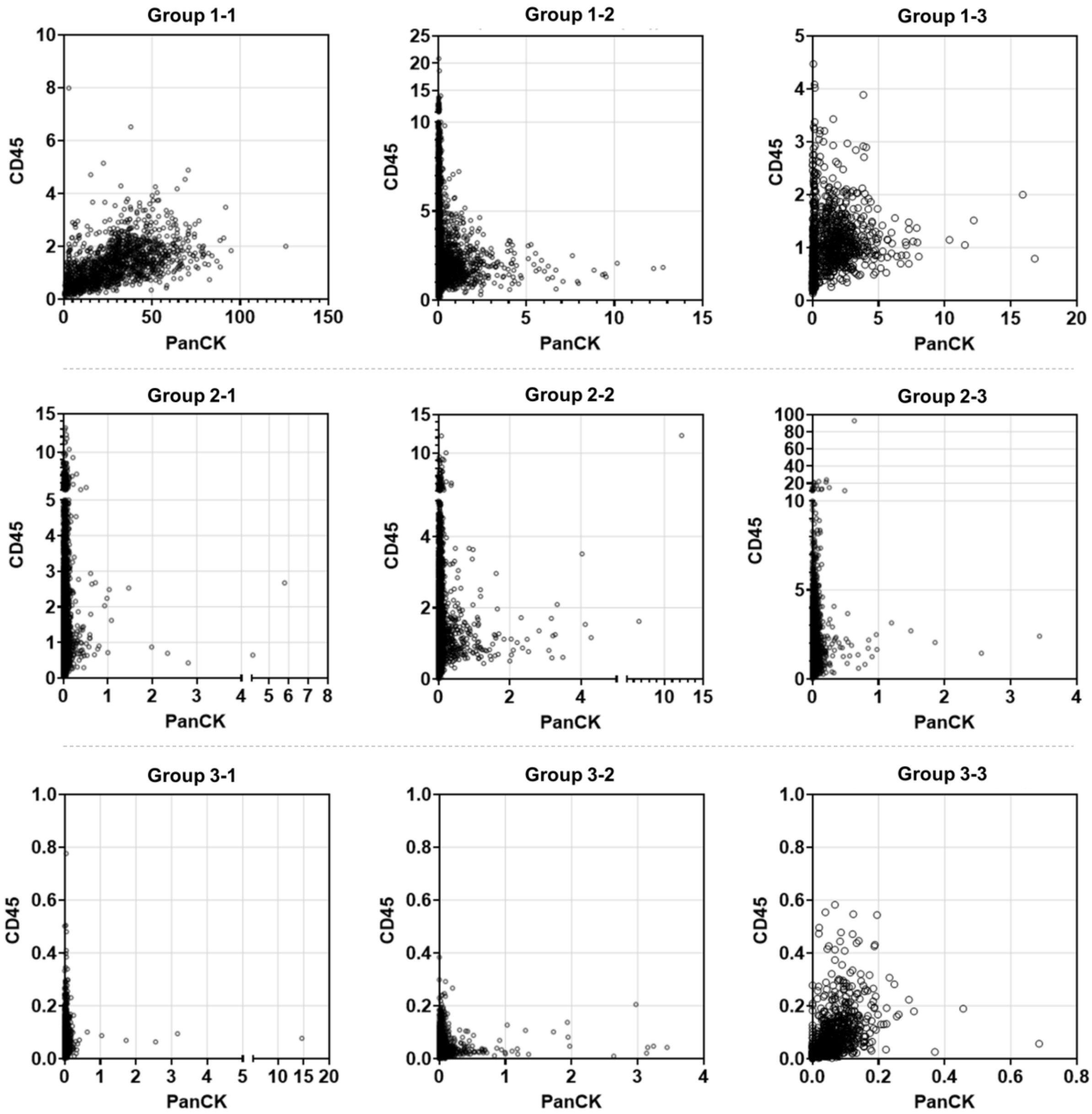

Supplementary Figure S2-3. Scatter plot analysis of CK and CD45 expression in IMC patient cases.

Scatter plots showing the relationship between cytokeratin (CK, x-axis) and CD45 (y-axis) expression intensities in single cells from patient tissue samples analyzed by Imaging Mass Cytometry (IMC). Data are presented for three major groups categorized by pan-CK expression levels (Group 1: CK-high, Group 2: CK-medium, Group 3: CK-low). Within each group, three representative patient cases are displayed (e.g., Group 1–1, 1–2, 1–3). Each point corresponds to an individual cell. These plots provide a direct visualization of CK and CD45 co-expression patterns across different patient samples, supporting the identification of hybrid-like $CK^{+}CD45^{+}$ cells within tumor tissues.

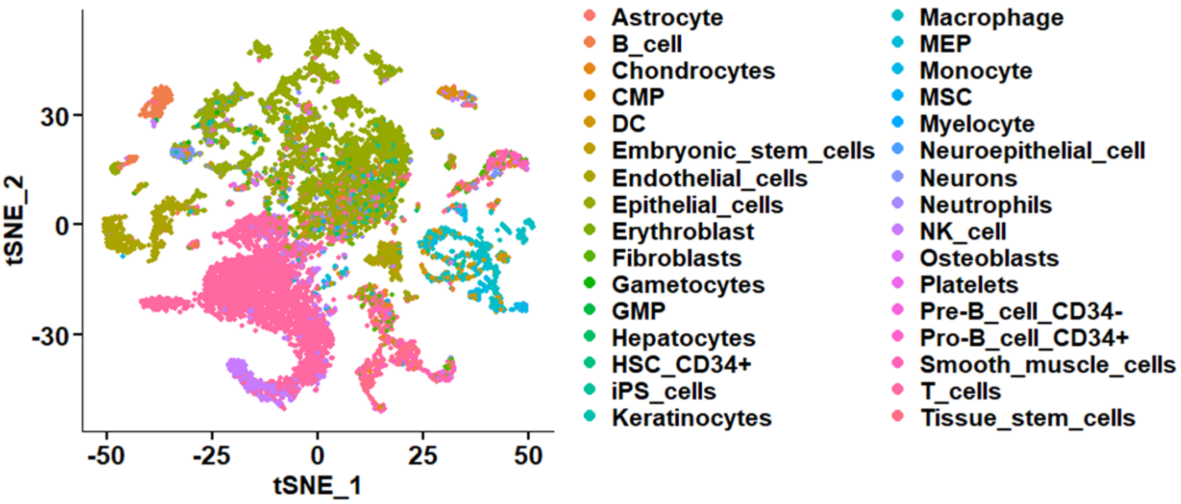

Annotation	Total (Count)	Type				All	
		KP_Pos		Others		KP_Pos	Others
		Count	Percent	Count	Percent	Percent	Percent
T_cells	3534	265	1.88	3269	23.16	1.8774354	23.159759
Epithelial_cells	4399	128	0.91	4271	30.26	0.9068367	30.25859
NK_cell	643	53	0.38	590	4.18	0.3754871	4.1799504
Macrophage	719	45	0.32	674	4.78	0.3188098	4.775062
B_cell	295	25	0.18	270	1.91	0.1771165	1.9128587
Tissue_stem_cells	939	22	0.16	917	6.5	0.1558626	6.4966348
DC	302	20	0.14	282	2	0.1416932	1.9978746
Monocyte	299	17	0.12	282	2	0.1204392	1.9978746
Smooth_muscle_cells	502	8	0.06	494	3.5	0.0566773	3.4998229
Endothelial_cells	1114	7	0.05	1107	7.84	0.0495926	7.8427205
Fibroblasts	248	4	0.03	244	1.73	0.0283386	1.7286575
CMP	102	3	0.02	99	0.7	0.021254	0.7013815
GMP	11	3	0.02	8	0.06	0.021254	0.0566773
Neurons	275	2	0.01	273	1.93	0.0141693	1.9341126
iPS_cells	137	2	0.01	135	0.96	0.0141693	0.9564293
Chondrocytes	173	1	0.01	172	1.22	0.0070847	1.2185618
Embryonic_stem_cells	102	1	0.01	101	0.72	0.0070847	0.7155508
Neutrophils	18	1	0.01	17	0.12	0.0070847	0.1204392
Pre-B_cell_CD34-	7	1	0.01	6	0.04	0.0070847	0.042508
Astrocyte	35	0	0	35	0.25	0	0.2479632
Erythroblast	2	0	0	2	0.01	0	0.0141693
Gametocytes	18	0	0	18	0.13	0	0.1275239
HSC_CD34+	23	0	0	23	0.16	0	0.1629472
Hepatocytes	94	0	0	94	0.67	0	0.6659582
Keratinocytes	5	0	0	5	0.04	0	0.0354233
MEP	5	0	0	5	0.04	0	0.0354233
MSC	11	0	0	11	0.08	0	0.0779313
Myelocyte	1	0	0	1	0.01	0	0.0070847
Neuroepithelial_cell	18	0	0	18	0.13	0	0.1275239
Osteoblasts	80	0	0	80	0.57	0	0.5667729
Platelets	3	0	0	3	0.02	0	0.021254
Pro-B_cell_CD34+	1	0	0	1	0.01	0	0.0070847

Supplementary Figure S3-1. KP_Pos and Other Cell Distribution Based on Reference-Based Cell-Level Annotation (related to Figure 3A-b).

KP_Pos ($KRT18^{+}PTPRC^{+}$) and Other cells were annotated at the single-cell level using a reference-based method (SingleR) as shown in Figure 3A-a. This table summarizes the distribution of KP_Pos

and Other cells across annotated cell types. Immune populations enriched for KP_Pos cells, including B cells, CMP, macrophages, monocytes, NK cells, and T cells, are highlighted in orange.

Annotation	Total (Count)	Type				All	
		KP_Pos		Others		KP_Pos	Others
		Count	Percent	Count	Percent	Percent	Percent
T_cells	3392	230	6.78	3162	93.22	1.629472	22.4017
Epithelial_cells	5692	185	3.25	5507	96.75	1.310662	39.01523
Macrophage	1225	79	6.45	1146	93.55	0.559688	8.119022
NK_cell	492	45	9.15	447	90.85	0.31881	3.166844
B_cell	290	30	10.34	260	89.66	0.21254	1.842012
Tissue_stem_cells	990	20	2.02	970	97.98	0.141693	6.872122
CMP	208	6	2.88	202	97.12	0.042508	1.431102
Monocyte	126	6	4.76	120	95.24	0.042508	0.850159
Endothelial_cells	1092	4	0.37	1088	99.63	0.028339	7.708112
Smooth_muscle_cells	608	3	0.49	605	99.51	0.021254	4.28622

Supplementary Figure S3-2. KP_Pos and Other Cell Distribution Based on Cluster-Level Annotation by Majority Voting (related to Figure 3A-c).

Cell types were assigned to Seurat clusters by majority voting based on reference annotations as shown in Figure 3A-c. This table summarizes the distribution of KP_Pos and Other cells across cluster-assigned cell types. Immune subtypes with high KP_Pos representation are highlighted in orange.

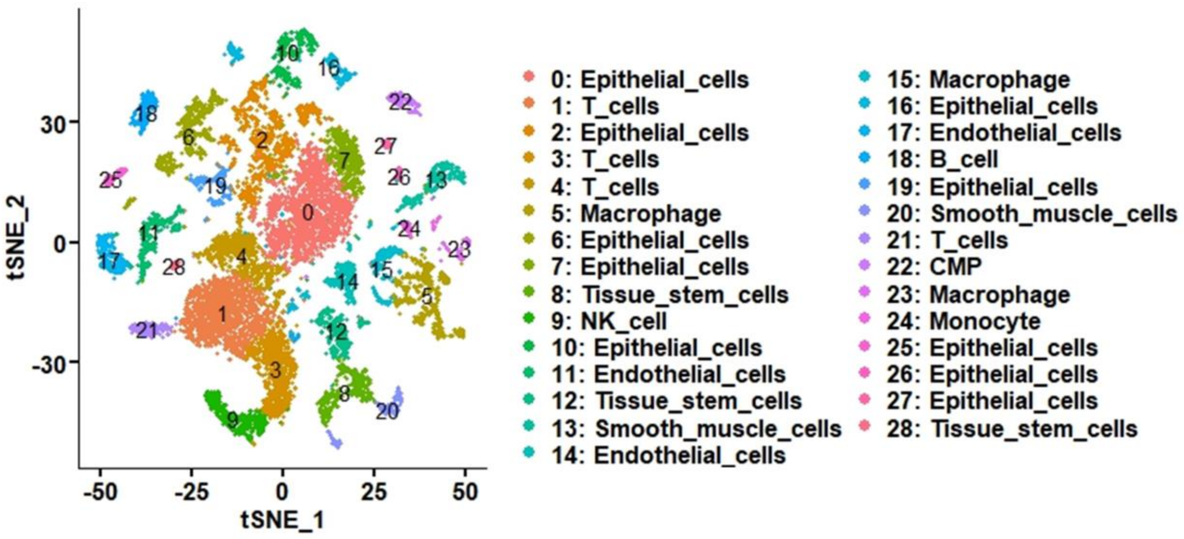

Cluster: Annotation	Total (Count)	Type				All	
		KP_Pos		Others		KP_Pos	Others
		Count	Percent	Count	Percent	Percent	Percent
1: T_cells	1509	114	7.55	1395	92.45	0.807651	9.883103
2: Epithelial_cells	1165	66	5.67	1099	94.33	0.467588	7.786043
3: T_cells	858	58	6.76	800	93.24	0.41091	5.667729
5: Macrophage	743	50	6.73	693	93.27	0.354233	4.909671
4: T_cells	797	46	5.77	751	94.23	0.325894	5.320581
9: NK_cell	492	45	9.15	447	90.85	0.31881	3.166844
10: Epithelial_cells	434	31	7.14	403	92.86	0.219625	2.855119
0: Epithelial_cells	1969	30	1.52	1939	98.48	0.21254	13.73716
18: B_cell	290	30	10.34	260	89.66	0.21254	1.842012
23: Macrophage	138	22	15.94	116	84.06	0.155863	0.821821
6: Epithelial_cells	732	13	1.78	719	98.22	0.092101	5.093872
21: T_cells	228	12	5.26	216	94.74	0.085016	1.530287
26: Epithelial_cells	69	11	15.94	58	84.06	0.077931	0.41091
27: Epithelial_cells	62	10	16.13	52	83.87	0.070847	0.368402
8: Tissue_stem_cells	505	8	1.58	497	98.42	0.056677	3.521077
28: Tissue_stem_cells	57	8	14.04	49	85.96	0.056677	0.347148
7: Epithelial_cells	536	7	1.31	529	98.69	0.049593	3.747786
15: Macrophage	344	7	2.03	337	97.97	0.049593	2.387531
16: Epithelial_cells	334	7	2.1	327	97.9	0.049593	2.316684
19: Epithelial_cells	268	6	2.24	262	97.76	0.042508	1.856181
22: CMP	208	6	2.88	202	97.12	0.042508	1.431102
24: Monocyte	126	6	4.76	120	95.24	0.042508	0.850159
12: Tissue_stem_cells	428	4	0.93	424	99.07	0.028339	3.003897
25: Epithelial_cells	123	4	3.25	119	96.75	0.028339	0.843075
13: Smooth_muscle_cells	376	2	0.53	374	99.47	0.014169	2.649663
17: Endothelial_cells	294	2	0.68	292	99.32	0.014169	2.068721
11: Endothelial_cells	433	1	0.23	432	99.77	0.007085	3.060574
14: Endothelial_cells	365	1	0.27	364	99.73	0.007085	2.578817
20: Smooth_muscle_cells	232	1	0.43	231	99.57	0.007085	1.636557

Supplementary Figure S3-3. Cluster-to-Cell Type Mapping and KP_Pos Distribution.

Seurat clusters were mapped to reference-based cell types.

Top: t-SNE plot showing Seurat clusters labeled with mapped cell type identities.

Bottom: Summary table presenting the number and percentage of KP_Pos (KRT18^{+}PTPRC^{+}) and Other cells across mapped cluster identities.

KP_Pos cells were mainly enriched in B cells, CMP, macrophages, monocytes, NK cells, and T cells,

which are highlighted in orange.

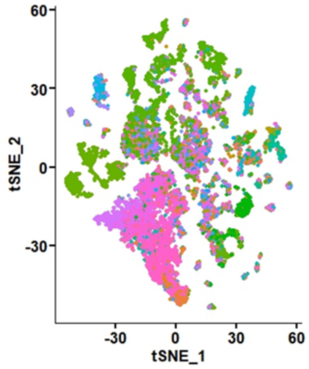

Annotation	Total (Count)	Type				All	
		KP_Pos		Others		KP_Pos	KP_Pos
		Count	Percent	Count	Percent	Percent	Percent
Epithelial cells	3002	98	3.26	2904	96.74	0.694297	20.57386
Tem/Trm cytotoxic T cells	1450	94	6.48	1356	93.52	0.665958	9.606801
Tem/Effector helper T cells	785	58	7.39	727	92.61	0.41091	5.150549
Regulatory T cells	679	39	5.74	640	94.26	0.276302	4.534183
Tcm/Naive helper T cells	1169	31	2.65	1138	97.35	0.219625	8.062345
NK cells	695	27	3.88	668	96.12	0.191286	4.732554
CD16+ NK cells	407	26	6.39	381	93.61	0.184201	2.699256
Memory B cells	475	25	5.26	450	94.74	0.177117	3.188098
Type 1 helper T cells	235	23	9.79	212	90.21	0.162947	1.501948
Alveolar macrophages	603	22	3.65	581	96.35	0.155863	4.116188
Mast cells	268	14	5.22	254	94.78	0.099185	1.799504
Tem/Temra cytotoxic T cells	161	14	8.7	147	91.3	0.099185	1.041445
Macrophages	201	13	6.47	188	93.53	0.092101	1.331916
Type 17 helper T cells	94	13	13.83	81	86.17	0.092101	0.573858
CD16- NK cells	205	9	4.39	196	95.61	0.063762	1.388594
Intestinal macrophages	138	9	6.52	129	93.48	0.063762	0.913921
Naive B cells	128	7	5.47	121	94.53	0.049593	0.857244
Tcm/Naive cytotoxic T cells	86	7	8.14	79	91.86	0.049593	0.559688
Trm cytotoxic T cells	110	7	6.36	103	93.64	0.049593	0.72972
Endothelial cells	1184	6	0.51	1178	99.49	0.042508	8.345731
Follicular helper T cells	46	6	13.04	40	86.96	0.042508	0.283386
Intermediate macrophages	98	6	6.12	92	93.88	0.042508	0.651789
Plasmablasts	305	6	1.97	299	98.03	0.042508	2.118314
B cells	28	5	17.86	23	82.14	0.035423	0.162947
Classical monocytes	163	5	3.07	158	96.93	0.035423	1.119377
Double-positive thymocytes	141	5	3.55	136	96.45	0.035423	0.963514
Mono-mac	44	5	11.36	39	88.64	0.035423	0.276302
Fibroblasts	541	4	0.74	537	99.26	0.028339	3.804463
ILC3	62	4	6.45	58	93.55	0.028339	0.41091
MAIT cells	88	4	4.55	84	95.45	0.028339	0.595112
Non-classical monocytes	48	4	8.33	44	91.67	0.028339	0.311725
DC2	62	2	3.23	60	96.77	0.014169	0.42508
gamma-delta T cells	11	2	18.18	9	81.82	0.014169	0.063762
Treg(diff)	10	2	20	8	80	0.014169	0.056677
CD8a/a	3	1	33.33	2	66.67	0.007085	0.014169
CRTAM+ gamma-delta T cells	36	1	2.78	35	97.22	0.007085	0.247963
DC	27	1	3.7	26	96.3	0.007085	0.184201
Early lymphoid/T lymphoid	6	1	16.67	5	83.33	0.007085	0.035423
Erythrophagocytic macrophages	159	1	0.63	158	99.37	0.007085	1.119377
T(agonist)	17	1	5.88	16	94.12	0.007085	0.113355
Age-associated B cells	24	0	0	24	100	0	0.170032
CD8a/b(entry)	3	0	0	3	100	0	0.021254
DC precursor	3	0	0	3	100	0	0.021254
DC1	6	0	0	6	100	0	0.042508
Double-negative thymocytes	17	0	0	17	100	0	0.120439
Early erythroid	3	0	0	3	100	0	0.021254
ETP	1	0	0	1	100	0	0.007085
Germinal center B cells	1	0	0	1	100	0	0.007085
ILC	1	0	0	1	100	0	0.007085
ILC precursor	2	0	0	2	100	0	0.014169
Kupffer cells	5	0	0	5	100	0	0.035423
Late erythroid	2	0	0	2	100	0	0.014169
Megakaryocyte-erythroid-mast cell progenitor	1	0	0	1	100	0	0.007085
Megakaryocytes/platelets	1	0	0	1	100	0	0.007085
Memory CD4+ cytotoxic T cells	2	0	0	2	100	0	0.014169
MEMP	1	0	0	1	100	0	0.007085
Mid erythroid	1	0	0	1	100	0	0.007085
Migratory DCs	7	0	0	7	100	0	0.049593
Myelocytes	34	0	0	34	100	0	0.240878
NKT cells	6	0	0	6	100	0	0.042508
pDC	5	0	0	5	100	0	0.035423
Pre-pro-B cells	2	0	0	2	100	0	0.014169
Proliferative germinal center B cells	13	0	0	13	100	0	0.092101
Tem/Effector helper T cells PD1+	3	0	0	3	100	0	0.021254
Transitional B cells	1	0	0	1	100	0	0.007085

Supplementary Figure S3-4. Cell Subtype Annotation Using CellTypist and KP_Pos Distribution.

Cells were annotated into detailed epithelial, immune, and stromal subtypes using CellTypist.

Left: t-SNE plot showing annotated cell subtypes.

Right: Summary table presenting the number and percentage of KP_Pos (KRT18^{+}PTPRC^{+}) and Other cells across CellTypist-defined subtypes.

KP_Pos cells were broadly distributed across immune-related subtypes, with enriched populations highlighted in orange.

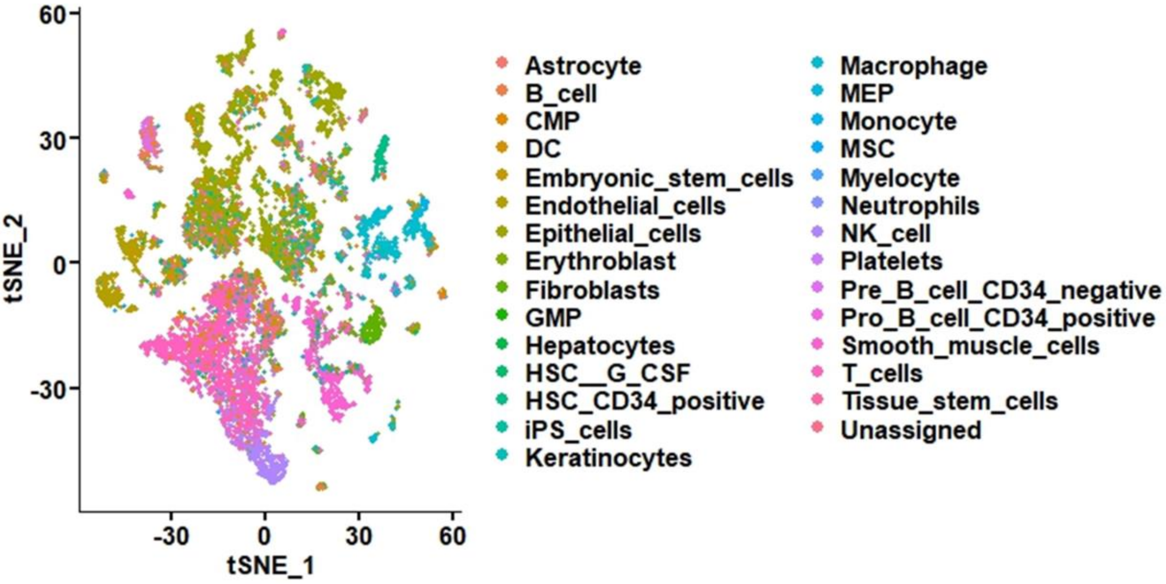

Annotation	Total (Count)	Type				All	
		KP_Pos		Others		KP_Pos	Others
		Count	Percent	Count	Percent	Percent	Percent
Epithelial_cells	3952	197	4.98	3755	95.02	1.395678	26.6029
T_cells	2377	144	6.06	2233	93.94	1.020191	15.82005
Macrophage	938	58	6.18	880	93.82	0.41091	6.234502
NK_cell	713	49	6.87	664	93.13	0.347148	4.704215
Smooth_muscle_cells	1406	41	2.92	1365	97.08	0.290471	9.670563
CMP	720	30	4.17	690	95.83	0.21254	4.888417
Pre_B_cell_CD34_negative	177	19	10.73	158	89.27	0.134609	1.119377
DC	271	16	5.9	255	94.1	0.113355	1.806589
B_cell	151	13	8.61	138	91.39	0.092101	0.977683
HSC_CD34_positive	298	9	3.02	289	96.98	0.063762	2.047467
Keratinocytes	352	7	1.99	345	98.01	0.049593	2.444208
Monocyte	122	6	4.92	116	95.08	0.042508	0.821821
Fibroblasts	506	5	0.99	501	99.01	0.035423	3.549416
Tissue_stem_cells	255	5	1.96	250	98.04	0.035423	1.771165
iPS_cells	372	5	1.34	367	98.66	0.035423	2.600071
Endothelial_cells	689	2	0.29	687	99.71	0.014169	4.867163
HSC__G_CSF	118	2	1.69	116	98.31	0.014169	0.821821
Astrocyte	28	0	0	28	100	0	0.198371
Embryonic_stem_cells	15	0	0	15	100	0	0.10627
Erythroblast	15	0	0	15	100	0	0.10627
GMP	2	0	0	2	100	0	0.014169
Hepatocytes	19	0	0	19	100	0	0.134609
MEP	4	0	0	4	100	0	0.028339
MSC	77	0	0	77	100	0	0.545519
Myelocyte	2	0	0	2	100	0	0.014169
Neutrophils	3	0	0	3	100	0	0.021254
Platelets	5	0	0	5	100	0	0.035423
Pro_B_cell_CD34_positive	5	0	0	5	100	0	0.035423
Unassigned	523	0	0	523	100	0	3.705278

Supplementary Figure S3-5. Marker-Based Annotation and Immune Cell Subtype Distribution of KP_Pos Cells.

KP_Pos ($KRT18^{+}PTPRC^{+}$) and Other cells were annotated using a curated marker-based reference for immune cell subtypes.

Top: t-SNE plot showing cells labeled with marker-defined immune subtypes.

Bottom: Summary table presenting the number and percentage of KP_Pos and Other cells across these subtypes.

KP_Pos cells were enriched in selected immune subtypes, including B cells, CMP, macrophages, monocytes, NK cells, and T cells, which are highlighted in orange.

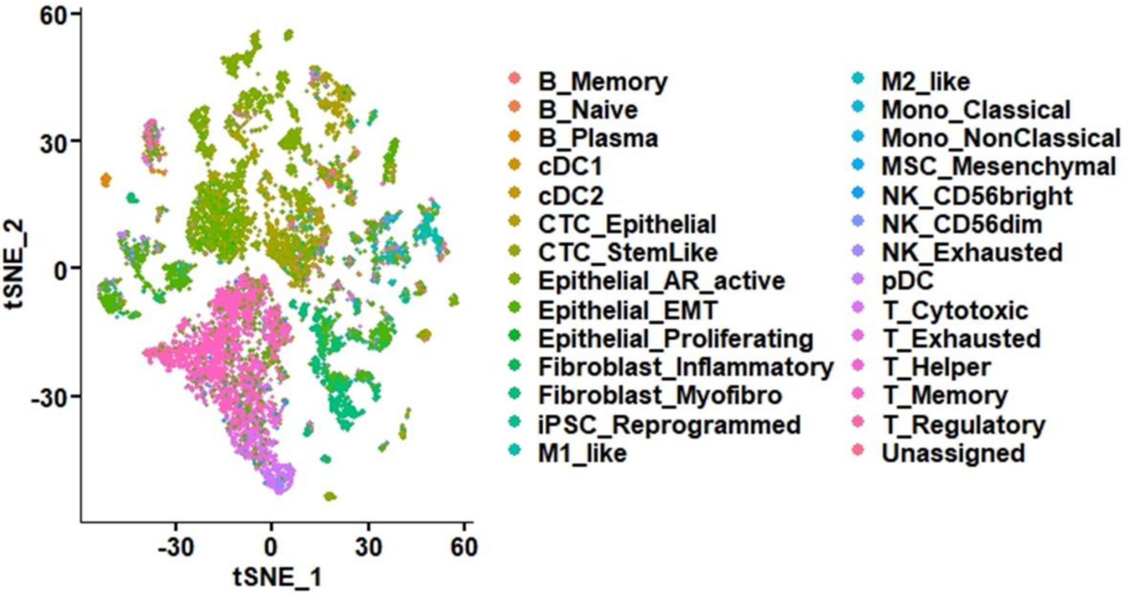

Annotation	Total (Count)	Type				All	
		KP_Pos		Others		KP_Pos	Others
		Count	Percent	Count	Percent	Percent	Percent
T_Memory	2760	246	8.91	2514	91.09	1.742827	17.81084
CTC_Epithelial	1889	97	5.13	1792	94.87	0.687212	12.69571
Epithelial_AR_active	2912	81	2.78	2831	97.22	0.573858	20.05668
Epithelial_EMT	2631	63	2.39	2568	97.61	0.446334	18.19341
T_Cytotoxic	611	34	5.56	577	94.44	0.240878	4.08785
Fibroblast_Myofibro	1212	22	1.82	1190	98.18	0.155863	8.430747
M1_like	387	17	4.39	370	95.61	0.120439	2.621325
Fibroblast_Inflammatory	130	11	8.46	119	91.54	0.077931	0.843075
T_Regulatory	146	6	4.11	140	95.89	0.042508	0.991853
B_Plasma	175	5	2.86	170	97.14	0.035423	1.204392
NK_CD56dim	84	5	5.95	79	94.05	0.035423	0.559688
CTC_StemLike	158	4	2.53	154	97.47	0.028339	1.091038
NK_CD56bright	47	4	8.51	43	91.49	0.028339	0.30464
T_Exhausted	172	4	2.33	168	97.67	0.028339	1.190223
M2_like	75	3	4	72	96	0.021254	0.510096
Mono_NonClassical	36	2	5.56	34	94.44	0.014169	0.240878
B_Memory	26	1	3.85	25	96.15	0.007085	0.177117
Mono_Classical	86	1	1.16	85	98.84	0.007085	0.602196
cDC2	39	1	2.56	38	97.44	0.007085	0.269217
iPSC_Reprogrammed	115	1	0.87	114	99.13	0.007085	0.807651
B_Naive	12	0	0	12	100	0	0.085016
Epithelial_Proliferating	33	0	0	33	100	0	0.233794
MSC_Mesenchymal	56	0	0	56	100	0	0.396741
NK_Exhausted	12	0	0	12	100	0	0.085016
Unassigned	101	0	0	101	100	0	0.715551
cDC1	7	0	0	7	100	0	0.049593
pDC	203	0	0	203	100	0	1.438186

Supplementary Figure S3-6. Subtype-Specific Marker-Based Annotation and Extended Immune Cell Subtype Distribution of KP_Pos Cells.

Cells were annotated into detailed immune subtypes based on subtype-specific marker profiles.

Top: t-SNE plot showing cells labeled with immune subtypes.

Bottom: Summary table presenting the number and percentage of KP_Pos ($KRT18^{+}PTPRC^{+}$) and Other

cells across annotated immune subtypes.

KP_Pos cells were distributed across multiple immune subtypes, with enriched populations highlighted in orange.

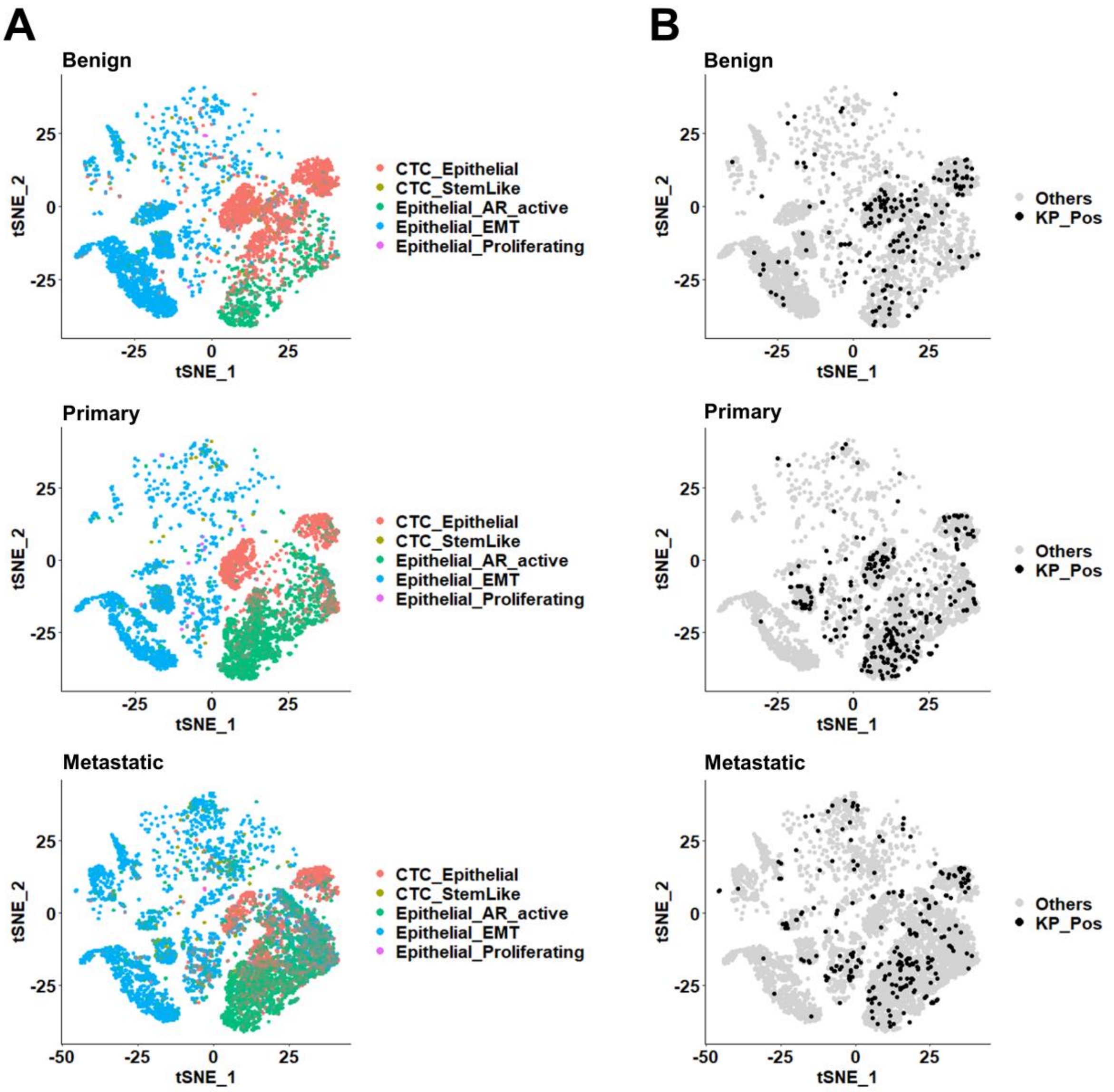

C

KP_Pos_count	Benign	Primary	Metastatic
CTC_Epithelial	124	95	101
CTC_StemLike	0	4	5
Epithelial_AR_active	38	122	80
Epithelial_EMT	28	44	61
Epithelial_Proliferating	0	2	0

KP_Pos_Percent	Benign	Primary	Metastatic
CTC_Epithelial	6.56	7.88	5.27
CTC_StemLike	0.00	8.16	3.50
Epithelial_AR_active	7.45	6.75	2.83
Epithelial_EMT	1.20	3.09	2.33
Epithelial_Proliferating	0.00	10.53	0.00

Supplementary Figure S4-1. Epithelial Cell Subtype Distribution and KP_Pos Cell Composition Across Benign, Primary, and Metastatic Prostate Cancer.

A) Spatial distribution of epithelial cell subtypes in benign (top), primary (middle), and metastatic (bottom) prostate cancer samples. Epithelial cells are categorized into five functional subtypes: CTC_Epithelial, CTC_StemLike, Epithelial_AR_active, Epithelial_EMT, and Epithelial_Proliferating.

B) Spatial distribution of KP_Pos ($KRT18^{+}PTPRC^{+}$) cells versus Others within the epithelial cell compartment across benign, primary, and metastatic conditions. KP_Pos cells are shown in black; Others in gray.

C) Composition of KP_Pos cells within each epithelial subtype across disease states, summarized as counts (left) and percentages (right).

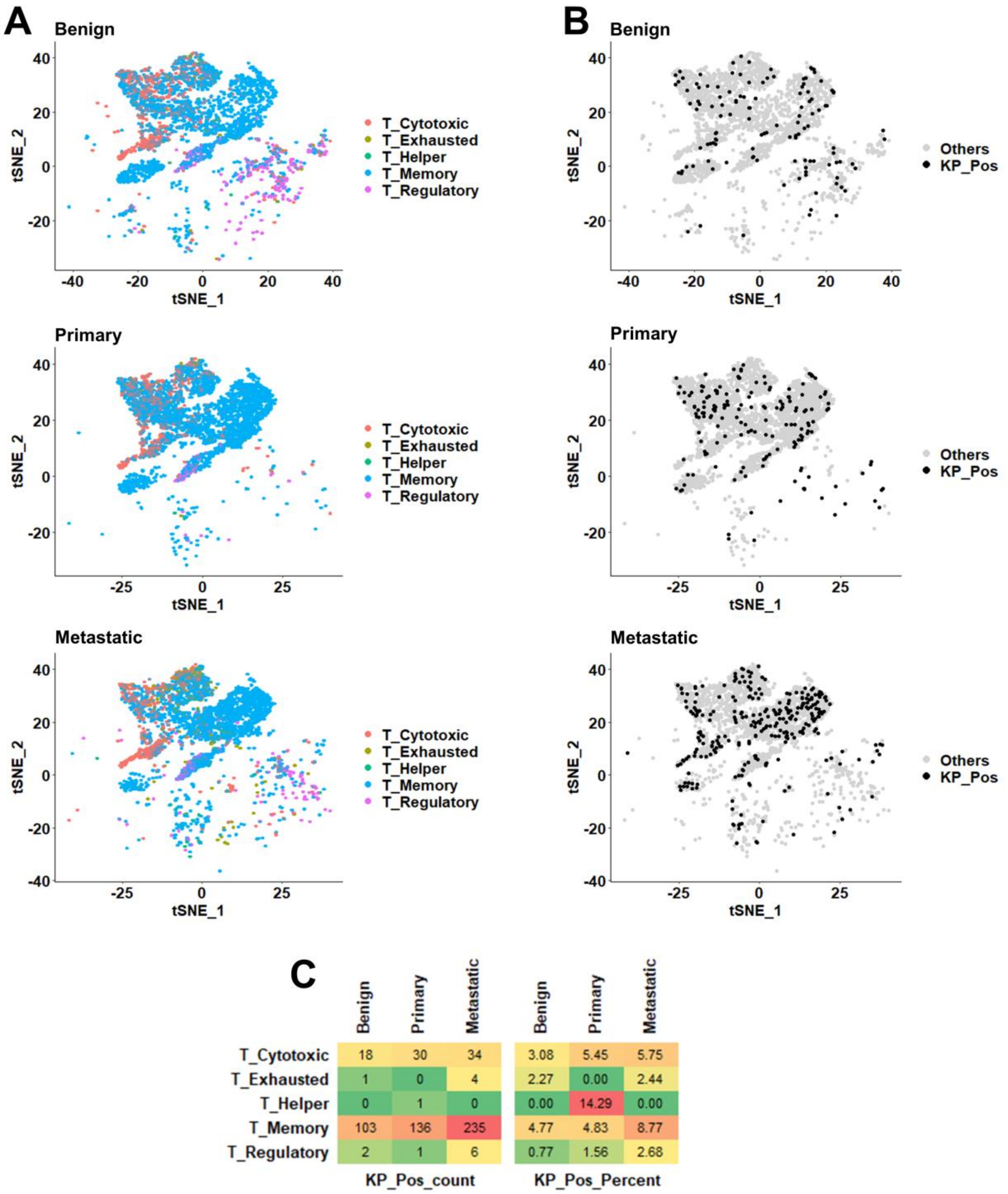

Supplementary Figure S4-2. T Cell Subtype Distribution and KP_Pos Cell Composition Across Benign, Primary, and Metastatic Prostate Cancer.

A) Spatial distribution of T cell subtypes in benign (top), primary (middle), and metastatic (bottom) prostate cancer samples. T cells are classified into five functional subtypes: T_Cytotoxic, T_Exhausted, T_Helper, T_Memory, and T_Regulatory. Each subtype is color-coded and visualized on t-SNE plots.

B) Spatial distribution of KP_Pos (KRT18^{+}PTPRC^{+}) cells versus Others within the T cell compartment across benign, primary, and metastatic conditions. KP_Pos cells are shown in black, while Others are

shown in gray.

C) Summary table showing the composition of KP_Pos cells across T cell subtypes and disease states. Left: KP_Pos cell counts within each T cell subtype. Right: KP_Pos cell percentages relative to each T cell subtype.

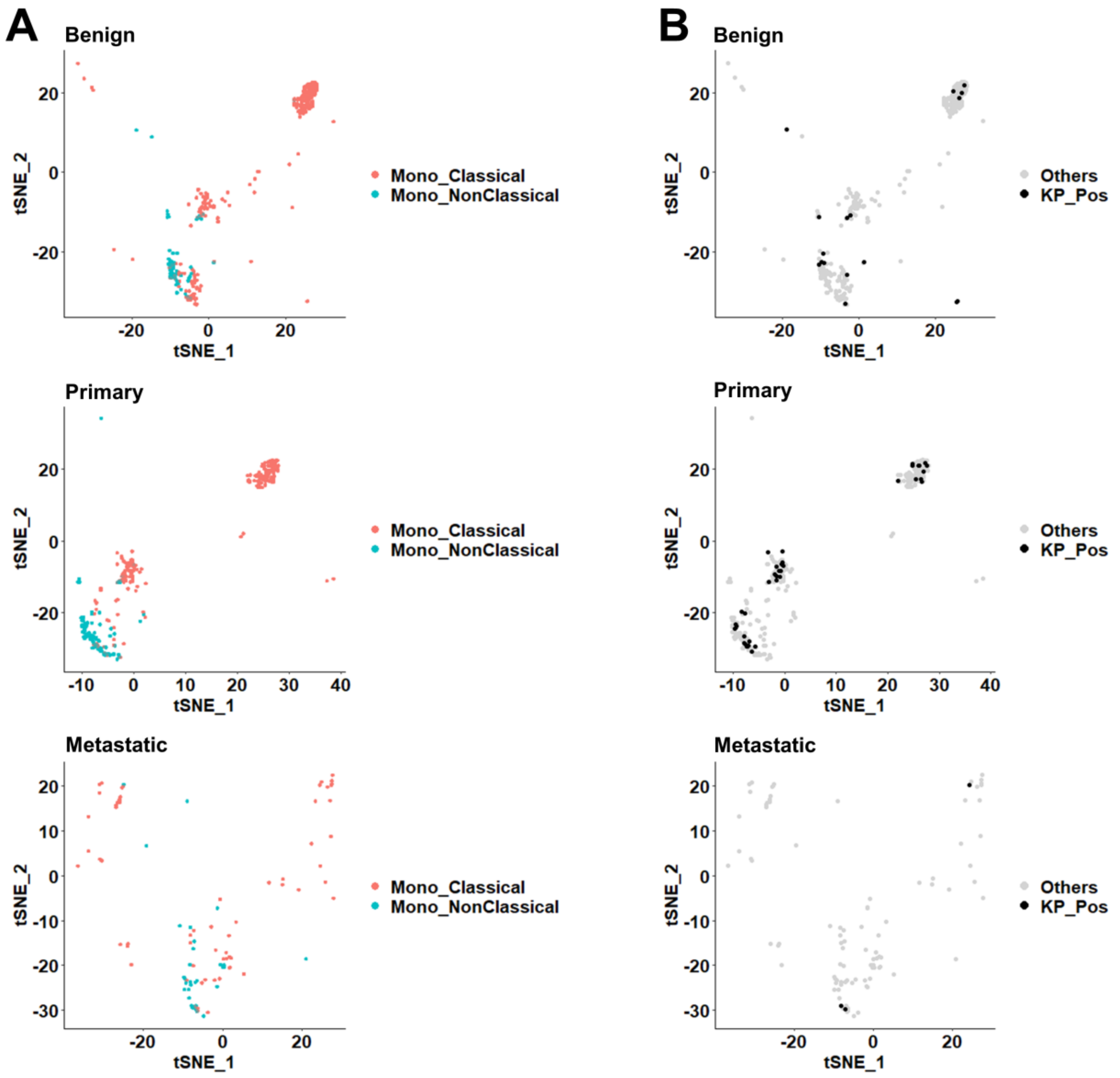

C

	KP_Pos_count: Benign	KP_Pos_count: Primary	KP_Pos_count: Metastatic	KP_Pos_Percent: Benign	KP_Pos_Percent: Primary	KP_Pos_Percent: Metastatic
Mono_Classical	10	26	1	2.98	9.89	1.43
Mono_NonClassical	7	13	2	10.00	12.26	5.88

Supplementary Figure S4-3. Monocyte Subtype Distribution and KP_Pos Cell Composition Across Benign, Primary, and Metastatic Prostate Cancer.

A) Spatial distribution of monocyte subtypes in benign (top), primary (middle), and metastatic (bottom) prostate cancer samples. Monocytes are classified into two subtypes: Mono_Classical and Mono_NonClassical.

B) Spatial distribution of KP_Pos (KRT18^{+}PTPRC^{+}) cells versus Others within the monocyte compartment across benign, primary, and metastatic conditions. KP_Pos cells are shown in black;

Others in gray.

C) Composition of KP_Pos cells within each monocyte subtype across the three disease states, summarized as counts (left) and percentages (right).

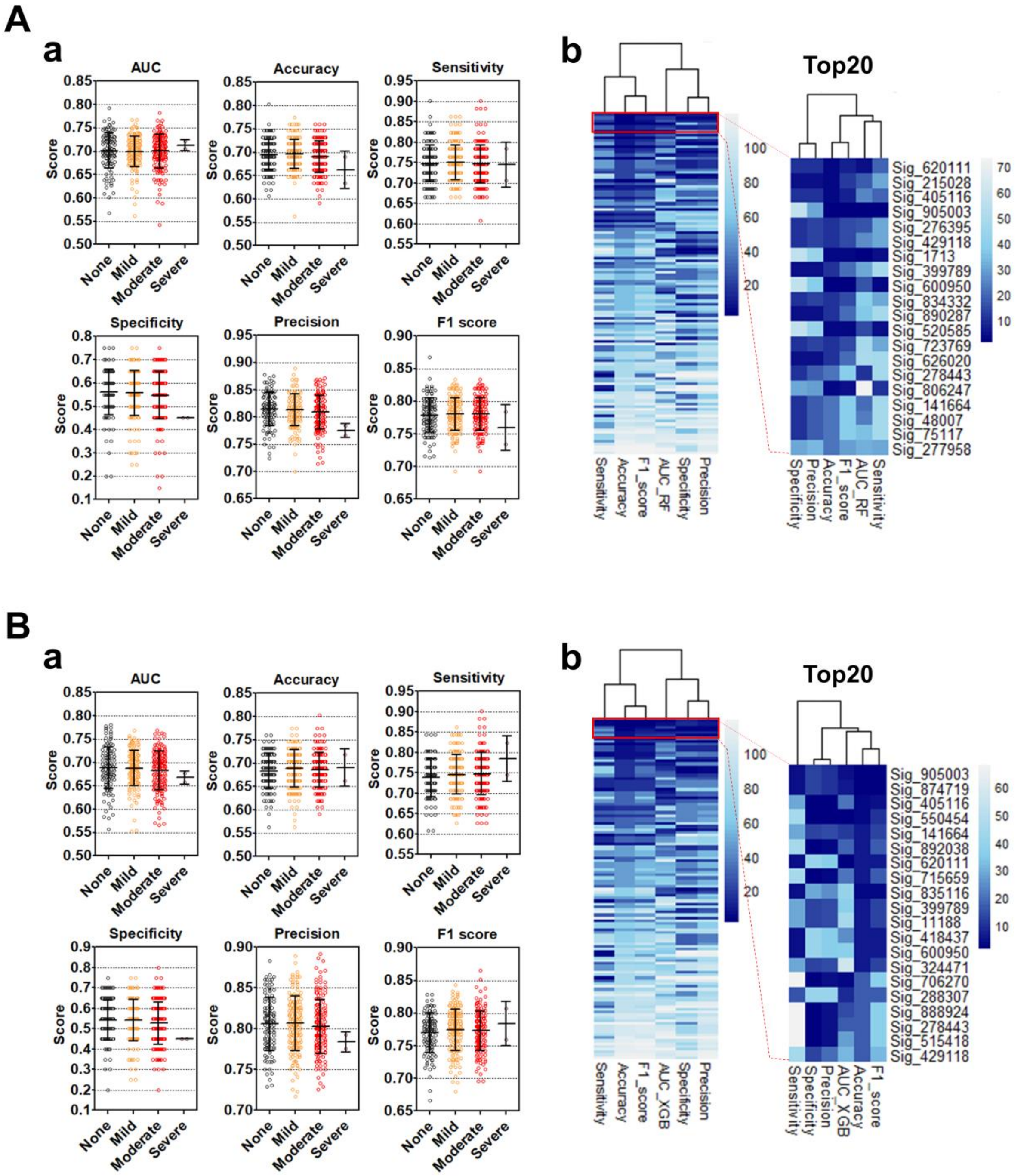

Supplementary Figure S5. Evaluation of Performance Metrics by Overfitting Level and Signature Ranking in RF and XGB Models.

A) Performance Metrics of Random Forest (RF) Models Stratified by Overfitting Level.

(a) Boxplots showing classification performance across four overfitting categories (None, Mild, Moderate, Severe) based on RF analysis. Metrics include AUC, Accuracy, Sensitivity, Specificity, Precision, and F1 score.

(b) Signature-level performance ranking in RF models.

Left: Heatmap representing normalized performance scores across six metrics (AUC, Accuracy, Sensitivity, Specificity, Precision, F1 score) for individual signatures in RF models.
Right: Top 20 ranked signatures based on average performance across metrics in RF models.

B) Performance Metrics of XGBoost (XGB) Models Stratified by Overfitting Level.
(a) Boxplots of performance metrics for XGB models across the same four overfitting levels as in (A), including AUC, Accuracy, Sensitivity, Specificity, Precision, and F1 score.
(b) Signature-level performance ranking in XGB models.
Left: Heatmap of normalized performance scores across metrics for individual signatures in XGB models.
Right: Top 20 ranked signatures based on average performance in XGB models.

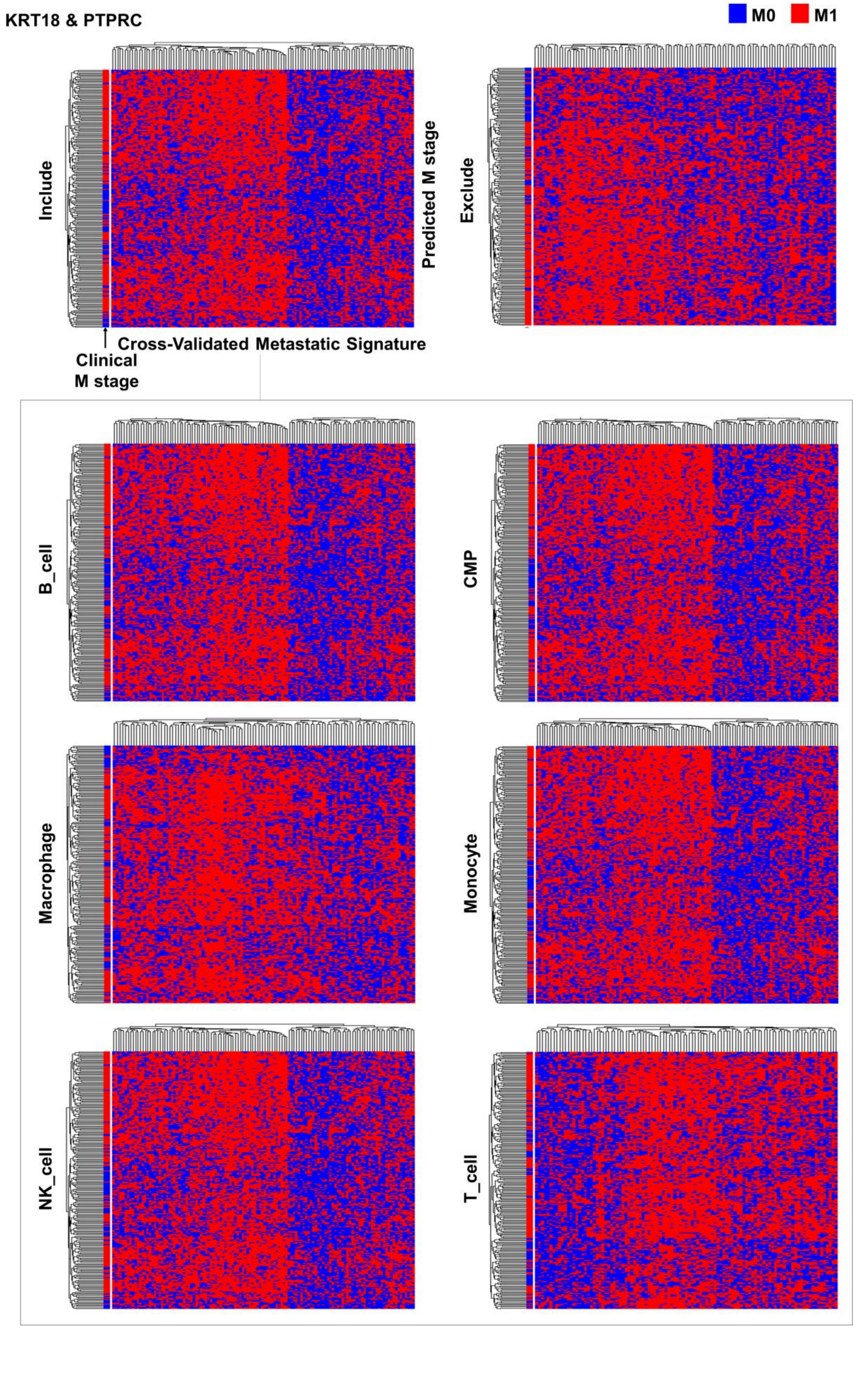

KRT18 & PTPRC
M0
M1
Include
Predicted M stage
Exclude
Clinical
M stage
Cross-Validated Metastatic Signature
B_cell
CMP
Macrophage
Monocyte
NK_cell
T_cell

Supplementary Figure S6-1. Prediction Patterns of M0 and M1 Using Lineage-Specific Gene Signatures.

Heatmaps displaying the M-stage prediction outcomes (M0: blue, M1: red) for bulk RNA-seq samples across representative lineage-specific gene signature subsets. Panels correspond to signatures grouped by cell-of-origin or marker inclusion ($KRT18^{+}$/$PTPRC^{+}$ “Include” vs “Exclude”) and major immune lineages (B cells, CMPs, macrophages, monocytes, NK cells, and T cells).

Samples are consistently aligned across all panels, allowing visualization of prediction consistency and heterogeneity within and between signature groups.

Clustering method		Metastatic only						Metastatic compared to Benign and Primary					
		Cell-level	SingleR majority voting	Cluster-to-cell type mapping	CellTypist	Canonical marker-defined	Subtype-specific marker	Cell-level	SingleR majority voting	Cluster-to-cell type mapping	CellTypist	Canonical marker-defined	Subtype-specific marker
**Cell type**	**Subtype**	Number of Upregulated gene						Number of Upregulated gene					
B cell		3	6	3	0	2	0	6	15	91	0	2	0
	Memory	0	0	0	10	0	0	0	0	0	4	0	0
	Naïve	0	0	0	19	0	0	0	0	0	0	0	0
	Plasma	0	0	0	293	0	438	0	0	0	287	0	374
	Pre B / CD34-	0	0	0	0	6	0	0	0	0	0	0	0
CMP		113	95	113	0	12	0	84	86	106	0	0	0
Macrophage	Alveolar	0	0	0	0	0	0	0	0	0	60	0	0
	Intestinal	0	0	0	10	0	0	0	0	0	12	0	0
	M1-like	0	0	0	0	0	92	0	0	0	0	0	51
	M2-like	0	0	0	0	0	0	0	0	0	0	0	108
		46	44	113	16	35	0	26	35	21	1	18	0
	Intermediate	0	0	0	11	0	0	0	0	0	0	0	0
Monocyte	Classical	0	0	0	208	0	0	0	0	0	0	0	0
		16	9	16	0	24	0	12	91	19	0	20	0
NK cell	CD16	0	0	0	0	0	0	0	0	0	4	0	0
	CD16-	0	0	0	21	0	0	0	0	0	22	0	0
	CD16+	0	0	0	3	0	0	0	0	0	4	0	0
	CD56bright	0	0	0	0	0	2	0	0	0	0	0	2
	CD56dim	0	0	0	0	0	19	0	0	0	0	0	28
		7	2	7	19	1	0	6	18	4	51	1	0
T cell		8	43	49	0	2	0	47	40	24	0	53	0
	Cytotoxic	0	0	0	0	0	7	0	0	0	0	0	6
	Exhausted	0	0	0	0	0	52	0	0	0	0	0	50
	Memory	0	0	0	0	0	11	0	0	0	0	0	11
	Regulatory	0	0	0	10	0	6	0	0	0	35	0	86
	Naïve helper, Central memory helper	0	0	0	0	0	0	0	0	0	138	0	0
	Effector memory	0	0	0	0	0	0	0	0	0	1	0	0
	Effector memory, Effector memory RA, Cytotoxic	0	0	0	0	0	0	0	0	0	2	0	0
	Effector memory, Tissue-resident memory, Cytotoxic	0	0	0	0	0	0	0	0	0	3	0	0
	Type 1 helper	0	0	0	0	0	0	0	0	0	8	0	0
Epithelial cell	CTC	0	0	0	0	0	0	0	0	0	0	0	96
	AR active	0	0	0	0	0	136	0	0	0	390	0	156
		137	53	1061	185	219	0	52	166	343	0	241	0
	EMT	0	0	0	0	0	91	0	0	0	171	0	93

Supplementary Figure S6-2. Comparative analysis of upregulated genes across cell subtypes using multiple annotation strategies.

Table summarizing the number of upregulated genes identified in metastatic prostate cancer cells, either within metastatic cases alone (blue-shaded section on the left) or in metastatic samples compared with benign and primary prostate tissues (orange-shaded section on the right). Results are organized by cell type and subtype (rows), including B cells, CMP, macrophages, monocytes, NK cells, T cells, and epithelial subsets (CTCs, AR-active, EMT). Each column corresponds to a distinct clustering or annotation method: cell-level analysis, SingleR majority voting, cluster-to-cell type mapping, CellTypist annotation, canonical marker-defined assignment, and subtype-specific marker mapping. Numbers indicate the total count of upregulated genes per cell subtype under each method. This comparison highlights both overlapping and method-specific transcriptional programs associated with metastatic progression.

A

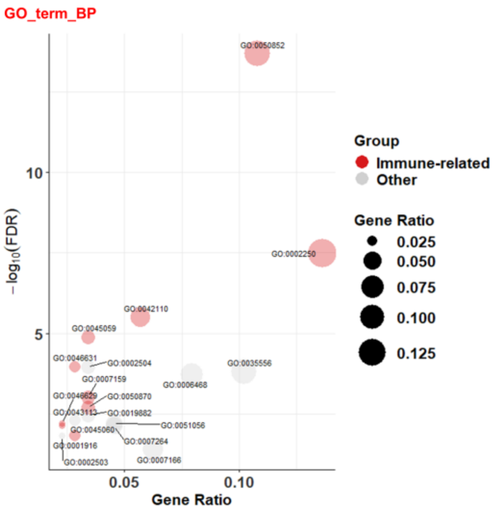

GO ID	GO term name
GO:0050852	T cell receptor signaling pathway
GO:0002250	adaptive immune response
GO:0042110	T cell activation
GO:0045059	positive thymic T cell selection
GO:0046631	alpha-beta T cell activation
GO:0002504	antigen processing and presentation of peptide or polysaccharide antigen via MHC class II
GO:0035556	intracellular signal transduction
GO:0006468	protein phosphorylation
GO:0007159	leukocyte cell-cell adhesion
GO:0050870	positive regulation of T cell activation
GO:0019882	antigen processing and presentation
GO:0043113	receptor clustering
GO:0051056	regulation of small GTPase mediated signal transduction
GO:0046629	gamma-delta T cell activation
GO:0045060	negative thymic T cell selection
GO:0007264	small GTPase-mediated signal transduction
GO:0001916	positive regulation of T cell mediated cytotoxicity
GO:0002503	peptide antigen assembly with MHC class II protein complex
GO:0007166	cell surface receptor signaling pathway

B

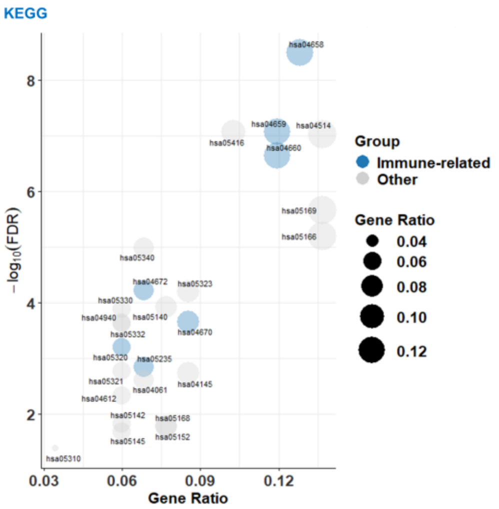

Pathway ID	Pathway name
hsa04658	Th1 and Th2 cell differentiation
hsa04659	Th17 cell differentiation
hsa05416	Viral myocarditis
hsa04514	Cell adhesion molecules
hsa04660	T cell receptor signaling pathway
hsa05169	Epstein-Barr virus infection
hsa05166	Human T-cell leukemia virus 1 infection
hsa05340	Primary immunodeficiency
hsa04672	Intestinal immune network for IgA production
hsa05323	Rheumatoid arthritis
hsa05140	Leishmaniasis
hsa05330	Allograft rejection
hsa04940	Type I diabetes mellitus
hsa04670	Leukocyte transendothelial migration
hsa05332	Graft-versus-host disease
hsa05320	Autoimmune thyroid disease
hsa05235	PD-L1 expression and PD-1 checkpoint pathway in cancer
hsa05321	Inflammatory bowel disease
hsa04145	Phagosome
hsa04061	Viral protein interaction with cytokine and cytokine receptor
hsa04612	Antigen processing and presentation
hsa05142	Chagas disease
hsa05152	Tuberculosis
hsa05168	Herpes simplex virus 1 infection
hsa05145	Toxoplasmosis
hsa05310	Asthma

Supplementary Figure S6-3-1. GO and KEGG enrichment analysis of epithelial cells (clustering: canonical marker-defined).

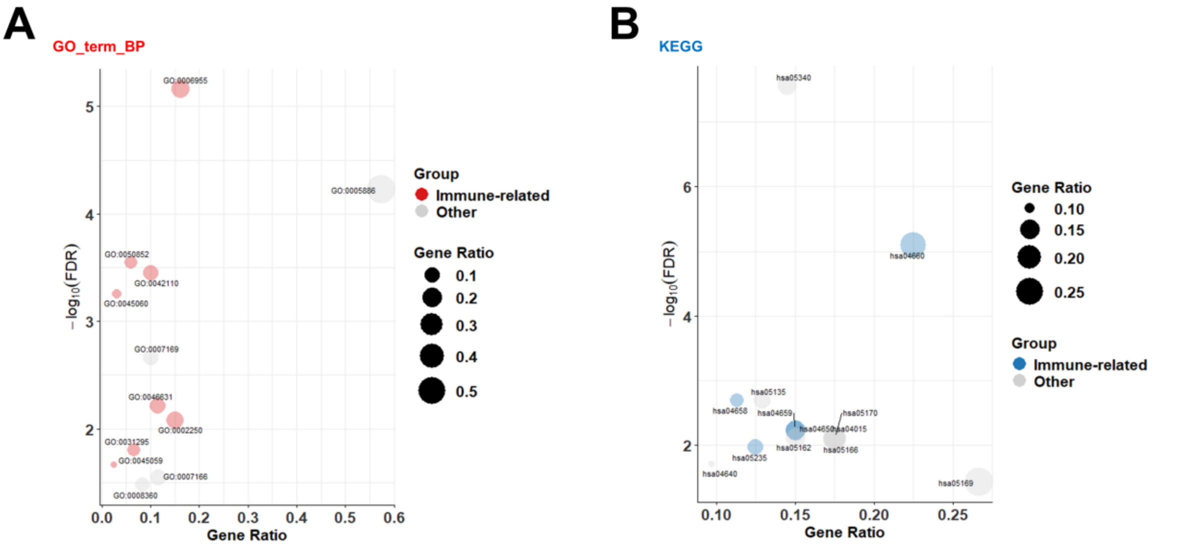

GO ID	GO term name
GO:0008360	regulation of cell shape
GO:0007166	cell surface receptor signaling pathway
GO:0045059	positive thymic T cell selection
GO:0031295	T cell costimulation
GO:0002250	adaptive immune response
GO:0046631	alpha-beta T cell activation
GO:0007169	cell surface receptor protein tyrosine kinase signaling pathway
GO:0045060	negative thymic T cell selection
GO:0042110	T cell activation
GO:0050852	T cell receptor signaling pathway
GO:0005886	plasma membrane
GO:0006955	immune response

Pathway ID	Pathway name
hsa05340	Primary immunodeficiency
hsa04660	T cell receptor signaling pathway
hsa04658	Th1 and Th2 cell differentiation
hsa05135	Yersinia infection
hsa04659	Th17 cell differentiation
hsa04650	Natural killer cell mediated cytotoxicity
hsa05170	Human immunodeficiency virus 1 infection
hsa05162	Measles
hsa04015	Rap1 signaling pathway
hsa05166	Human T-cell leukemia virus 1 infection
hsa05235	PD-L1 expression and PD-1 checkpoint pathway in cancer
hsa04640	Hematopoietic cell lineage
hsa05169	Epstein-Barr virus infection

Supplementary Figure S6-3-2. GO and KEGG enrichment analysis of epithelial cells (clustering: cell-level).

A

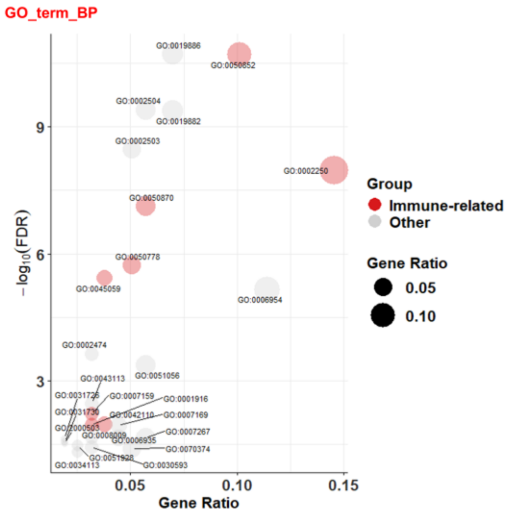

GO ID	GO term name
GO:0019886	antigen processing and presentation of exogenous peptide antigen via MHC class II
GO:0050852	T cell receptor signaling pathway
GO:0002504	antigen processing and presentation of peptide or polysaccharide antigen via MHC class II
GO:0019882	antigen processing and presentation
GO:0002503	peptide antigen assembly with MHC class II protein complex
GO:0002250	adaptive immune response
GO:0050870	positive regulation of T cell activation
GO:0050778	positive regulation of immune response
GO:0045059	positive thymic T cell selection
GO:0006954	inflammatory response
GO:0002474	antigen processing and presentation of peptide antigen via MHC class I
GO:0051056	regulation of small GTPase mediated signal transduction
GO:0043113	receptor clustering
GO:0007159	leukocyte cell-cell adhesion
GO:0001916	positive regulation of T cell mediated cytotoxicity
GO:0042110	T cell activation
GO:0007169	cell surface receptor protein tyrosine kinase signaling pathway
GO:0008009	chemokine activity
GO:0007267	cell-cell signaling
GO:0006935	chemotaxis
GO:0031726	CCR1 chemokine receptor binding
GO:0031730	CCR5 chemokine receptor binding
GO:2000503	positive regulation of natural killer cell chemotaxis
GO:0051928	positive regulation of calcium ion transport
GO:0030593	neutrophil chemotaxis
GO:0070374	positive regulation of ERK1 and ERK2 cascade
GO:0034113	heterotypic cell-cell adhesion

B

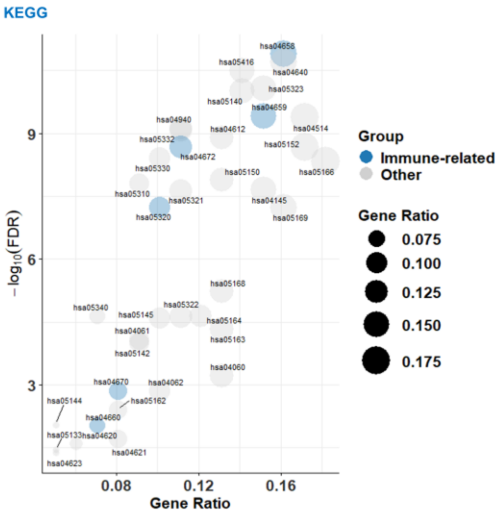

Pathway ID	Pathway name
hsa04658	Th1 and Th2 cell differentiation
hsa04640	Hematopoietic cell lineage
hsa05416	Viral myocarditis
hsa05323	Rheumatoid arthritis
hsa05140	Leishmaniasis
hsa04659	Th17 cell differentiation
hsa04514	Cell adhesion molecules
hsa04940	Type I diabetes mellitus
hsa05332	Graft-versus-host disease
hsa04612	Antigen processing and presentation
hsa04672	Intestinal immune network for IgA production
hsa05152	Tuberculosis
hsa05330	Allograft rejection
hsa05166	Human T-cell leukemia virus 1 infection
hsa05150	Staphylococcus aureus infection
hsa05310	Asthma
hsa04145	Phagosome
hsa05321	Inflammatory bowel disease
hsa05320	Autoimmune thyroid disease
hsa05169	Epstein-Barr virus infection
hsa05168	Herpes simplex virus 1 infection
hsa05164	Influenza A
hsa05340	Primary immunodeficiency
hsa05322	Systemic lupus erythematosus
hsa05145	Toxoplasmosis
hsa05163	Human cytomegalovirus infection
hsa04061	Viral protein interaction with cytokine and cytokine receptor
hsa05142	Chagas disease
hsa04060	Cytokine-cytokine receptor interaction
hsa04062	Chemokine signaling pathway
hsa04670	Leukocyte transendothelial migration
hsa05162	Measles
hsa05144	Malaria
hsa04660	T cell receptor signaling pathway
hsa04621	NOD-like receptor signaling pathway
hsa04620	Toll-like receptor signaling pathway
hsa05133	Pertussis
hsa04623	Cytosolic DNA-sensing pathway

Supplementary Figure S6-3-3. GO and KEGG enrichment analysis of epithelial cells (clustering: CellTypist).

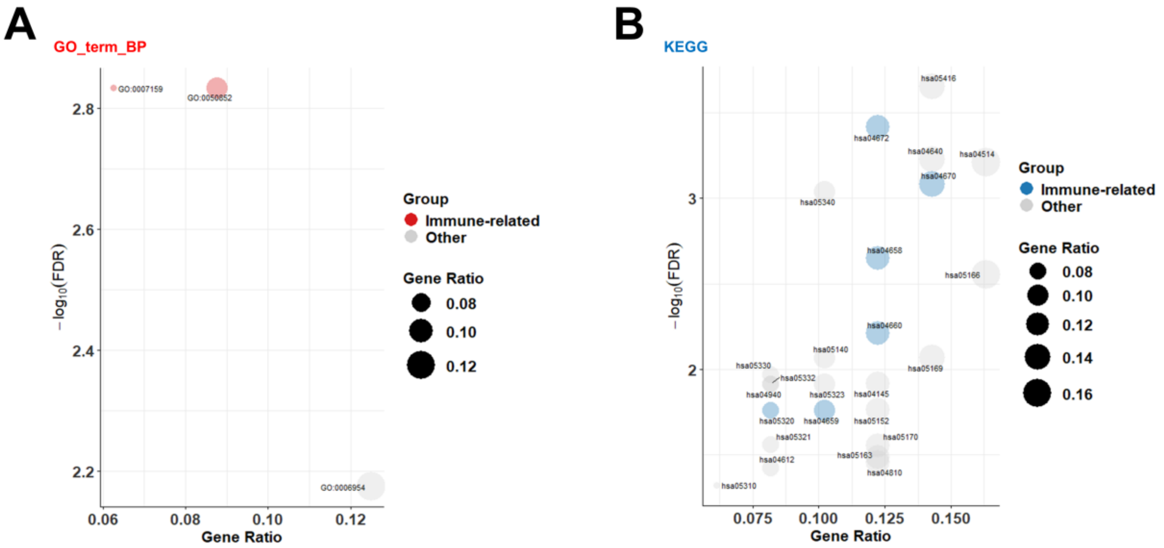

GO ID	GO term name
GO:0007159	leukocyte cell-cell adhesion
GO:0050852	T cell receptor signaling pathway
GO:0006954	inflammatory response

Pathway ID	Pathway name
hsa05416	Viral myocarditis
hsa04672	Intestinal immune network for IgA production
hsa04640	Hematopoietic cell lineage
hsa04514	Cell adhesion molecules
hsa04670	Leukocyte transendothelial migration
hsa05340	Primary immunodeficiency
hsa04658	Th1 and Th2 cell differentiation
hsa05166	Human T-cell leukemia virus 1 infection
hsa04660	T cell receptor signaling pathway
hsa05169	Epstein-Barr virus infection
hsa05140	Leishmaniasis
hsa05330	Allograft rejection
hsa04940	Type I diabetes mellitus
hsa04145	Phagosome
hsa05323	Rheumatoid arthritis
hsa05332	Graft-versus-host disease
hsa04659	Th17 cell differentiation
hsa05320	Autoimmune thyroid disease
hsa05152	Tuberculosis
hsa05321	Inflammatory bowel disease
hsa05170	Human immunodeficiency virus 1 infection
hsa05163	Human cytomegalovirus infection
hsa04810	Regulation of actin cytoskeleton
hsa04612	Antigen processing and presentation
hsa05310	Asthma

Supplementary Figure S6-3-4. GO and KEGG enrichment analysis of epithelial cells (clustering: cell-to-cell type mapping).

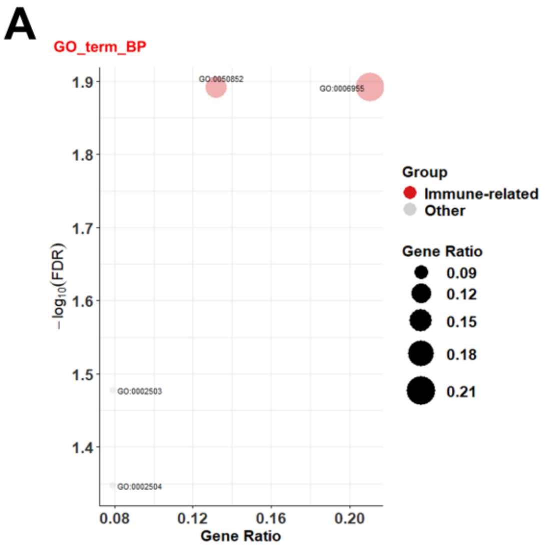

GO ID	GO term name
GO:0050852	T cell receptor signaling pathway
GO:0006955	immune response
GO:0002503	peptide antigen assembly with MHC class II protein complex
GO:0002504	antigen processing and presentation of peptide or polysaccharide antigen via MHC class II

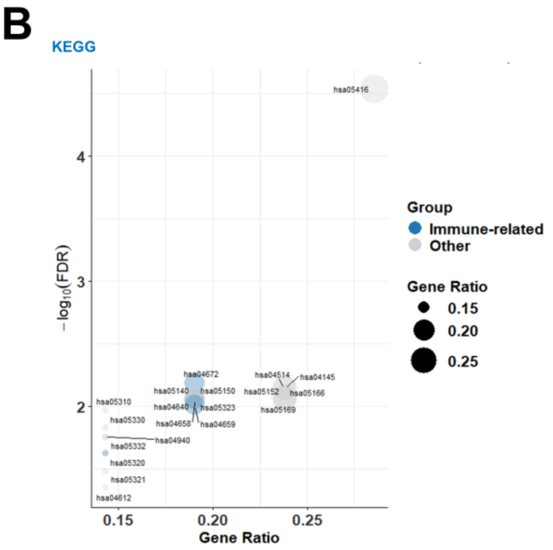

Pathway ID	Pathway name
hsa05416	Viral myocarditis
hsa04672	Intestinal immune network for IgA production
hsa04514	Cell adhesion molecules
hsa04145	Phagosome
hsa05152	Tuberculosis
hsa05140	Leishmaniasis
hsa05169	Epstein-Barr virus infection
hsa04658	Th1 and Th2 cell differentiation
hsa05323	Rheumatoid arthritis
hsa04640	Hematopoietic cell lineage
hsa05166	Human T-cell leukemia virus 1 infection
hsa05150	Staphylococcus aureus infection
hsa04659	Th17 cell differentiation
hsa05310	Asthma
hsa05330	Allograft rejection
hsa04940	Type I diabetes mellitus
hsa05332	Graft-versus-host disease
hsa05320	Autoimmune thyroid disease
hsa05321	Inflammatory bowel disease
hsa04612	Antigen processing and presentation

Supplementary Figure S6-3-5. GO and KEGG enrichment analysis of epithelial cells (clustering: SingleR majority voting).

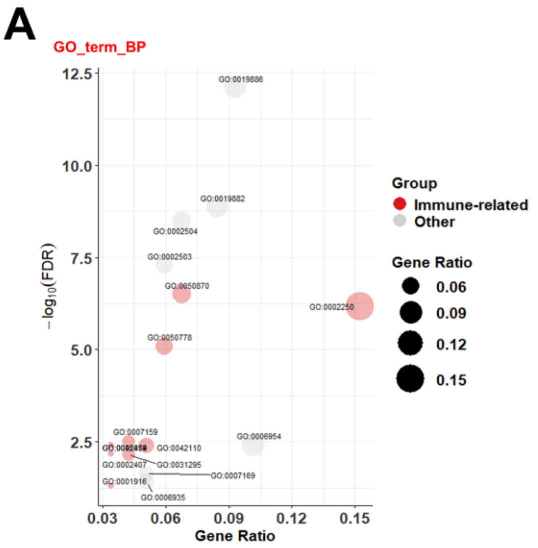

GO ID	GO term name
GO:0019886	antigen processing and presentation of exogenous peptide antigen via MHC cla ss II
GO:0019882	antigen processing and presentation
GO:0002504	antigen processing and presentation of peptide or polysaccharide antigen via M HC class II
GO:0002503	peptide antigen assembly with MHC class II protein complex
GO:0050870	positive regulation of T cell activation
GO:0002250	adaptive immune response
GO:0050778	positive regulation of immune response
GO:0007159	leukocyte cell-cell adhesion
GO:0002474	antigen processing and presentation of peptide antigen via MHC class I
GO:0045059	positive thymic T cell selection
GO:0006954	inflammatory response
GO:0042110	T cell activation
GO:0002407	dendritic cell chemotaxis
GO:0031295	T cell costimulation
GO:0007169	cell surface receptor protein tyrosine kinase signaling pathway
GO:0006935	chemotaxis
GO:0001916	positive regulation of T cell mediated cytotoxicity

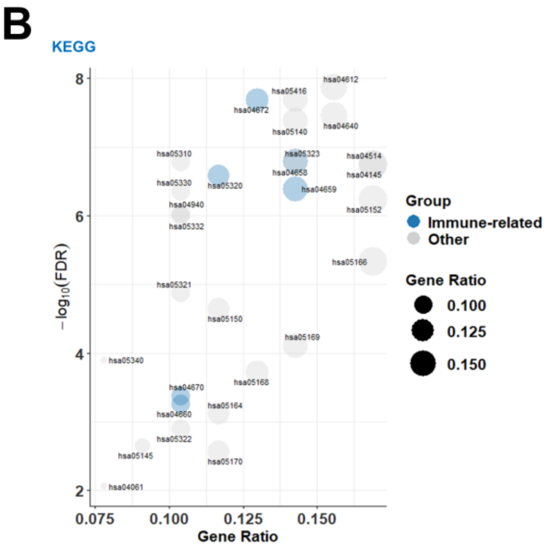

Pathway ID	Pathway name
hsa04612	Antigen processing and presentation
hsa04672	Intestinal immune network for IgA production
hsa05416	Viral myocarditis
hsa04640	Hematopoietic cell lineage
hsa05140	Leishmaniasis
hsa04658	Th1 and Th2 cell differentiation
hsa05323	Rheumatoid arthritis
hsa05310	Asthma
hsa04514	Cell adhesion molecules
hsa04145	Phagosome
hsa05320	Autoimmune thyroid disease
hsa04659	Th17 cell differentiation
hsa05330	Allograft rejection
hsa05152	Tuberculosis
hsa04940	Type I diabetes mellitus
hsa05332	Graft-versus-host disease
hsa05166	Human T-cell leukemia virus 1 infection
hsa05321	Inflammatory bowel disease
hsa05150	Staphylococcus aureus infection
hsa05169	Epstein-Barr virus infection
hsa05340	Primary immunodeficiency
hsa05168	Herpes simplex virus 1 infection
hsa04670	Leukocyte transendothelial migration
hsa04660	T cell receptor signaling pathway
hsa05164	Influenza A
hsa05322	Systemic lupus erythematosus
hsa05145	Toxoplasmosis
hsa05170	Human immunodeficiency virus 1 infection
hsa04061	Viral protein interaction with cytokine and cytokine receptor

Supplementary Figure S6-3-6. GO and KEGG enrichment analysis of epithelial cells (clustering: subtype-specific marker, AR-active subtype).

A

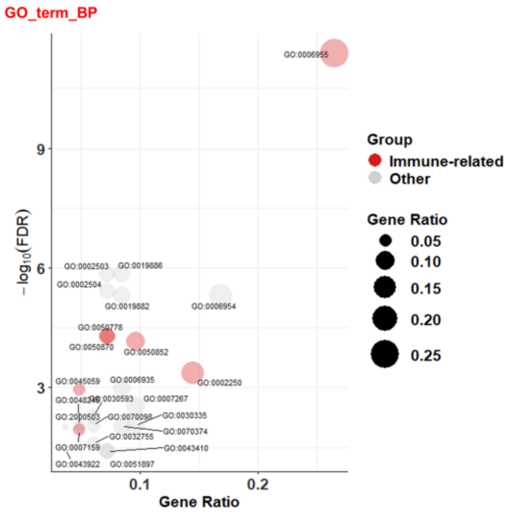

B

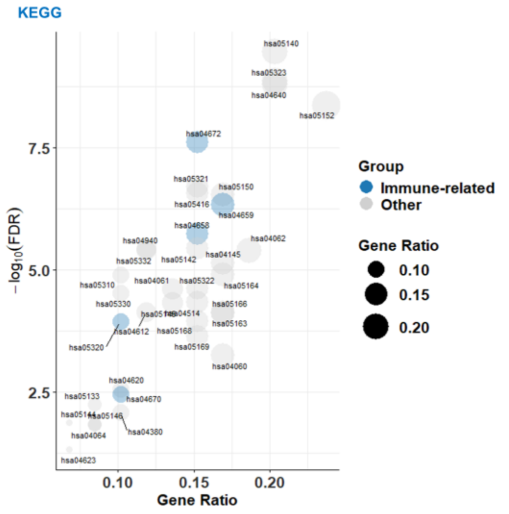

GO ID	GO term name
GO:0006955	immune response
GO:0019886	antigen processing and presentation of exogenous peptide antigen via MHC class II
GO:0002503	peptide antigen assembly with MHC class II protein complex
GO:0002504	antigen processing and presentation of peptide or polysaccharide antigen via MHC class II
GO:0019882	antigen processing and presentation
GO:0006954	inflammatory response
GO:0050778	positive regulation of immune response
GO:0050870	positive regulation of T cell activation
GO:0050852	T cell receptor signaling pathway
GO:0002250	adaptive immune response
GO:0006935	chemotaxis
GO:0045059	positive thymic T cell selection
GO:0007267	cell-cell signaling
GO:0030593	neutrophil chemotaxis
GO:0030335	positive regulation of cell migration
GO:0070098	chemokine-mediated signaling pathway
GO:0048245	eosinophil chemotaxis
GO:2000503	positive regulation of natural killer cell chemotaxis
GO:0070374	positive regulation of ERK1 and ERK2 cascade
GO:0007159	leukocyte cell-cell adhesion
GO:0032755	positive regulation of interleukin-6 production
GO:0043922	negative regulation by host of viral transcription
GO:0051897	positive regulation of phosphatidylinositol 3-kinase/protein kinase B signal transduction
GO:0043410	positive regulation of MAPK cascade

Pathway ID	Pathway name
hsa05140	Leishmaniasis
hsa05323	Rheumatoid arthritis
hsa04640	Hematopoietic cell lineage
hsa05152	Tuberculosis
hsa04672	Intestinal immune network for IgA production
hsa05321	Inflammatory bowel disease
hsa05416	Viral myocarditis
hsa05150	Staphylococcus aureus infection
hsa04659	Th17 cell differentiation
hsa04658	Th1 and Th2 cell differentiation
hsa05142	Chagas disease
hsa04940	Type I diabetes mellitus
hsa05332	Graft-versus-host disease
hsa04062	Chemokine signaling pathway
hsa04145	Phagosome
hsa05310	Asthma
hsa05164	Influenza A
hsa05322	Systemic lupus erythematosus
hsa04061	Viral protein interaction with cytokine and cytokine receptor
hsa05330	Allograft rejection
hsa05145	Toxoplasmosis
hsa04514	Cell adhesion molecules
hsa04612	Antigen processing and presentation
hsa05166	Human T-cell leukemia virus 1 infection
hsa05163	Human cytomegalovirus infection
hsa05168	Herpes simplex virus 1 infection
hsa05320	Autoimmune thyroid disease
hsa05169	Epstein-Barr virus infection
hsa04060	Cytokine-cytokine receptor interaction
hsa04620	Toll-like receptor signaling pathway
hsa04670	Leukocyte transendothelial migration
hsa05133	Pertussis
hsa04380	Osteoclast differentiation
hsa05144	Malaria
hsa05146	Amoebiasis
hsa04064	NF-kappa B signaling pathway
hsa04623	Cytosolic DNA-sensing pathway

Supplementary Figure S6-3-7. GO and KEGG enrichment analysis of epithelial cells (clustering: subtype-specific marker, CTC subtype).

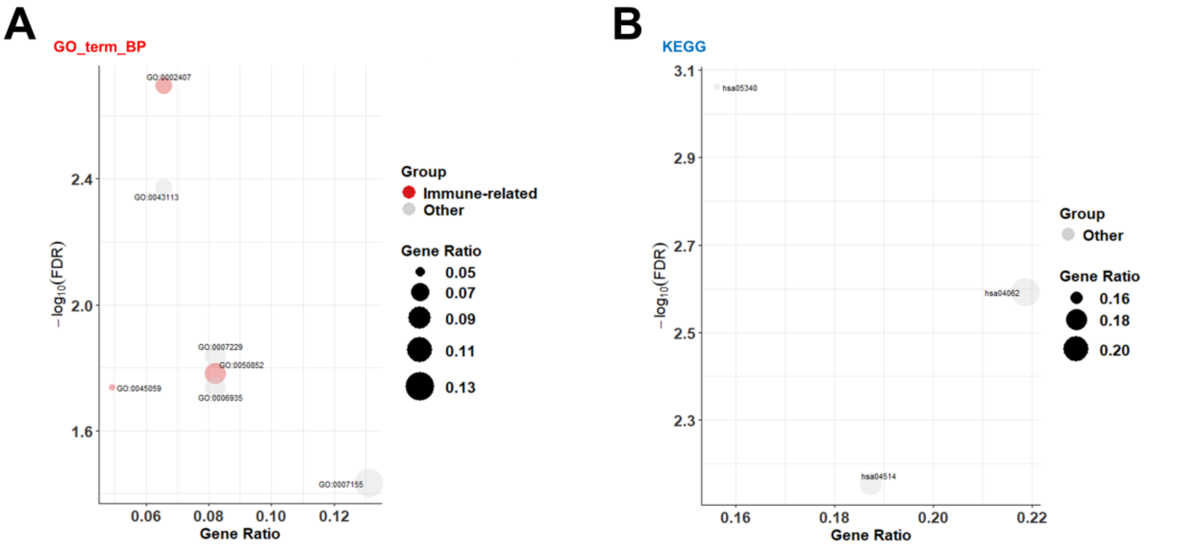

GO ID	GO term name
GO:0002407	dendritic cell chemotaxis
GO:0043113	receptor clustering
GO:0007229	integrin-mediated signaling pathway
GO:0050852	T cell receptor signaling pathway
GO:0006935	chemotaxis
GO:0045059	positive thymic T cell selection
GO:0007155	cell adhesion

Pathway ID	Pathway name
hsa05340	Primary immunodeficiency
hsa04062	Chemokine signaling pathway
hsa04514	Cell adhesion molecules

Supplementary Figure S6-3-8. GO and KEGG enrichment analysis of epithelial cells (clustering: subtype-specific marker, EMT subtype).

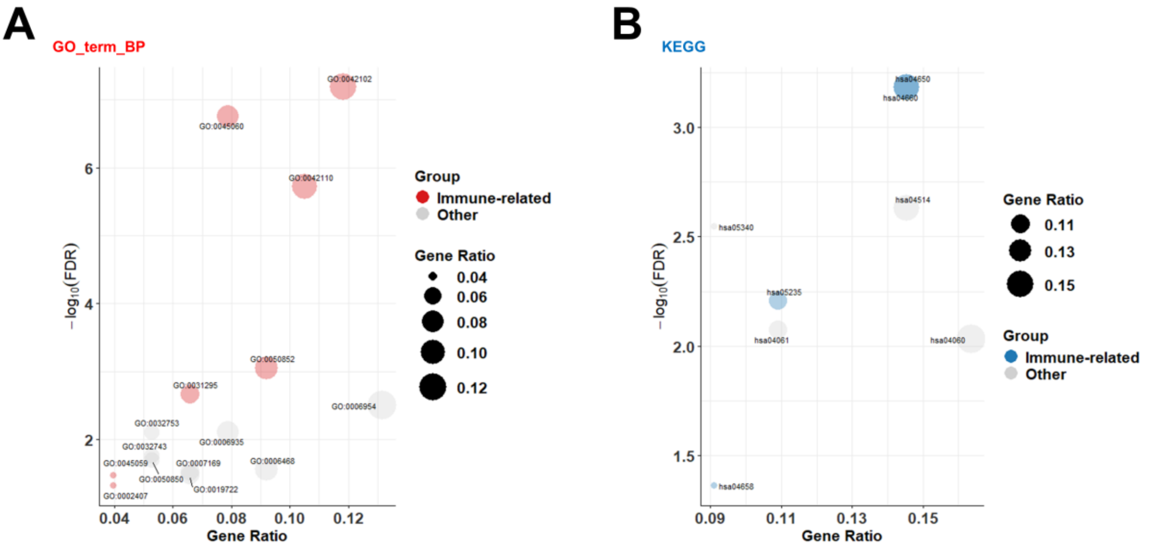

GO ID	GO term name
GO:0042102	positive regulation of T cell proliferation
GO:0045060	negative thymic T cell selection
GO:0042110	T cell activation
GO:0050852	T cell receptor signaling pathway
GO:0031295	T cell costimulation
GO:0006954	inflammatory response
GO:0032753	positive regulation of interleukin-4 production
GO:0006935	chemotaxis
GO:0032743	positive regulation of interleukin-2 production
GO:0050850	positive regulation of calcium-mediated signaling
GO:0006468	protein phosphorylation
GO:0007169	cell surface receptor protein tyrosine kinase signaling pathway
GO:0019722	calcium-mediated signaling
GO:0045059	positive thymic T cell selection
GO:0002407	dendritic cell chemotaxis

Pathway ID	Pathway name
hsa04650	Natural killer cell mediated cytotoxicity
hsa04660	T cell receptor signaling pathway
hsa04514	Cell adhesion molecules
hsa05340	Primary immunodeficiency
hsa05235	PD-L1 expression and PD-1 checkpoint pathway in cancer
hsa04061	Viral protein interaction with cytokine and cytokine receptor
hsa04060	Cytokine-cytokine receptor interaction
hsa04658	Th1 and Th2 cell differentiation

Supplementary Figure S6-3-9. GO and KEGG enrichment analysis of T cells (clustering: cell-to-cell type mapping).

Common description for Supplementary Figures S6-3-1 to S6-3-9:

Dot plots show enriched Gene Ontology biological process (GO_BP, left) terms and KEGG pathways (right) identified from upregulated genes in the indicated cell types under each clustering strategy. The x-axis represents the gene ratio, and the y-axis represents $-\log_{10}$(FDR). Dot size is proportional to the number of genes in each category, and dot color distinguishes immune-related categories (red for GO terms, blue for KEGG pathways) from other functional pathways (gray). Tables below each plot list the corresponding GO terms or KEGG pathways with their identifiers.

Legends of Supplementary Tables

Supplementary Table S1-1. Differentially expressed genes (DEGs) between KP_Pos and Other cells within each immune cell type based on SingleR majority-voting annotation (related to Figure 1D and Supplementary Figure S1-4).

Supplementary Table S1-2. Differentially expressed genes (DEGs) between KP_Pos and Other cells within each immune cell type based on cell-level annotation (related to Supplementary Figure S1-2-1).

Supplementary Table S1-3. Differentially expressed genes (DEGs) between KP_Pos and Other cells within each immune cell type based on cluster-to-cell-type mapping (related to Supplementary Figure S1-2-2).

Supplementary Table S1-4. Differentially expressed genes (DEGs) between KP_Pos and Other cells within each immune cell type based on CellTypist annotation (related to Supplementary Figure S1-2-3).

Supplementary Table S1-5. Differentially expressed genes (DEGs) between KP_Pos and Other cells within each immune cell type based on canonical marker–defined cell types (related to Supplementary Figure S1-2-4).

Supplementary Table S1-6. Differentially expressed genes (DEGs) between KP_Pos and Other cells within each immune cell type based on subtype-specific marker annotation (related to Supplementary Figure S1-2-5).

Supplementary Table S2-1. Summary of Cell Area Distribution (cumulative proportion values for each group).

Supplementary Table S2-2. Summary of Cytoplasm Area Distribution (cumulative proportion values for each group).

Supplementary Table S2-3. Global Statistics for Cell Area (overall mean and median values).

Supplementary Table S2-4. Global Statistics for Cytoplasm Area (overall mean and median

values).

Supplementary Table S3-1. Cluster-specific marker genes based on cell-level annotation (related to Supplementary Figure S3-1).

Supplementary Table S3-2. Cluster-specific marker genes based on SingleR majority voting annotation (related to Figure 3A-a and Supplementary Figure S3-2).

Supplementary Table S3-3. Cluster-specific marker genes based on cluster-to-cell type mapping (related to Supplementary Figure S3-3).

Supplementary Table S3-4. Cluster-specific marker genes based on CellTypist annotation (related to Supplementary Figure S3-4).

Supplementary Table S3-5. Cluster-specific marker genes based on canonical marker-defined cell types (related to Supplementary Figure S3-5).

Supplementary Table S3-6. Cluster-specific marker genes based on subtype-specific marker annotation (related to Supplementary Figure S3-6).

Supplementary Table S4-1-1. Distribution of KP_Pos and Other Cells Based on Cell-Level Annotation (Integration Across Benign, Primary, and Metastatic Samples).

Supplementary Table S4-1-2. Distribution of KP_Pos and Other Cells Based on Majority Voting Annotation (Integration Across Benign, Primary, and Metastatic Samples).

Supplementary Table S4-1-3. Distribution of KP_Pos and Other Cells Based on Cluster-to-Cell Type Mapping (Integration Across Benign, Primary, and Metastatic Samples).

Supplementary Table S4-1-4. Distribution of KP_Pos and Other Cells Based on CellTypist Annotation (Integration Across Benign, Primary, and Metastatic Samples).

Supplementary Table S4-1-5. Distribution of KP_Pos and Other Cells Based on Canonical Cell Type–Specific Marker Annotation (Integration Across Benign, Primary, and Metastatic Samples).

Supplementary Table S4-1-6. Distribution of KP_Pos and Other Cells Based on Subtype-Specific

Marker Annotation (Integration Across Benign, Primary, and Metastatic Samples).

Supplementary Table S4-2-1. Metastasis-Specific Marker Genes Based on Cell-Level Annotation (Integration Across Benign, Primary, and Metastatic Samples).

Supplementary Table S4-2-2. Metastasis-Specific Marker Genes Based on Majority Voting Annotation (Integration Across Benign, Primary, and Metastatic Samples).

Supplementary Table S4-2-3. Metastasis-Specific Marker Genes Based on Cluster-to-Cell Type Mapping (Integration Across Benign, Primary, and Metastatic Samples).

Supplementary Table S4-2-4. Metastasis-Specific Marker Genes Based on CellTypist Annotation (Integration Across Benign, Primary, and Metastatic Samples).

Supplementary Table S4-2-5. Metastasis-Specific Marker Genes Based on Canonical Cell Type–Specific Marker Annotation (Integration Across Benign, Primary, and Metastatic Samples).

Supplementary Table S4-2-6. Metastasis-Specific Marker Genes Based on Subtype-Specific Marker Annotation (Integration Across Benign, Primary, and Metastatic Samples).

Supplementary Table S5-1. List of Predictive Gene Signatures with Gene Composition and Three-Way Partitioning Results.

This table summarizes all constructed gene signatures used for M0/M1 classification. It includes signature ID, gene composition, and performance results from three-way data partitioning (training, validation, and test AUC scores) evaluated across five random seeds.

Supplementary Table S5-2. Classification Performance Metrics for Each Signature Using RF and XGB Models.

Evaluation scores (AUC, Accuracy, Sensitivity, Specificity, Precision, and F1 score) for each gene signature based on Random Forest (RF) and Extreme Gradient Boosting (XGB) models are summarized in this table.

Supplementary Table S5-3. Overfitting Level Classification for Each Gene Signature.

Overfitting level assigned to each gene signature based on the difference in PR_AUC between validation and test datasets across five random seeds. Signatures are categorized as None, Mild, Moderate, or Severe overfitting.

Supplementary Table S6-1-1. Composite scores of 119 individual signatures used for M-stage prediction (signatures categorized as 'None' in overfitting level assessment).

Supplementary Table S6-1-2. Prediction accuracy for M-stage classification using the top 55 high-performing signatures based on composite score.

Supplementary Table S6-2-1. Performance metrics of five signature combinations evaluated using Random Forest (RF) analysis.

This table summarizes the classification performance of five signature combinations generated from the 19 composite score–positive signatures identified in Figure 6B. The corresponding scatter plot is shown in Figure E-a, where the five evaluated combinations are highlighted in orange, illustrating the relationship between mean accuracy and mean ROC AUC using the RF model.

Supplementary Table S6-2-2. Performance metrics of 23 signature combinations evaluated using XGBoost (XGB) analysis.

This table summarizes the classification performance of 23 signature combinations generated from the same 19 composite score–positive signatures shown in Figure 6B. The corresponding scatter plot is presented in Figure E-b, where the 23 evaluated combinations are highlighted in orange, illustrating the relationship between mean accuracy and mean ROC AUC using the XGB model.

Supplementary Table S7-1. Cell type markers.

This table lists the marker genes used to define major cell types for annotation in this study.

Note: Although referenced earlier in the Materials and Methods section, the numbering is retained for consistency with the overall Supplementary numbering scheme.

Supplementary Table S7-2. Cell subtype markers.

This table lists the marker genes used to define functional cell subtypes within major cell types for annotation in this study.

Note: This table appears in the Materials and Methods section prior to other Supplementary materials; numbering has been preserved for consistency.
